# Pre-exposure Prophylaxis (PrEP) for HIV Prevention Among Men Who Have Sex with Men (MSM): A Scoping Review on PrEP Service Delivery and Programming

**DOI:** 10.1007/s10461-020-02855-9

**Published:** 2020-04-09

**Authors:** Alyson Hillis, Jennifer Germain, Vivian Hope, James McVeigh, Marie Claire Van Hout

**Affiliations:** Faculty of Health, Public Health Institute, Liverpool John Moores University, Exchange Station, Liverpool, L32ET UK

**Keywords:** Pre-exposure prophylaxis, PrEP, HIV prevention, Biomedical prevention products, Men who have sex with men, MSM

## Abstract

Pre-exposure prophylaxis (PrEP) is an evidence-based new biomedical HIV prevention intervention, which involves the pre-emptive use of daily (or event-based) antiretroviral drugs, to reduce risk of HIV acquisition if exposed. PrEP has recently been positioned as an integral prevention tool to reduce HIV acquisition risk among men who have sex with men (MSM) at country-level and within global prevention strategies. Given this global scale up of PrEP, we conducted a scoping review of extant international literature documenting service related perspectives, models and lessons learnt in PrEP programming for MSM. A systematic search of literature was conducted, and restricted to English language records in the timeframe 2008 to February 2019. Eligibility criteria centered on whether studies broadly described PrEP programming and service delivery for MSM as well as health communication. Following exclusion of ineligible records and removal of duplicates, 84 records were charted and thematically analysed according to scoping review methods. Four themes emerged from the thematic analysis of data; ‘*PrEP service aspects, settings and staff’;* ‘*PrEP prescriber experiences*, *therapeutic alliance and care planning*’; ‘*PrEP adherence within formal service structures*’; and ‘*Multi-disciplinary and innovative PrEP care pathways*’. The review highlights the complexities in providing optimal PrEP services for MSM by mapping and illustrating the importance of understanding the informal and formal routes to PrEP use among this HIV risk population; the barriers to uptake; the requirement for the presence of a positive therapeutic alliance between patient and prescriber in supporting patient initiation and adherence to PrEP regimes; and the need for availability in different culturally and ethnically sensitive models of PrEP service delivery according to low to high risk groups within the MSM communities.

**Electronic supplementary material:**

The online version of this article (10.1007/s10461-020-02855-9) contains supplementary material, which is available to authorized users.

## Background

In 2016, the United Nations General Assembly agreed that a fast-track response was required to end AIDS by 2030 and reduce new HIV infections to fewer than 500,000 annually by 2020 worldwide. The response is primarily through continued progress towards the 90–90–90 target (by 2020, 90% of all people living with HIV will know their HIV status, 90% of those diagnosed will receive antiretroviral therapy (ART) and 90% will have viral suppression) and through an intensive focus on people-centred implementation of the five prevention pillars [1–3]. The five prevention pillars are a combination prevention approach involving sexual education and economic empowerment to women, human rights programmes for key populations, condom programmes, voluntary medical male circumcision, and the use of pre-exposure prophylaxis (PrEP) [1].

The last few years has seen considerable breakthroughs in the prevention of new HIV infections [4, 5]. Yet transmission of HIV amongst men who have sex with men (MSM) in developed and developing countries remains a challenge and the reduction of the HIV disease burden among this key population is integral to ending the HIV epidemic [6–10]. Globally MSM are estimated to be at almost twenty times greater odds of acquiring HIV compared to the general population [11, 12]. Although undoubtedly important, ART alone will not reduce the epidemic enough to move towards elimination [13]. PrEP is an evidence-based biomedical HIV prevention intervention which involves the pre-emptive use of daily (or event-based) ARTs [tenofovir disoproxil fumurate (TDF) and emtricitabine (FTC)] to reduce the risk of HIV acquisition if exposed [14, 15]. PrEP has low toxicity and has been shown to be effective (particularly when adherence is high) among high risk groups including MSM [16–20]. Given its successes in efficacy, [21, 22] PrEP is now considered as an integral tool in the progressive strengthening of a combined HIV prevention programme among MSM, which includes 100% condom use, [23] voluntary HIV testing and counselling services, [24, 25] and HIV treatment as prevention (TasP) [26]. The roll out of PrEP along with continued support in testing and rapid access to treatment are the key drivers in the elimination of HIV and it is for this reason that the WHO and UNAIDS have prioritised PrEP implementation for populations at the highest risk of HIV [4, 27, 28].

Regulatory approval of PrEP in recent years has shifted international research activity towards PrEP demonstration projects, [15, 29] which aim to provide evidence around cultural and MSM sub-population variability in the acceptance of PrEP, [8, 30–33] associated stigma and interactions with healthcare providers, [34] understanding of cost and adherence, [35, 36] impact on sexual behaviour and “*risk compensation*”, [12, 37, 38] and development of drug resistance [39, 40].

To date, PrEP literature has focused on knowledge, awareness and willingness to use PrEP, [41–44] but as implementation trials are being carried out and PrEP is being made available, further evidence is required to identify suitable types of service delivery and programming for PrEP. There is now a need to establish how PrEP can optimally be embedded into existing combined HIV prevention programmes for MSM, in order to assert an effective and sustainable stand-alone regimen as well as an efficient combination model of service delivery. An important aspect of PrEP programming incorporates how services have been, and will be, communicated to MSM as well as healthcare providers. This will ensure a streamlined integration of PrEP into healthcare settings and ultimately a global change in attitudes and behaviour [45].

## Methods

There have been urgent calls to action from community organisations and charities directed at governments to ensure that once implementation trials end, which in England is relatively soon, there will be a seamless transition for patients to access PrEP [46]. The planning of PrEP programming, complemented by effective health communication and education, is therefore key to success. Due to this, a scoping review on PrEP was conducted in order to establish what is known about PrEP, with particular focus on the context of PrEP programming and service delivery for MSM. Scoping review methodologies are a valid approach and increasingly used across a variety of disciplines, particularly when a topic is not extensively reviewed, and where a comprehensive descriptive overview of available information across a wide range of study designs and methodologies is warranted [47–50]. It is a form of research synthesis that aims to ‘*map the literature on a particular topic or research area and provide an opportunity to identify key concepts; gaps in the research; and types and sources of evidence to inform practice, policymaking, and research’* [49]. The five stage iterative process developed by Arksey and O'Malley [48] was adhered to throughout this review process and consisted of the following key stages; (1) identifying the research question, (2) identifying relevant studies, (3) study selection, (4) charting the data and (5) collating, summarizing and reporting the results. The underpinning research question was: ‘*What is known about PrEP service delivery in terms of communication and form of PrEP consumer knowledge, and implementation within stand-alone or combination models of service delivery for MSM*?’.

Following an initial exploratory search, comprehensive searches were conducted in the Cochrane Database of Systematic Reviews, Medline, Web of Science, Scopus, PsycINFO and CINAHL to locate publications over a 10 year timeframe up to 2019. Key words and terms such as *‘PrEP*’, ‘*Pre-exposure prophylaxis*’, ‘*men who have sex with men*’ and *‘MSM*’ (Table 1) were used to locate empirical studies as well as grey literature (for example, international and national policy documents, thesis and online reports, PubMed Clinical Queries and Scopus). Manual searching of reference lists was undertaken. Eligibility criteria centered on whether studies broadly described PrEP programming and service delivery for MSM as well as health communication. Inclusion criteria therefore covered but was not limited to, prescribing, adherence, access, interventions, programming as well as structures and modelling. We restricted the search to records in the English language. Where possible we included PrEP user, MSM community and healthcare provider (nurses, community health workers, doctors, social and outreach worker) perspectives.

**Table 1:** Search terms and strategy

Search	Search terms	Results
1	PrEP OR “pre-exposure prophylaxis” OR tenofovir OR truvada OR emtricitabine OR “TDF" OR "FTC”	13,833
2	Homosexual* OR “men who have sex with men” OR MSM OR gay OR bisexual	51,188
3	1 + 2	1003

Databases were searched using the appropriate subject headings and/or keywords or text words for the above search groups. Example search (Medline) conducted on 03/08/2018

**Fig. 1:**
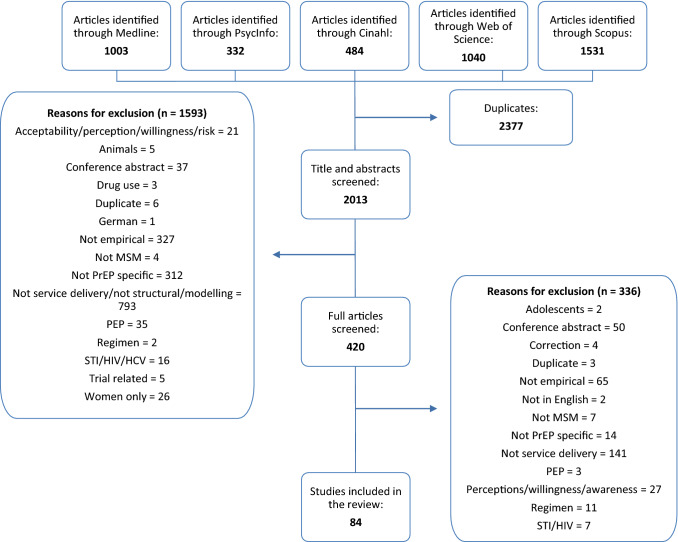
Flowchart for inclusion and exclusion of literature

Records were managed using EndNote, with duplicates removed manually by two members of the team. The title and abstract of each record were screened independently by two authors, cross checked by a third, and where any doubt remained in terms of inclusion, a fourth author reviewed the record [50]. All records deemed relevant on screening, underwent a second full-text screening to ensure relevance and eligibility for this scoping review. Searches identified 2013 unique records, and of these, 84 were selected according to inclusion criteria, charted and thematically analysed (Fig. 1). The charting exercise was conducted as per Levac et al. [50] by two members of the team in consultation, and generated specific themes pertaining to PrEP communication, delivery and programming of PrEP as either a stand-alone intervention, or as part of a combination HIV prevention intervention for MSM. An Excel spreadsheet charted all relevant data (including year of publication, author, method and aim, results, key findings and conclusion) to enable the identification of commonalities, themes and gaps in the extant literature. A trial charting exercise as recommended by Daudt et al. [49] was conducted in order to ensure consistency of approach and to facilitate the development of prior categories guiding the subsequent extraction and charting of the data from the records. Keywords and emerging themes were documented in parallel to the extraction of data. Once data extraction was completed, two authors discussed the findings and themes. As qualitative, quantitative and mixed methods studies were included in the review, the data extraction table was kept broad to ensure that all data were captured, adequately documented and thoroughly analysed. Where additional data extraction categories emerged, team consultation guided decisions around reporting of results. Four themes emerged from the thematic analysis of the collective records: *‘PrEP service aspects, settings and staff’; ‘PrEP prescriber experiences, therapeutic alliance and care planning’; ‘PrEP adherence within formal service structures’;* and *‘Multi-disciplinary and innovative PrEP care pathways’* (Supplementary Table S1)*.* These themes additionally reflect the service users’ chronological experience of the PrEP cascade [51]. Motivations for taking PrEP were not included here as they refer to the individual’s perceptions and awareness of PrEP, as opposed to their experiences of service delivery.

## Results

### Profile of Studies

The final sample consisted of 84 studies, the majority of which were empirical (Supplementary Table S2). While there was a mixture of quantitative (n = 49), qualitative (n = 29) and mixed methods (n = 6) studies, the majority (n = 62) had a cross sectional study design. Other methodological approaches were intervention evaluations (n = 8), cohort (n = 7), randomised control trials (n = 6) and ethnographic studies (n = 1). Although different methodological approaches were used, this was not an issue as similar recruitment methods were carried out across the pieces of research. This meant that the results could be appropriately charted and thematically analysed as per scoping review methodologies [49]. In the studies that targeted MSM (n = 70) as opposed to healthcare providers (n = 15), the main recruitment channels were through clinics and community health centres (n = 26), ongoing trials (n = 21), community organisations, groups and outreach (n = 11), local neighbourhoods and word of mouth (n = 11), social network websites (n = 11), social media (n = 10) and phone apps (n = 9).

The majority of research is from the United States (US; n = 69), with remaining papers based in Thailand (n = 5), South America (n = 4), the United Kingdom (UK; n = 3), South Africa (n = 3), Kenya, Canada, Germany, France (n = 2) and Australia, Ukraine and the rest of Europe (n = 1). From a global perspective, the US health system largely differs from other countries as it is predominantly funded by private health insurance with elements of public health coverage. Due to this disparity in the geographic reach of research, this review of extant PrEP service-related literature must be understood through the lens of limited access to free healthcare and the need to pay for potential PrEP prescriptions. Interestingly, the studies that focused on PrEP in Thailand, highlighted the more innovative approaches of service provision that focused on widespread distribution, scale up and novel interventions [52–54].

In terms of age and demographic, the included records focused on two key MSM subgroups, namely youth and Black/African American populations (Supplementary Table S2). Eleven studies focused on ‘young’ MSM aged between 16 and 25 years old; 47 studies addressed 26 to 40 year olds; four studies explored 41 year olds and above and seven targeted all age groups. Of the articles that targeted PrEP service users, 36% of records had a sample that was predominantly Black/African American/non-Hispanic, whilst 49% were White and only 15% had a Latino/Hispanic majority. Only 11% (n = 9) of the final articles directly focused on Black MSM and their specific needs.

### Theme One: PrEP Service Aspects, Settings and Staff

Theme One outlines current PrEP resource allocation and service delivery models that were reported in the studies. Three areas were found to be of interest within the papers: the specialty responsible for PrEP provision (also known as the ‘Purview Paradox’), the setting in which the services should be held and how these two elements operate together within a larger framework. Firstly, the ‘Purview Paradox’ was reported in a number of articles [55–64]. Some authors viewed sexual health workers as *‘first adopters’* in PrEP service delivery due to on-site expertise, [65] whilst others favored primary care staff as they are the first point of contact for high-risk patients [55, 65, 66]. Doblecki-Lewis [67] and Hoffman et al. [55] offered a unified approach, urging services to consider *‘task-shifting’* between primary care staff and HIV specialists. Alternatively, seven studies identified the potential role for ‘PrEP navigators’—individuals performing activities to assist potential and current PrEP users through bridging communications with relevant services through information provision and support [62, 68–73]. Additional training for service providers was noted as a necessity by 18 studies to ensure effective and sustainable PrEP delivery and programming, which could also provide a solution to bridging the gap between service provider knowledge and the prescribing of PrEP [55, 57, 59, 60, 63, 65, 67–69, 72–81].

Secondly, the scoping review highlighted the geographical constraints experienced by many of the study samples. Whilst 53 studies were based in cities, only two studies explored the impact of rural locations on accessing PrEP [53, 82]. The distance between the service user and provider dictated patients’ ability to access PrEP [82, 83]. For example, Hubach et al. [82] stated that more rural primary care providers did not prescribe PrEP as they believed it was out of their medical purview. Geographical barriers can have a significant impact on health outcomes, mostly effecting disadvantaged individuals [71]. To overcome these obstacles, Aloysius et al. [84] and Sun et al. [62] observed that pharmacies were geographically convenient for patients. However, some pharmacies experienced disruptions in the delivery of medication, with participants having to receive refills by post. Two studies discussed the possibility of home-based (HB) PrEP provision [56, 60]. John et al. [56] reported that nearly three-quarters (n = 655) of their sample, ‘*preferred to receive PrEP*…*care* via *HB-PrEP services*’, and it should therefore be considered as a potential avenue to increase PrEP uptake. Alongside other authors, [53, 55, 62, 65, 69, 70, 85, 86] Anand et al. [52] observed that PrEP uptake was greatest in community-based clinics (CBCs), such as Adam’s Love Clinic (70.4%) in Thailand and 56 Dean Street in London [84]. Although Arnold et al. [65] reported irregular access to doctors, poor laboratory monitoring and lack of follow-up at CBCs, in general they were seen to provide a broader, holistic approach to PrEP service provision by performing counselling, HIV and STI screening, tailored patient care and adherence support [52]. Furthermore, as CBCs are specialised, staff receive regular training opportunities and quality assessments to ensure that patients continue to receive high standards of care [52].

Finally, taking into consideration both staff and settings, recommendations regarding effective PrEP service delivery models were made in 37 studies. Of paramount importance was the design and implementation of a PrEP service, tailored to the patient’s needs [53, 87]. Uptake and adherence would then be reinforced by regular contact between the PrEP user and healthcare providers with regular screening and patient counselling [55, 88–92]. PrEP service implementation is underpinned by the necessity to reiterate the importance of taking PrEP as a commitment for protecting both the PrEP user and other people, [54, 92, 93] allowing for non-judgmental consultations, reporting missed dosages and providing ongoing education [67, 92].

### Theme Two: PrEP Prescriber Experiences, Therapeutic Alliance and Care Planning

MSM experiences of sexual health consultations were heavily reported in the included studies. The main areas covered were the relationship between the service user and service provider, topics discussed during consultation and service delivery recommendations. It is worth noting that only 18% (n = 15) of the studies interviewed service providers. Of these, the majority of service providers described positive attitudes towards PrEP prescribing with only two studies reporting that providers did not feel comfortable with current procedures [61, 76].

The reported descriptions of the service provider-user relationship ranged from an ‘*impersonal, heteronormative nature of *[*healthcare provider*] *interactions*’ [78] to the empathetic and professional efforts made by staff *‘to develop rapport across cultures’* [94]. Of the more negative experiences, stigma (related to homophobia, racism, lack of sensitivity, discrimination and inappropriate or offensive language) played a key role in the discussions between the patient and the service provider [62, 68, 78–80, 82, 83, 94–98]. Furthermore, Calabrese et al. [68] described the service provider’s disapproval of the users’ motivations for seeking PrEP and the perception of it as “*a gay man’s prevention tool*”.

A surprising finding was the lack of open discussion that was experienced during consultations. From the perspective of the service user, conversations around sexual behaviour were missed as they were seen to be difficult, [62, 67, 80] uncomfortable, [61, 97] or as a consequence of the service providers’ failure to take complete histories [56, 60, 68, 78, 79, 86, 96]. Some studies reported the lack of disclosure of sexual activity [80, 82, 99] and PrEP use [80, 82, 99], due to concerns for privacy and confidentiality, perceived stigma, fear of negative repercussions and embarrassment. When service users were able to disclose their previous and current sexual behaviours this was due to a ‘*positive rapport with staff*’ [55, 77, 82, 92, 100, 101]. Other conversation topics noted informal PrEP use, [102] missed pills, [92] risk reduction and counselling, [55, 62] testing, [86] HIV prevention, wider sexual health and sexual orientation, [62, 80, 81, 97] systemic barriers such as lack of primary care practices, adherence and medication refills, [62, 97] drug use [101] and lack of healthcare support [94, 103]. As has been shown, research evidences service provision and initiation of PrEP, however the discontinuation of PrEP was only reported in three studies [57, 63, 104]. Ending treatment, as within any therapy area, requires clear protocol, guidelines and communication.

A range of solutions for improving the relationship between the service users and providers were offered in the studies. These included having gay-friendly service providers and affirmative training, [77, 79, 80, 94, 100] improving long-term communication to build trust, [62, 67, 92, 94, 95, 101] developing patient-centred care, [92] increasing service provider knowledge and awareness of PrEP, [62, 96, 101] and encouraging wider health discussions [62, 78]. Key solutions included the routinisation of PrEP prescribing with electronic health record systems that could be used ‘*to identify potential PrEP candidates…and follow up-care*’, [62] and the delivery of PrEP using a shared decision-making approach between the patient and service provider, as proposed by Calabrese et al. [68] and Krakower et al. [57].

### Theme Three: PrEP Adherence Within Formal Service Structures

Promoting and supporting the adherence to PrEP regimens was an occurring theme across all studies. Adherence is crucial to efficacy of PrEP, and if poorly managed, it can result in a risk of HIV acquisition due to suboptimal levels of drug concentration [85]. While Daughtridge et al. [105] witnessed an increase in adherence from 79% at 16 weeks post initiation to 88% at 28 weeks, some studies noted that adherence reduced over time [53, 106].

Tellalian et al. [63] and Stekler et al. [107] identified that service level support was needed in order to improve correct use of PrEP, adherence and retention. Other barriers to PrEP adherence at service user level included medication concerns, for example the pill being too big or tasting unpleasant, [108] missing clinics, [109] forgetting to take medication [54, 75, 103, 110] and cost [111, 112]. Disruption in routine was also reported [54, 93, 103, 108] but Vaccher et al. [103] stated that this could be reduced by carrying spare medication for emergencies. Certain service user groups were identified as being at risk of low PrEP adherence, including those from ethnic minorities, [73, 91, 111] young MSM, [91] smokers, [111] and those with problematic alcohol or substance use, [91] all of which are exacerbated by systemic disadvantages within society or from an individual’s predisposition to risky behaviour.

Dosing aids such as building on existing pill taking routines, [92] mobile phone technologies, pill boxes, calendars, alarms [73, 89, 91–93, 108, 111–113] and matching PrEP dosages to daily routines, [93] such as taking PrEP with food or when brushing teeth, [88, 103] were reported to be effective in helping service users adhere to their regimen. Jaiswal et al. [96] stated that those who had been taking PrEP for a prolonged period of time no longer required the support of reminders to ensure adherence to PrEP. However, certain dosing aids were met with some resistance. Vaccher et al. [103] found that some service users perceived pill boxes as being for the elderly. This was supported by Elst et al. [88], who suggested that adherence support strategies may not work outside of a trial setting.

### Theme Four: Multi-Disciplinary and Innovative PrEP Care Pathways

Successful PrEP care pathways are underpinned by multi-disciplinary and innovative approaches. Within the chronology of an optimal PrEP service delivery model, these presented as health messaging and communication, referral into the pathway, support services and technology interventions once individuals had initiated PrEP. The multi-level implementation framework and system characteristics described by Beach et al. [83] and Galindo et al. [114], provided a combination model of PrEP service delivery. Beach et al. [83] looked at provision across *microsystem*, *mesosystem* and *macrosystem* levels, identifying the constituents of each level.

The *microsystem level* represents where individuals participate directly with their surroundings and should therefore include the following three key elements. Firstly, information should be provided on how to access PrEP to address current structural barriers and challenges, [52, 57, 68, 70, 73, 85, 94, 97, 101, 108, 114–117] such as cost, [113, 118, 119] geography, [83] and stigma [78, 82, 120]. Secondly, general education about PrEP should be made accessible across a range of media, to a broad geographic and culturally diverse audience. The literature shows that aside from the regular methods of communication, PrEP messaging was effectively delivered through specialised PrEP educators and navigators, [65, 72, 93, 114, 120–123] the LGBT community [68, 73, 80, 83] and PrEP hotlines [73]. These communication methods were delivered through various interventions such as outreach work, [73, 78, 122] behaviour changing strategies [82] and stigma-specific campaigns [61, 73, 77]. Community mobilisation interventions were particularly effective as they created*, ‘social change by building awareness of critical health issues*…*empower[ing] community members to take charge of their healthcare needs through a collective, engaging and iterative process*…[*therefore it*] *has been effective in other population-level HIV prevention efforts*’ [114]. Although much of the literature discussed the positive impact that tailored and reframed messaging has had on the uptake of PrEP, [56, 78, 96, 98, 114] 22 studies identified the need for greater levels of communication and education [59, 61, 65, 67, 72, 73, 77–80, 82, 88, 92, 93, 101, 102, 117, 120, 122, 124–126]. For example, Calabrese et al. [68] stated that there was a gap for ‘*the development of curricula and evaluation standards for PrEP-related medical education*’, whilst Newman et al. [120] highlighted the importance of online experiences in bringing together the gay and bisexual male (GBM) PrEP community. The third element is the identification of potential PrEP users [52, 61, 73, 96, 108, 127]. This could be through existing relationships between the individuals and healthcare providers or counsellors but also through established referral systems. A clear referral route into the PrEP care pathway was highlighted in 14 studies [53, 55, 57, 61, 62, 66, 68, 69, 73, 79, 83, 101, 115, 117, 123]. Pathways may need to accommodate a number of referral routes, including online platforms, testing services, LGBT services, self-referrals (such as word of mouth, dating apps and online search engines), peer and community-based organisations, primary care practices or general practices, other departments (such as health, infectious disease, sexual health, urgent care or emergency), social organisations, medical agencies as well as by transitioning patients who were previously prescribed post-exposure prophylaxis (PEP) and through partners seen at HIV or sexual health clinics. Anand et al. [52] described an online-to-offline (O2O) model that connects high risk MSM to PrEP and HIV testing services, counselling, information and administrative services. Other elements of the delivery model within the PrEP care pathway would benefit from improving these aspects of PrEP programming. This framework could subsequently help to reduce stigma, encourage uptake and adherence, give confidence, reaffirm decisions, rectify misinformation, better patient-provider relationships and ultimately provide open communication and optimum care for the patient.

The *mesosystem level* is the cross-disciplinary collaboration and interaction of service providers to MSM. Seven studies found that patients sought an environment in which providers were comfortable discussing sexual history and orientation, a place that was free from stigma and discrimination [52, 55, 56, 65, 82, 105, 120]. There is a need for the service provider to ensure the maintenance of confidentiality and therefore instil trust in the relationship, which will in turn encourage uptake and adherence to PrEP [57, 58, 61, 68, 72, 73, 78, 83, 94, 103, 108, 114, 120]. Support services that constitute current PrEP pathways were identified in 21 studies. Outside of routine PrEP care, services included LGBT-friendly support, [67] social support including mental health, homelessness and substance use, [65, 128] prevention and treatment of unrelated diseases, [128] sexual health education, [52, 55, 78, 118] vaccine administration, [69, 105] pharmaceutical patient assistance programs and outreach programs [67, 78, 88]. 15 studies focused on the positive impact of counselling or discussion-based services [52, 54, 62, 65, 66, 72, 73, 79, 87, 88, 91, 92, 96, 108, 129]. Providing these services, particularly with certain demographics such as adolescents and ethnic minorities, proved to increase adherence and uptake [65, 66, 88, 91, 92, 108]. Technology-based interventions were reported in five studies and they can be viewed as uniting the *micro-* and *mesosystem levels*. These technologies were considered to be useful for service users and providers for setting reminders, [92, 103, 106, 112, 130] monitoring and surveillance (particularly those with a high risk of contracting HIV), [52, 91, 112] education and information, [82, 102, 106, 130] tailoring care, [102] providing counselling, [52, 66, 77, 107, 131] adherence, [53, 67, 73, 79, 89, 91–93, 103, 106, 111, 112, 131, 132] recording dosing regimen (event-based, intermittent or daily), [93, 106, 112, 132] allowing individuals to feel connected and even providing maps that show where PrEP is offered in the local area [106, 133]. In their studies, Refugio et al. [130], Stekler et al. [107] and Fuchs et al. [89] implemented a text-based support strategy, which allowed participants to send messages, set reminders, download and receive information, abate stigma and enable staff to provide additional support throughout the PrEP user’s journey. Fuchs et al. [89] found that messages sent by service providers earlier in the week received better response rates from both PrEP users and other staff members. Liu et al.’s [106] PrEPmate included weekly ‘*check-in’* messages, reminders and fun facts or trivia for users, with 92% of participants recommending the intervention to others. Mitchell et al.’s [112] mSMART intervention app for adherence enabled users to log their dosing, take part in daily surveys (with a 70% completion rate) and take pictures to consolidate memories of taking the medication. In general, bi-directional and two-way messaging services were positively received by participants [89, 91, 93, 103, 106].

Lastly, the *macrosystem level* identifies the broad socio-cultural dynamics of PrEP users in society and ensures ‘*comprehensive services are available to all individuals*’ [83]. The comprehensive service utilises six main factors. These include the initial assessment of the potential PrEP user [52, 73, 96, 113]; tailoring of services to the individual receiving them [52, 54, 56, 57, 60, 65, 68, 79, 82, 83, 87, 88, 93, 94, 113–115, 117, 120]; triaging of services to ensure efficiency, for example point of care access, ‘*on demand’* or same day PrEP initiation [57, 94, 113, 115, 117]; ongoing relationship between the PrEP provider and the individual, which also includes shared-decision making processes [57, 61, 78, 93]; availability of resources such as onsite support staff, PrEP navigators or ‘*take-away*’, home-based PrEP services [56, 60, 68, 73, 88, 101, 103]; and ongoing support throughout the duration of the service [53, 58, 72, 73, 78, 82, 85, 87, 88, 90, 93, 94, 96, 101, 103, 108, 114–116, 120, 124, 127, 132]. If these elements are successfully integrated at a *macrosystem level*, then not only will the PrEP care pathway provide a holistic service but it will effectively cut across and encompass all patient groups regardless of different demographics or backgrounds.

### PrEP Service Delivery Model

**Fig. 2:**
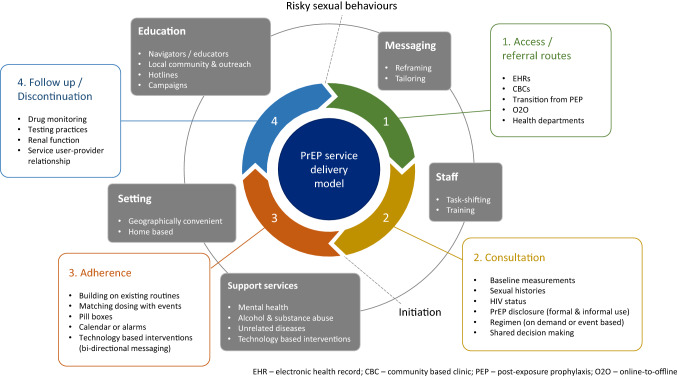
PrEP service delivery model

Based on the findings of the review, with particular consideration to Newman et al.’s [120] augmented PrEP cascade diagram, as well as in alignment to the multi-level framework and system characteristics presented by Beach et al. [83] and Galindo et al. [114] respectively, the authors have developed a PrEP service delivery model (Fig. 2). The model combines key service delivery aspects that were identified, evaluated and proven to be effective or positively received in the studies.

The inner cycle of the model highlights the four main touch points between the service user and the PrEP care pathway. The touch points are in the recommended order that will establish an effective patient pathway, according to the findings of the scoping review: access/referral routes (how the patient enters the PrEP pathway); consultation (key topics that should be discussed between the patient and the service provider); adherence (long-term pathway to ensure PrEP maintenance); and follow up/discontinuation (steps in place to enforce effective treatment).

The outer circle represents the context in which the four touch points function. These contextual factors operate throughout the PrEP care pathway in order for the overall model to perform effectively. These factors include education and messaging (through a tailored delivery using skilled PrEP navigators); task-shifting between primary care providers and HIV specialists; geographically convenient distribution and access to PrEP (also in the form of home-based delivery); and ongoing support services, complemented through innovative technological interventions.

Two actions have been included in the model for completeness. Although the PrEP service delivery model is continuous, the cycle is initiated once the patient starts displaying risky sexual behaviours. It is at this point that the patient would enter a health system and therefore the cycle (between touch points 4 and 1). PrEP should be initiated between touch points 2 and 3, during or after the initial consultation, depending on local resource allocation.

## Discussion

The scoping review presents a unique mapping of extant literature on the PrEP service care pathway, which can be used collectively to inform technical guidance in optimising PrEP provision within STI prevention programming for MSM. It highlights the complexities involved in optimising PrEP service uptake and service delivery, including the role of staffing, PrEP provider experience, setting, communications and service configuration for MSM. We recognise the limitations of such a review at a time when PrEP is being rolled out, with data restricted to the US, Thailand, UK, Canada, Kenya, Ukraine, Australia, France, Peru, Europe and Central Asia. Strengths centre on the thoroughness of the review approach through extensive searches to locate all forms of information with regard to PrEP service aspects and programming.

Theme One discusses the importance of resource allocations amongst staff and across various settings. Once these elements are established, patients should ideally have clear access to the PrEP care pathway, as can be seen in the PrEP service delivery model (Fig. 2). However, the review occurs at a time where online sourcing of drugs and pharmaceutical medicines is increasingly common. As many countries do not currently offer PrEP or restrict access, PrEP users can source the PrEP drugs informally via diverted medication and online [70, 72, 99, 102, 103, 126, 129]. Studies have also described the accessing of PrEP through deceit, either by pretending they have been exposed to HIV and are planning to use PEP as PrEP, [100] or pretending to be living with HIV [129]. In some instances this is a cost related decision where use of prescribed PrEP occurs either ‘*on-demand or event-driven’* in order to save money, [103, 113] where MSM are initiated to use PrEP by a sexual partner for *‘extra security’*, [129] or where MSM simply felt confident using informal PrEP as a pharmaceutical product if accessed through friends [102]. This has implications for technical service aspects of PrEP programming as users of informal PrEP report feeling guilty in deceiving their healthcare providers and fear judgement [129]. In addition, service configurations are hindered by the disconnect between healthcare providers who recommend PrEP but are unable to prescribe it, [72] the complexities between sexual risk and the uncertain efficacy of ‘*on-demand*’ use, [72] use of PrEP outside of engagement with medical and sexual health support in the form of STI screening and minimisation of PrEP related harm through kidney function tests, [99, 102] and general lack of PrEP user knowledge concerning drug resistance, adverse effects, STI exposure and toxicity [130]. These complexities highlight the need for a comprehensive list of programme considerations and recommendations across a range of services to support potential and existing PrEP service users along the care pathway. In particular there were mixed interpretations regarding the eligibility criteria and reasons for PrEP discontinuation, the necessity to obtain baseline measurements of patients during the initial consultation and report on these during follow up visits, as well as document and build upon the existing and emerging referral routes of patients. By standardising key processes within the wider system, this will allow for a more effective service through attainable resource allocation, thus providing a smoother, more robust programme delivery of PrEP, as can be seen in the PrEP service delivery model.

Two main aspects of service delivery have been brought to light during the review. The first is ensuring that PrEP candidates are able to access the system through effective referral routes and go on to receive suitable services that meet their needs. Mixed evidence exists with regard to PrEP and risk compensation, with the concept that PrEP use increases condomless sexual practices and STIs, which in turn undermines the positive aspects of PrEP and impacts on PrEP rollout among MSM [38, 134–142]. However, the review underscores the importance of PrEP to MSM service users and how it should be considered as a commitment to protect oneself and others [54, 92, 108]. Furthermore, PrEP provision in a service creates the opportunity for MSM to access sexual health care, testing, treatment, counselling regarding condom use and STI testing and psychological support that would not be accessed otherwise [12, 60]. Conversely, PrEP could also be considered and used as a gateway for individuals to engage in other health services that they may not have wanted to initially, for example with mental health and vaccination services [61]. This has been highlighted in touch point 4 of the PrEP service delivery model. Hence the review has brought to the fore the importance of exploring a wide range of potential access routes to PrEP by including both diverse referral routes and services that can offer PrEP to MSM [55, 56, 60, 62] as well as showing that a wide range of healthcare providers can be utilised to prescribe PrEP within the desired safe spaces free from stigma and discrimination [52, 55, 56, 65, 82, 105, 120]. The outer circle and touch point 2 of the PrEP service delivery model addresses these elements. Prescriber bias in determining eligibility can be reduced by the implementation of electronic health record systems to flag up potential PrEP candidate and follow up-care [62] as well as through shared decision-making [57, 68]. Distribution of PrEP by qualified prescribers who have developed positive therapeutic alliances with their patients based on a shared decision-making approach underpins adherence to correct treatment regimens, [75] reduction of stigma, [62, 78–80, 82, 96, 97] regular baseline assessments of STI rates, [99] risk behaviours, [62, 81, 95, 96] drug monitoring (TDM) and renal function [72, 84]. Once established, the PrEP care pathway can then be further supported by PrEP navigators to assist potential and current PrEP users through bridging communications, building trust and providing necessary information between users and relevant services [62, 67, 73, 92, 94, 95, 101], as shown in the outer circle of the PrEP service delivery model. Studies indicate the need for task shifting between primary care staff and HIV specialists as well as further training across all roles and professions in order to leave no one behind and facilitate referrals to streamline the PrEP cascade [55, 57, 59–61, 67, 69, 72, 75–77, 79, 80, 94, 100]. By adapting healthcare providers’ education and communication to target populations, a healthier rapport can be maintained with the patient, ultimately leading to better health outcomes.

The second point to be considered regards technical issues around supporting adherence to PrEP. The review highlights barriers to uptake and how adherence to PrEP regimens remains a fundamental component for consideration of programme planning, budget and PrEP effectiveness, alongside consideration of strategically situating PrEP interventions as stand-alone, and within, existing prevention programmes [143]. This is highlighted in touch point 3 of the PrEP service delivery model. Service aspects required to boost adherence are centered on a tailored approach, which are dependent on patient needs, [53, 87] regular education, [70, 92] regular contact between the PrEP user and healthcare providers with frequent screening, renal function testing, TDM and patient counselling, [55, 84, 88–92, 105, 111] all with a non-judgmental attitude [92]. There is a need for continued innovation, mindful of individual need and development of personalised engagement and adherence strategies. Innovations include dosing aids using mobile phone technologies, [53, 89, 92, 106] pill boxes, [73, 103] calendars, [73, 93, 103, 108] existing drug routines/events, [88, 89, 92, 103, 108] alarms to assist MSM patients and the option to carry spare medication [66, 67, 73, 92, 103, 108, 113]. These elements have been highlighted in touch point 3 of the PrEP service delivery model. Ultimately, there is a continued need to deepen our understanding of the biomedical, social and risk complexities of PrEP as a HIV prevention strategy for low to high risk MSM groups, and through a culturally sensitive approach. This is required in order to identify those in need and engage with them whilst mitigating stigma, [134, 144] determining eligibility, [143] and promoting self-assessment to ensure adequate adherence [57, 143].

### Future Research Directions

In finer detail, although socio-economic demographic data was captured in studies, this was not a focal point of discussion throughout the articles and represents an area for further investigation. The majority of studies state that there is a need for further research into black and ethnic minorities’ access to PrEP service provisions. Additionally, young MSM require further investigation as they are less likely to be seeking HIV/STI testing, diagnosis or treatment for HIV [145]. While Amico et al. [108], Desrosiers et al. [66] and Refugio et al. [130] discuss interventions and techniques to help increase the uptake of PrEP among youths, namely through technology and apps, considerably more research needs to be done. At service level, the majority of service providers described positive attitudes towards PrEP prescribing with only two studies reporting that providers did not feel comfortable with the current procedures [61, 76]. However, only 15 studies targeted healthcare providers. This highlights a major gap in the knowledge base with considerable more research needed to investigate the perspectives of healthcare professionals in the context of PrEP delivery. There is a clear and urgent need for research to outline a ‘*universal best approach*’ for follow-up and termination consultations [57, 104].

## Conclusion

The review highlights the complexities in providing optimal PrEP services for MSM. Environments free from stigma are important to the success of all aspects of PrEP delivery. Service aspects are underpinned by the need to understand informal and formal routes of PrEP use among MSM, understanding their barriers to uptake and retention, the importance of a positive therapeutic alliance between patient and prescriber in supporting patient initiation and adherence to PrEP regimes as well as the need for PrEP availability through different models of service delivery which are adapted to the MSM community and the providers involved. Findings here can be used to inform PrEP technical guidance to enhance programming across a range of modes of service delivery, in improving targeting low to high risk groups within the MSM communities, and in improving supporting medication adherence and STI screening. We make recommendations for future research directions as PrEP services are initiated and up-scaled globally.

## Electronic supplementary material

Below is the link to the electronic supplementary material.

Supplementary file1 (DOCX 60 kb)

Supplementary file2 (DOCX 43 kb)

## Data Availability

The datasets used and/or analysed during the current study are available from the corresponding author on reasonable request.

## References

[1] UNAIDS. Prevention Gap Report. 2016c. https://www.unaids.org/sites/default/files/media_asset/2016-prevention-gap-report_en.pdf. Accessed Jan 2019.

[2] UNAIDS. Strategy on the Fast Track to end AIDS 2016–2021. 2016d. https://www.unaids.org/sites/default/files/media_asset/20151027_UNAIDS_PCB37_15_18_EN_rev1.pdf Accessed Jan 2019.

[3] UNAIDS. 90–90–90: An ambitious treatment target to help end the AIDS epidemic. 2019. https://www.unaids.org/en/resources/909090. Accessed June 2019.

[4] AIDSMAP. The UK is moving towards elimination of HIV transmission. 2017. https://www.aidsmap.com/The-UK-is-moving-towards-elimination-of-HIV-transmission/page/3209286/. Accessed July 2019.

[5] UNAIDS. Global AIDS update. 2016a; Available at: https://www.unaids.org/en/resources/documents/2016/Global-AIDS-update-2016. Accessed June 2019.

[6] CDC. HIV among gay and bisexual men. 2015. https://www.cdc.gov/nchhstp/newsroom/docs/factsheets/cdc-msm-508.pdf. Accessed Jan 2019.

[7] Chris B, Andrea LW, Damian W et al. The global HIV epidemics in men who have sex with men. The International Bank for Reconstruction and Development. 2011.

[8] Peng P, Su S, Fiarley C (2018). Global estimate of the acceptability of pre-exposure prophylaxis for HIV among men who have sex with men: a systematic review and meta-analysis. AIDS Behav.

[9] NCAIDS, NCSTD, China CCD. Update on the AIDS/STD epidemic in China and main response in control and prevention in December, 2015. China J AIDS STD. 2016;22(2):69. http://caod.oriprobe.com/articles/47727492/Update_on_the_AIDS_STD_epidemic_in_China_and_main_response_in_control_.htm. Accessed Jan 2019.

[10] UNAIDS. The Gap Report 2014. 2014. https://files.unaids.org/en/media/unaids/contentassets/documents/unaidspublication/2014/UNAIDS_Gap_report_en.pdf. Accessed June 2019.

[11] UNFPA. Implementing comprehensive HIV and STI programmes with men who have sex with men: practical guidance for collaborative interventions. 2015. https://www.unfpa.org/sites/default/files/pub-pdf/MSMIT_for_Web.pdf. Accessed Aug 2019.

[12] Freeborn K, Portillo CJ (2018). Does preexposure prophylaxis for HIV prevention in men who have sex with men change risk behaviour? A systematic review. J Clin Nurs.

[13] Stover J, Bollinger L, Izazola JA (2016). What is required to end the AIDS epidemic as a public health threat by 2030? The cost and impact of the fast-track approach. PLoS ONE.

[14] Davies O (2016). Pre-exposure prophylaxis for hiv prevention: why, what who and how. Infect Dis Ther.

[15] WHO. Guidance on pre-exposure oral prophylaxis (PrEP) for serodiscordant couples, men and transgender womenwho have sex with men at high risk of HIV: Recommendations for use in the context of demonstration projects. 2012. https://apps.who.int/iris/bitstream/handle/10665/75188/9789241503884_eng.pdf;jsessionid=AB43EE2B0490DA58A6109943FA6B2022?sequence=1. Accessed June 2019. 23586123

[16] Grant RM, Lama JR, Anderson PL (2010). Preexposure chemoprophylaxis for HIV prevention in men who have sex with men. N Engl J Med.

[17] Liu A, Cohen S, Vittinghoff E. Adherence, sexual behavior and HIV/STI incidence among men who have sex with men (MSM) and transgender women (TGW) in the US PrEP demonstration (Demo) project. In: 8th International AIDS Society Conference on HIV Pathogenesis, Treatment, and Prevention. Vancouver, 2015.

[18] Molina JM, Capitant C, Spire B (2015). On-demand preexposure prophylaxis in menat high risk for HIV-1 infection. N Engl J Med.

[19] McCormack S (2016). Pre-exposure prophylaxis to prevent the acquisition of HIV-1 infection (PROUD): effectiveness results from the pilot phase of a pragmatic open-label randomised trial. Lancet.

[20] Trager M, Schroeder S, Wright E (2018). Effects of pre-exposure prophylaxis for the prevention of human immunodeficiency virus infection on sexual risk behavior in men who have sex with men: a systematic review and meta-analysis. Clin Infect Dis.

[21] Anderson PL, Glidden DV, Liu A (2012). Emtricitabine-tenofovir concentrations and pre-exposure prophylaxis efficacy in men who have sex with men. Sci Transl Med.

[22] Baeten JM, Grant R (2013). Use of antiretrovirals for HIV prevention: what do we know and what don't we know?. Curr HIV/AIDS Rep.

[23] Charania MR, Crepaz N, Guenther-Gray C (2011). Efficacy of structural-level condom distribution interventions: a meta-analysis of U.S. and international studies 1998–2007. AIDS Behav.

[24] Ford N, Irvine C, Shubber Z (2014). Adherence to HIV postexposure prophylaxis: a systematic review and meta-analysis. AIDS.

[25] Nugroho A, Erasmus V, Zomer TP, Wu Q, Richardus JH (2017). Behavioral interventions to reduce HIV risk behavior for MSM and transwomen in Southeast Asia: a systematic review. AIDS Care.

[26] Templeton DJ, Millett GA, Grulich AE (2010). Male circumcision to reduce the risk of HIV and sexually transmitted infections among men who have sex with men. Curr Opin Infect Dis.

[27] WHO. Guideline on when to start antiretroviral therapy and on pre-exposure prophylaxis for HIV. 2015. https://apps.who.int/medicinedocs/documents/s22247en/s22247en.pdf. Accessed June 2019. 26598776

[28] WHO. Consolidated guidelines on the use of antiretroviral drugs for treating and preventing HIV infection. Recommendations for a public health approach, second edition. 2016. http://www.who.int/hiv/pub/arv/arv-2016/en/. Accessed Jan 2019. 27466667

[29] WHO. PrEP demonstration projects: a framework for country level protocol development. 2013. https://apps.who.int/iris/handle/10665/112799. Accessed June 2019.

[30] Ding Y, Yan H, Ning Z (2016). Low willingness and actual uptake of pre-exposure prophylaxis for HIV-1 prevention among men who have sex with men in Shanghai. China Biosci Trends.

[31] Holt M (2014). HIV pre-exposure prophylaxis and treatment as prevention: a review of awareness and acceptability among men who have sex with men in the Asia-Pacific region and the Americas. Sex Health.

[32] Kirby T, Thornber-Dunwell M (2014). Uptake of PrEP for HIV slow among MSM. Lancet.

[33] Young I, McDaid L (2014). How acceptable are antiretrovirals for the prevention of sexually transmitted HIV?: a review of research on the acceptability of oral pre-exposure prophylaxis and treatment as prevention. AIDS Behav.

[34] Underhill K, Morrow KM, Colleran C (2015). A qualitative study of medical mistrust, perceived discrimination, and risk behavior disclosure to clinicians by U.S. male sex workers and other men who have sex with men: implications for biomedical HIV prevention. J Urban Health.

[35] Amico KR, Stirratt MJ (2014). Adherence to preexposure prophylaxis: current, emerging, and anticipated bases of evidence. Clin Infect Dis.

[36] Zhou F, Gao L, Li S (2012). Willingness to accept HIV pre-exposure prophylaxis among Chinese men who have sex with men. PLoS ONE.

[37] Blumenthal J, Haubrich R (2014). Risk compensation in PrEP: an old debate emerges yet again. Virtual Mentor.

[38] Marcus JL, Glidden DV, Mayer KH (2013). No evidence of sexual risk compensation in the iPrEx trial of daily oral HIV preexposure prophylaxis. PLoS ONE.

[39] Fonner VA, Dalglish SL, Kennedy CE (2016). Effectiveness and safety of oral HIV preexposure prophylaxis for all populations. AIDS.

[40] Mugwanya KK, Baeten JM (2016). Safety of oral tenofovir disoproxil fumarate-based pre-exposure prophylaxis for HIV prevention. Expert Opin Drug Saf.

[41] Eaton L, Driffin D, Smith H (2014). Psychosocial factors related to willingness to use pre-exposure prophylaxis for HIV prevention among Black men who have sex with men attending a community event. Sex Health.

[42] Grov C, Whitfield T, Rendina H, Ventuneac A, Parsons JT (2015). Willingness to Take PrEP and potential for risk compensation among highly sexually active gay and bisexual men…pre-exposure prophylaxis. AIDS Behav.

[43] Holloway I, Tan D, Gildner J (2017). Facilitators and barriers to pre-exposure prophylaxis willingness among young men who have sex with men who use geosocial networking applications in California. AIDS Patient Care STDs.

[44] Klevens R, Martin B, Doherty R (2018). Factors associated with pre-exposure prophylaxis in a highly insured population of Urban men who have sex with men, 2014. AIDS Behav.

[45] Rimal RN, Lapinski MK (2009). Why health communication is important in public health. Bull World Health Organ.

[46] Trust TH. Make PrEP available. 2019. https://www.tht.org.uk/our-work/our-campaigns/make-prep-available. Accessed Oct 2019.

[47] Anderson S, Allen P, Peckham S, Goodwin N (2008). Asking the right questions: scoping studies in the commissioning of research on the organisation and delivery of health services. Health Res Policy Syst.

[48] Arksey H, O’Malley L (2005). Scoping studies: towards a methodological framework. Int J Soc Res Methodol.

[49] Daudt H, van Mossel C, Scott S (2013). Enhancing the scoping study methodology: a large, inter-professional team’s experience with Arksey and O’Malley’s framework. BMC Med Res Methodol.

[50] Levac D, Colquhoun H, O’Brien KK (2010). Scoping studies: advancing the methodology. Implement Sci..

[51] Brouwers, P. Improving the HIV Pre-Exposure Prophylaxis (PrEP) Cascade. 2015. https://www.nimh.nih.gov/funding/grant-writing-and-application-process/concept-clearances/2015/improving-the-hiv-pre-exposure-prophylaxis-prep-cascade.shtml. Accessed Oct 2019.

[52] Anand T, Nitpolprasert C, Trachunthong D (2017). A novel online-to-offline (O2O) model for pre-exposure prophylaxis and HIV testing scale up. J Int AIDS Soc.

[53] Phanuphak N, Sungsing T, Jantarapakde J (2018). Princess PrEP program: the first key population-led model to deliver pre-exposure prophylaxis to key populations by key populations in Thailand. Sex Health.

[54] Tangmunkongvorakul A, Chariyalertsak S, Amico RK (2013). Facilitators and barriers to medication adherence in an HIV prevention study among men who have sex with men in the iPrEx Study in Chiang Mai Thailand. AIDS Care.

[55] Hoffman S, Guidry JA, Collier KL (2016). A clinical home for preexposure prophylaxis: diverse health care providers' perspectives on the "purview paradox". Int Assoc Provid AIDS Care.

[56] John SA, Rendina HJ, Grov C, Parsons JT (2017). Home-based pre-exposure prophylaxis (PrEP) services for gay and bisexual men: an opportunity to address barriers to PrEP uptake and persistence. PLoS ONE.

[57] Krakower DS, Ware NC, Maloney KM (2017). Differing experiences with pre-exposure prophylaxis in boston among lesbian, gay, bisexual, and transgender specialists and generalists in primary care: implications for scale-up. AIDS Patient Care STDs.

[58] Mullins TLK, Zimet G, Lally M (2017). HIV care providers' intentions to prescribe and actual prescription of pre-exposure prophylaxis to at-risk adolescents and adults. AIDS Patient Care STDs.

[59] Ojile N, Sweet D, Kallail KJ (2017). A preliminary study of the attitudes and barriers of family physicians to prescribing HIV preexposure prophylaxis. Kans J Med.

[60] Parsons JT, John SA, Whitfield THF, Cienfuegos-Szalay J, Grov C (2018). HIV/STI counseling and testing services received by gay and bisexual men using pre-exposure prophylaxis (PrEP) at their last PrEP care visit. Sex Transm Dis.

[61] Patel RR, Chan PA, Harrison LC (2018). Missed opportunities to prescribe HIV pre-exposure prophylaxis by primary care providers in Saint Louis Missouri. LGBT Health.

[62] Sun C, Anderson K, Bangsberg D (2019). Access to HIV pre-exposure prophylaxis in practice settings: a qualitative study of sexual and gender minority adults’ perspectives. J Gen Intern Med.

[63] Tellalian D, Maznavi K, Bredeek UF, Hardy WD (2013). Pre-exposure prophylaxis (PrEP) for HIV infection: results of a survey of HIV healthcare providers evaluating their knowledge, attitudes, and prescribing practices. AIDS Patient Care STDs.

[64] Bien C, Patel V, Blackstock O, Felsen U (2017). Reaching key populations: PrEP Uptake in an Urban health care system in the Bronx New York. AIDS Behav.

[65] Arnold EA, Hazelton P, Lane T (2012). A qualitative study of provider thoughts on implementing pre-exposure prophylaxis (PrEP) in clinical settings to prevent HIV infection. PLoS ONE.

[66] Desrosiers A, Levy M, Dright A (2018). A randomized controlled pilot study of a culturally-tailored counseling intervention to increase uptake of HIV pre-exposure prophylaxis among young black men who have sex with men in Washington DC. AIDS Behav.

[67] Doblecki-Lewis S, Jones D (2016). Community federally qualified health centers as homes for HIV preexposure prophylaxis: perspectives from South Florida. J Int Assoc Provid AIDS Care.

[68] Calabrese SK, Magnus M, Mayer KH (2016). Putting PrEP into practice: lessons learned from early-adopting U.S. providers' firsthand experiences providing HIV pre-exposure prophylaxis and associated care. PLoS ONE.

[69] Clement ME, Okeke NL, Munn T (2018). Partnerships between a university-affiliated clinic and community-based organizations to reach black men who have sex with men for PrEP care. J Acquir Immune Defic Syndr.

[70] Doblecki-Lewis S, Liu A, Feaster D (2017). Healthcare access and PrEP continuation in San Francisco and Miami after the US PrEP demo project. J Acquir Immune Defic Syndr.

[71] Ojikutu B, Bogart L, Mayer K (2019). Spatial access and willingness to use pre-exposure prophylaxis among black/African American individuals in the United States: Cross-Sectional Survey. JMIR Public Health Surveill.

[72] Paparini S, Nutland W, Rhodes T, Nguyen V, Anderson J (2018). DIY HIV prevention: formative qualitative research with men who have sex with men who source PrEP outside of clinical trials. PLoS ONE.

[73] Parisi D, Warren B, Leung SJ (2018). A multicomponent approach to evaluating a pre-exposure prophylaxis (PrEP) implementation program in five agencies in New York. J Assoc Nurs AIDS Care.

[74] Adams LM, Balderson BH (2016). HIV providers' likelihood to prescribe pre-exposure prophylaxis (PrEP) for HIV prevention differs by patient type: a short report. AIDS Care.

[75] Adams LM, Balderson BH, Brown K, Bush SE, Packett BJ (2018). Who starts the conversation and who receives preexposure prophylaxis (PrEP)? a brief online survey of medical providers’ PrEP practices. Health Educ Behav.

[76] Clement ME, Seidelman J, Wu J (2018). An educational initiative in response to identified PrEP prescribing needs among PCPs in the Southern U.S.. AIDS Care.

[77] Grimm J, Schwartz J (2018). "It's like birth control for HIV": communication and stigma for gay men on PrEP. J Homosex.

[78] Klassen BJ, Lin SY, Lachowsky NJ (2017). Gay men’s understanding and education of new HIV prevention technologies in vancouver Canada. Qual Health Res.

[79] Lelutiu-Weinberger C, Golub SA (2016). Enhancing PrEP access for black and latino men who have sex with men. J Acquir Immune Defic Syndr.

[80] Maloney KM, Krakower DS, Ziobro D (2017). Culturally competent sexual healthcare as a prerequisite for obtaining preexposure prophylaxis: findings from a qualitative study. LGBT Health.

[81] Raifman JRG, Flynn C, German D (2017). Healthcare provider contact and pre-exposure prophylaxis in baltimore men who have sex with men. Am J Prev Med.

[82] Hubach RD, Currin JM, Sanders CA (2017). Barriers to access and adoption of pre-exposure prophylaxis for the prevention of hiv among men who have sex with men (MSM) in a relatively rural state. AIDS Educ Prev.

[83] Beach LB, Greene GJ, Lindeman P (2018). Barriers and facilitators to seeking HIV services in Chicago among young men who have sex with men: perspectives of HIV service providers. AIDS Patient Care STDS.

[84] Aloysius I, Savage A, Zdravkov J (2017). (2017) InterPrEP. Internet-based pre-exposure prophylaxis with generic tenofovir DF/emtricitabine in London: an analysis of outcomes in 641 patients. J Virus Erad.

[85] Eaton LA, Matthews DD, Bukowski LA (2018). Elevated HIV prevalence and correlates of PrEP use among a community sample of black men who have sex with men. J Acquir Immune Defic Syndr.

[86] Levy ME, Watson CC, Glick SN (2016). Receipt of HIV prevention interventions is more common in community-based clinics than in primary care or acute care settings for Black men who have sex with men in the District of Columbia. AIDS Care.

[87] Mayer K, Safren S, Elsesser S (2017). Optimizing pre-exposure antiretroviral prophylaxis adherence in men who have sex with men: results of a pilot randomized controlled trial of 'life-steps for PrEP'. AIDS Behav.

[88] Elst E, Mbogua J, Operario D (2013). High acceptability of HIV pre-exposure prophylaxis but challenges in adherence and use: qualitative insights from a phase I trial of intermittent and daily PrEP in at-risk populations in Kenya. AIDS Behav.

[89] Fuchs JD, Stojanovski K, Vittinghoff E (2018). A mobile health strategy to support adherence to antiretroviral preexposure prophylaxis. AIDS Patient Care STDS.

[90] Landovitz RJ, Beymer M, Kofron R (2017). Plasma tenofovir levels to support adherence to TDF/FTC preexposure prophylaxis for HIV prevention in MSM in Los Angeles California. J Acquir Immune Defic Syndr.

[91] Pasipanodya EC, Jain S, Sun X (2018). Trajectories and predictors of longitudinal preexposure prophylaxis adherence among men who have sex with men. J Infect Dis.

[92] Gilmore HJ, Liu A, Koester KA (2013). Participant experiences and facilitators and barriers to pill use among men who have sex with men in the iPrEx pre-exposure prophylaxis trial in San Francisco. AIDS Patient Care STDs.

[93] Amico KR, McMahan V, Goicochea P (2012). Supporting study product use and accuracy in self-report in the iPrEx Study: next step counseling and neutral assessment. AIDS Behav.

[94] Witzel TC, Nutland W, Bourne A (2018). What qualities in a potential HIV pre-exposure prophylaxis service are valued by black men who have sex with men in London? A qualitative acceptability study. Int J STD AIDS.

[95] Galea JT, Kinsler JJ, Salazar X (2011). Acceptability of pre-exposure prophylaxis as an HIV prevention strategy: barriers and facilitators to pre-exposure prophylaxis uptake among at-risk Peruvian populations. Int J STD AIDS.

[96] Jaiswal J, Griffin M, Singer SN (2018). Structural barriers to pre-exposure prophylaxis use among young sexual minority men: the P18 Cohort Study. Curr HIV Res.

[97] Philbin MM, Parker CM, Parker RG (2018). Gendered social institutions and preventive healthcare seeking for black men who have sex with men: the promise of biomedical HIV prevention. Arch Sex Behav.

[98] Raifman J, Nunn A, Oldenburg CE (2018). An evaluation of a clinical pre-exposure prophylaxis education intervention among men who have sex with men. Health Serv Res.

[99] Bourne A, Alba B, Garner A (2019). Use of, and likelihood of using, HIV pre-exposure prophylaxis among men who have sex with men in Europe and Central Asia: findings from a 2017 large geosocial networking application survey. Sex Transm Infect.

[100] Grov C, Kumar N (2018). HIV pre-exposure prophylaxis (PrEP) is coming to Europe, but Are gay men ready to accept it? qualitative findings from Berlin Germany. Sex Res Soc Policy.

[101] Underhill K, Morrow KM, Colleran CM (2014). Access to healthcare, HIV/STI testing, and preferred pre-exposure prophylaxis providers among men who have sex with men and men who engage in street-based sex work in the US. PLoS ONE.

[102] Buttram ME (2018). The informal use of antiretroviral medications for HIV prevention by men who have sex with men in South Florida: initiation, use practices, medications and motivations. Cult Health Sex.

[103] Vaccher SJ, Kaldor JM, Callander D, Zablotska IB, Haire BG (2018). Qualitative insights into adherence to HIV pre-exposure prophylaxis (PrEP) among Australian gay and Bisexual men. AIDS Patient Care STDs.

[104] Karris MY, Beekmann SE, Mehta SR, Anderson CM, Polgreen PM (2014). Are we prepped for preexposure prophylaxis (PrEP)? Provider opinions on the real-world use of PrEP in the United States and Canada. Clin Infect Dis.

[105] Daughtridge GW, Conyngham SC, Ramirez N, Koenig HC (2015). I am men's health: generating adherence to HIV pre-exposure prophylaxis (PrEP) in young men of color who have sex with men. J Int Assoc Provid AIDS Care.

[106] Liu AY, Vittinghoff E, von Felten P (2018). Randomized controlled trial of a mobile health intervention to promote retention and adherence to pre-exposure prophylaxis among young people at risk for human immunodeficiency virus: the EPIC Study. Clin Infect Dis..

[107] Stekler JD, McMahan V, Ballinger L (2018). HIV pre-exposure prophylaxis prescribing through telehealth. J Acquir Immune Defic Syndr.

[108] Amico KR, Miller J, Balthazar C (2018). Integrated next step counseling (iNSC) for sexual health and PrEP use among young men who have sex with men: implementation and observations from ATN110/113. AIDS Behav.

[109] Rusie LK, Orengo C, Burrell D (2018). Preexposure prophylaxis initiation and retention in care over 5 years, 2012–2017: are quarterly visits too much?. Clin Infect Dis.

[110] Smith DK, Toledo L, Smith DJ, Adams MA, Rothenberg R (2012). Attitudes and program preferences of African-American Urban young adults about pre-exposure prophylaxis (PrEP). AIDS Educ Prev.

[111] Marcus JL, Hurley LB, Hare CB (2016). Preexposure prophylaxis for HIV prevention in a large integrated health care system: adherence, renal safety, and discontinuation. J Acquir Immune Defic Syndr.

[112] Mitchell JT, LeGrand S, Hightow-Weidman LB (2018). Smartphone-based contingency management intervention to improve pre-exposure prophylaxis adherence: pilot trial. JMIR Mhealth Uhealth.

[113] Dubov A, Fraenkel L, Yorick R, Ogunbajo A, Altice FL (2018). Strategies to implement pre-exposure prophylaxis with men who have sex with men in Ukraine. AIDS Behav.

[114] Galindo GR, Walker JJ, Hazelton P (2012). Community member perspectives from transgender women and men who have sex with men on pre-exposure prophylaxis as an HIV prevention strategy: implications for implementation. Implement Sci.

[115] Bhatia R, Modali L, Lowther M (2018). Outcomes of preexposure prophylaxis referrals from public STI clinics and implications for the preexposure prophylaxis continuum. Sex Transm Dis.

[116] Hojilla, J. C. Optimizing the delivery of HIV pre-exposure prophylaxis (PrEP): An evaluation of risk compensation, disengagement, and the PrEP cascade (part 2). 2018b. https://search.proquest.com/docview/1990873737?accountid=12118;https://ljmu-primo.hosted.exlibrisgroup.com/openurl/44JMU/44JMU_services_page??url_ver=Z39.88-2004&rft_val_fmt=info:ofi/fmt:kev:mtx:dissertation&genre=dissertations+%26+theses&sid=ProQ:PsycINFO+&atitle=&title=Optimizing+the+delivery+of+HIV+pre-exposure+prophylaxis+%28PrEP%29%3A+An+evaluation+of+risk+compensation%2C+disengagement%2C+and+the+PrEP+cascade&issn=&date=2018-01-01&volume=&issue=&spage=&au=Hojilla%2C+J.+Carlo&isbn=978-0355142525&jtitle=&btitle=&rft_id=info:eric/2017-54454-181&rft_id=info:doi/. Accessed 7 Jan 2019.

[117] Kwakwa HA, Bessias S, Sturgis D (2018). Engaging United States black communities in HIV pre-exposure prophylaxis: analysis of a PrEP engagement cascade. J Natl Med Assoc.

[118] Spinelli MA, Scott HM, Vittinghoff E (2018). Provider adherence to pre-exposure prophylaxis monitoring guidelines in a large primary care network. Open Forum Infect Dis.

[119] Siegler AJ, Bratcher A, Weiss KM (2018). Location location location: an exploration of disparities in access to publicly listed pre-exposure prophylaxis clinics in the United States. Ann Epidemiol.

[120] Newman PA, Guta A, Lacombe-Duncan A, Tepjan S (2018). Clinical exigencies, psychosocial realities: negotiating HIV pre-exposure prophylaxis beyond the cascade among gay, bisexual and other men who have sex with men in Canada. J Int AIDS Soc.

[121] Chan PA, Glynn TR, Oldenburg CE (2016). Implementation of preexposure prophylaxis for human immunodeficiency virus prevention among men who have sex with men at a New England Sexually transmitted diseases clinic. Sex Transm Dis.

[122] Arnold T, Brinkley-Rubinstein L, Chan PA (2017). Social, structural, behavioral and clinical factors influencing retention in Pre-Exposure Prophylaxis (PrEP) care in Mississippi. PLoS ONE.

[123] Hojilla, J. C. Optimizing the delivery of HIV pre-exposure prophylaxis (PrEP): An evaluation of risk compensation, disengagement, and the PrEP cascade (part 1). 2018a. https://search.proquest.com/docview/1990873737?accountid=12118;https://ljmu-primo.hosted.exlibrisgroup.com/openurl/44JMU/44JMU_services_page??url_ver=Z39.88-2004&rft_val_fmt=info:ofi/fmt:kev:mtx:dissertation&genre=dissertations+%26+theses&sid=ProQ:PsycINFO+&atitle=&title=Optimizing+the+delivery+of+HIV+pre-exposure+prophylaxis+%28PrEP%29%3A+An+evaluation+of+risk+compensation%2C+disengagement%2C+and+the+PrEP+cascade&issn=&date=2018-01-01&volume=&issue=&spage=&au=Hojilla%2C+J.+Carlo&isbn=978-0355142525&jtitle=&btitle=&rft_id=info:eric/2017-54454-181&rft_id=info:doi/. Accessed 7 Jan 2019

[124] Golub SA, Gamarel KE, Rendina HJ, Surace A, Lelutiu-Weinberger CL (2013). From efficacy to effectiveness: facilitators and barriers to PrEP acceptability and motivations for adherence among MSM and transgender women in New York City. AIDS Patient Care STDs.

[125] Kurtz SP, Buttram ME (2016). Misunderstanding of pre-exposure prophylaxis use among men who have sex with men: public health and policy implications. LGBT Health.

[126] Merchant RC, Corner D, Garza E (2016). Preferences for HIV pre-exposure prophylaxis (PrEP) information among men-who-have-sex-with-men (MSM) at community outreach settings. J Gay Lesbian Ment Health.

[127] Shover CL, Javanbakht M, Shoptaw S (2018). HIV preexposure prophylaxis initiation at a large community clinic: differences between eligibility, awareness, and uptake. Am J Public Health.

[128] Marcus JL, Levine K, Grasso C (2018). HIV preexposure prophylaxis as a gateway to primary care. Am J Public Health.

[129] Rivierez I, Quatremere G, Spire B, Ghosn J, Rojas Castro D (2018). Lessons learned from the experiences of informal PrEP users in France: results from the ANRS-PrEPage Study. AIDS Care.

[130] Refugio ON, Kimble MM, Silva CL (2018). PrEPTECH: a telehealth-based initiation program for human immunodeficiency virus pre-exposure prophylaxis in young men of color who have sex with men. A Pilot Study of feasibility. J Acquir Immune Defic Syndr.

[131] Ridgway JP, Almirol EA, Bender A (2018). Which patients in the emergency department should receive preexposure prophylaxis? Implementation of a predictive analytics approach. AIDS Patient Care STDs.

[132] Mutua G, Sanders E, Mugo P (2012). Safety and adherence to intermittent pre-exposure prophylaxis (PrEP) for HIV-1 in African men who have sex with men and female sex workers. PLoS ONE.

[133] Sullivan PS, Driggers R, Stekler JD (2017). Usability and acceptability of a mobile comprehensive HIV prevention app for men who have sex with men: a Pilot Study. JMIR Mhealth Uhealth.

[134] Young I, Flowers P, McDaid LM (2014). Barriers to uptake and use of pre-exposure prophylaxis (PrEP) among communities most affected by HIV in the UK: findings from a qualitative study in Scotland. BMJ Open.

[135] Nodin N, Carballo-Dieguez A, Ventuneac AM, Balan IC, Remien R (2008). Knowledge and acceptability of alternative HIV prevention bio-medical products among MSM who bareback. AIDS Care.

[136] Kojima N, Davey DJ, Klausner JD (2016). Pre-exposure prophylaxis for HIV infection and new sexually transmitted infections among men who have sex with men. AIDS.

[137] Holt M, Murphy DA, Callander D (2012). Willingness to use HIV pre-exposure prophylaxis and the likelihood of decreased condom use are both associated with unprotected anal intercourse and the perceived likelihood of becoming HIV positive among Australian gay and bisexual men. Sex Transm Infect.

[138] Holt M, Lea T, Murphy D (2014). Willingness to use HIV pre-exposure prophylaxis has declined among australian gay and bisexual men: results from repeated national surveys, 2011–2013. J Acquir Immune Defic Syndr.

[139] Hoff CC, Chakravarty D, Bircher AE (2015). Attitudes towards PrEP and anticipated condom use among concordant HIV-negative and HIV-discordant male couples. AIDS Patient Care STDs.

[140] Eaton LA, Kalichman S (2007). Risk compensation in HIV prevention: implications for vaccines, microbicides, and other biomedical HIV prevention technologies. Curr HIV/AIDS Rep.

[141] Cassell MM, Halperin DT, Shelton JD, Stanton D (2006). Risk compensation: the Achilles’ heel of innovations in HIV prevention?. BMJ.

[142] Bil JP, Davidovich U, van der Veldt WM (2015). What do Dutch MSM think of preexposure prophylaxis to prevent HIV-infection? A cross-sectional study. AIDS Behav.

[143] Eakle R, Venter F, Rees H (2018). Pre-exposure prophylaxis (PrEP) in an era of stalled HIV prevention: Can it change the game?. Retrovirology.

[144] Kippax S, Stephenson N (2012). Beyond the distinction between biomedical and social dimensions of HIV prevention through the lens of a social public health. Am J Public Health.

[145] Marks SJ, Merchant RC, Clark MA (2017). Potential healthcare insurance and provider barriers to pre-exposure prophylaxis utilization among young men who have sex with men. AIDS Patient Care STDs.

