# Supplementary material for: Pre-exposure Prophylaxis (PrEP) for HIV Prevention Among Men Who Have Sex with Men (MSM): A Scoping Review on PrEP Service Delivery and Programming
Source: AIDS Behav. 2020 Apr 9;24(11):3056–70. doi: 10.1007/s10461-020-02855-9 (PMC7502438; doi:10.1007/s10461-020-02855-9)
Supplement: Supplementary file 1 — Supplementary file1 (DOCX 60 kb) [file 10461_2020_2855_MOESM1_ESM.docx]

Supplementary Table S1 Supplemental Table of Included Studies: Issues, Themes and Findings

| **Authors** | **Article details** | **Key issues** | **Themes** | **Aims and findings** |
| --- | --- | --- | --- | --- |
| Adams, L. M. and B. H. Balderson | HIV providers' likelihood to prescribe pre-exposure prophylaxis (PrEP) for HIV prevention differs by patient type: a short report; AIDS Care; 2016; 28 (9); pp. 1154-1158 | • Prescribing habits and eligibility • Support services | • PrEP service aspects, settings and staff • PrEP prescriber experiences, therapeutic alliance and care planning | The article examined perceptions of frontline HIV care providers about PrEP and their likelihood of prescribing it to different patient groups. Findings suggest that providers' willingness to prescribe PrEP varies by patient group. There is a need for further research to determine barriers to receiving complete PrEP endorsement, along with exploration into factors that may prevent providers from prescribing PrEP to heterosexuals and IDUs. |
| Adams, L. M., B. H. Balderson, K. Brown, S. E. Bush and B. J. Packett | Who Starts the Conversation and Who Receives Preexposure Prophylaxis (PrEP)? A Brief Online Survey of Medical Providers’ PrEP Practices; Health Education & Behavior; 2018; 54 (5); 723-729 | • Prescribing habits and eligibility • Relationship with patient • Staffing and service allocation | • PrEP service aspects, settings and staff | The article discusses how PrEP uptake continues to accelerate and will likely provide evolving opportunities for wide-scale HIV prevention. Future work should continue to explore attitudes about and barriers to PrEP among providers and patients alike. While outreach efforts to raise general awareness about PrEP should be ongoing, PrEP is still unknown to many people. It is therefore necessary for providers to start the conversation to ensure that people who may benefit from PrEP know about it and can partake in it. Extending equitable knowledge and access to PrEP across groups at risk for HIV throughout the US should remain a continued goal for HIV prevention. |
| Aloysius, I., A. Savage, J. Zdravkov, R. Korologou-Linden, A. Hill, R. Smith, V. Houghton-Price, M. Boffito and N. Nwokolo | InterPrEP. Internet-based pre-exposure prophylaxis with generic tenofovir DF/emtricitabine in London: an analysis of outcomes in 641 patients; Journal of Virus Eradication; 2017; 3 (4); pp. 218-222 | • Staffing and service allocation | • PrEP service aspects, settings and staff | The article evaluated the service of informal PrEP use at 56 Dean Street, London. In 336 person-years of follow-up, there were no new cases of HIV and no serious adverse events. The recreational drug use reported by those taking generic PrEP decreased during PrEP use. However, there was a 10% increase in individuals diagnosed with an STI during PrEP follow-up, compared to those diagnosed in the 3 months before starting PrEP. The price of branded PrEP from Gilead is prohibitively high for most at-risk individuals. Online generic PrEP provides an opportunity for patients to access HIV prevention more affordably. A safe network of online suppliers and appropriate monitoring will enable individuals at risk of HIV infection to access an effective means of prevention as an interim solution until PrEP is available on the NHS to all who need it. Similar methods of access to generics could be established in other countries where branded PrEP is not available. |
| Amico, K. R., V. McMahan, P. Goicochea, L. Vargas, J. L. Marcus, R. M. Grant and A. Liu | Supporting study product use and accuracy in self-report in the iPrEx study: next step counseling and neutral assessment; AIDS & Behavior; 2012; 16 (5); pp. 1243-1259 | • Health communication and education • Service structure or model • Social media, apps and technology | • PrEP service aspects, settings and staff • PrEP adherence within formal service structures • Multi-disciplinary and innovative PrEP care pathways | The study synthesized all available data concerning current adherence support approaches and messaging at the study sites if the iPrEx Adherence Working Group (AWG). The authors evaluated the feasibility and acceptability of NSC and NA, which provides adherence support. NSC was well recieved, while only certain aspects of NA were accepted. In terms of factors facilitating the integration of PrEP into daily life, the most commonly recorded facilitator was incorporation into one’s existing daily routine, which could include linking tablet taking to a meal, waking in the morning, or some other regularly occurring event. The most frequently reported barrier was therefore disruption in routine. Some common strategies used at sites for promoting adherence was to work out specific dose times. |
| Amico, K. R., J. Miller, C. Balthazar, P. A. Serrano, J. Brothers, S. Zollweg and S. Hosek | Integrated Next Step Counseling (iNSC) for Sexual Health and PrEP Use Among Young Men Who Have Sex with Men: Implementation and Observations from ATN110/113; AIDS and Behavior; 2018 | • Service structure or model • Support services • Tailoring | • PrEP service aspects, settings and staff • PrEP adherence within formal service structures • Multi-disciplinary and innovative PrEP care pathways | The study assessed the success of iNSC among YMSM. Completed case report forms by iNSC counselors at study visits were used for data collection. Sessions noted 'commitment to staying negative' as a motivator for taking PrEP. The most common challenge was the assumption that partners were also negative. The most common need was greater access to sexual health clinics and services. Facilitators for adherence included carrying doses, which was challenged by alcohol and drug use. |
| Anand, T., C. Nitpolprasert, D. Trachunthong, S. J. Kerr, S. Janyam, D. Linjongrat, L. B. Hightow-Weidman, P. Phanuphak, J. Ananworanich and N. Phanuphak | A novel Online-to-Offline (O2O) model for pre-exposure prophylaxis and HIV testing scale up; Journal of the International AIDS Society; 2017; 20 (1); p. 21326 | • Service structure or model • Social media, apps and technology • Staffing and service allocation • Support services • Tailoring | • PrEP service aspects, settings and staff • Multi-disciplinary and innovative PrEP care pathways | The article evaluated the effect of a novel Adam’s Love Online-to-Offline (O2O) model on PrEP and HIV testing uptake among Thai MSM and TG and identifies factors associated with PrEP uptake. The authors found that greater attention should be placed on increasing PrEP awareness through public health campaigns targeting MSM with high-risk behaviours. Extending online promotions and outreach through existing social media networks of community-based sites staff and popular online platforms used for seeking sex are critical to reach, engage and scale up PrEP among high-risk groups in the future. Results demonstrate that PrEP uptake was unrelated with risk behaviours including drug use, condom-use behaviour, number of sexual partners and STI history, in contrast to previously published studies that have found PrEP acceptability to be correlated with higher-risk behaviours. More efforts are therefore needed to build self-risk assessment abilities and encourage PrEP uptake among these vulnerable MSM and TG groups, a significant consideration for the next generation of Adam’s Love O2O platform. Further, the O2O model was less successful at engaging MSM and TG with lower education to PrEP services, indicating future tailoring of the model for wider PrEP scale up. The study demonstrates that Adam’s Love O2O model is highly effective in linking online at-risk MSM and TG to PrEP and HIV testing services, using eCounseling and booking as ‘bridging steps’ likely by overcoming the barriers and challenges to PrEP uptake. |
| Arnold, E. A., P. Hazelton, T. Lane, K. A. Christopoulos, G. R. Galindo, W. T. Steward and S. F. Morin | A qualitative study of provider thoughts on implementing pre-exposure prophylaxis (PrEP) in clinical settings to prevent HIV infection; 2012; Plos One; 7 (7); e40603 | • Health communication and education • Prescribing habits and eligibility • Staffing and service allocation • Support services • Tailoring | • PrEP service aspects, settings and staff • Multi-disciplinary and innovative PrEP care pathways | The authors explored how medical and service providers understand research results and plan to develop clinical protocols to prescribe, support and monitor adherence for patients on PrEP. Topics discussed with healthcare providers included assessing clinician impressions of PrEP and CDC guidance, considerations of cost, office capacity, dosing schedules, and following patients over time. The study found that there was minimal demand for PrEP at the time the interviews were conducted. There were disputes as to the eligibility of patients for PrEP, stating that current models of care, which do not involve routine frequent office visits, were not well suited for prescribing PrEP. Providers detailed the need to build capacity and were concerned about monitoring side effects and adherence. They also noted that community education campaigns needed to be tailored to effectively reach specific vulnerable populations. |
| Arnold, T., L. Brinkley-Rubinstein, P. A. Chan, A. Perez-Brumer, E. S. Bologna, L. Beauchamps, K. Johnson, L. Mena and A. Nunn | Social, structural, behavioral and clinical factors influencing retention in Pre-Exposure Prophylaxis (PrEP) care in Mississippi; Plos One; 2017; 12 (2); p. e0172354; | • Health communication and education | • Multi-disciplinary and innovative PrEP care pathways | The article assessed structural, social, behavioral, and clinical factors that may have affected individuals’ uptake, adherence, and retention in PrEP care. The authors' findings suggest that structural factors such as insurance, costs and co-payments as well as social factors such as relationship dynamics and stigma impacted PrEP uptake and retention in PrEP care. Behavioral factors including sexual risk behaviors and clinical factors such as actual and perceived side effects also affected participant’s decisions about starting and continuing to take PrEP. Additionally, participants reported many unintentional positive, health-related spill-over effects of taking PrEP and many of the MSM who had discontinued PrEP use re-initiated after participating in the study. Addressing structural factors such as cost or access to payment assistance programs, social factors such as stigma and relationship dynamics, and clinical and behavioral factors such as anticipated or experienced side effects of PrEP medication and sexual risk behaviors is imperative. Outreach enabled re-engagement of several patients in PrEP care who had previously been lost to follow-up. Future research to promote PrEP uptake and retention in care should address social, structural, behavioral and clinical factors. |
| Beach, L. B., G. J. Greene, P. Lindeman, A. K. Johnson, C. N. Adames, M. Thomann, P. C. T. Washington and G. Phillips Ii | Barriers and Facilitators to Seeking HIV Services in Chicago Among Young Men Who Have Sex with Men: Perspectives of HIV Service Providers; AIDS Patient Care & STDS; 2018; 32 (11); pp. 468-476 | • Health communication and education • Peer and social networks • Relationship with patient • Service structure or model • Staffing and service allocation • Tailoring | • PrEP service aspects, settings and staff • PrEP prescriber experiences, therapeutic alliance and care planning • Multi-disciplinary and innovative PrEP care pathways | The article assesses how providers perceive these facilitators and barriers to HIV services. Results of the study highlight potential opportunities for interventions to address barriers related to health system characteristics on the microsystem level (i.e., programming designed to increase individual understanding about lack of invincibility and building their capacity and knowledge for how to seek HIV services), the mesosystem level (i.e., providers working together to increase an individual’s trust of the medical system and ensuring confidentiality), and the macrosystem level (i.e., ensure comprehensive services are available to all individuals). The identification of community outreach and geography and transportation as barriers suggests that researchers and evaluators should consider incorporating community surveys and geospatial analysis into evaluations to better understand how the meso-, macro-, and exosystems influence engagement outcomes. Findings from these studies could then inform future efforts to address these barriers. Results indicate that providers report that complex, inter-related factors affect YMSM’s decision making for why YMSM choose to engage in HIV care. |
| Bhatia, R., L. Modali, M. Lowther, N. Glick, M. Bell, S. Rowan, K. Keglovitz and J. Schneider | Outcomes of Preexposure Prophylaxis Referrals From Public STI Clinics and Implications for the Preexposure Prophylaxis Continuum; Sexually Transmitted Diseases; 2018; 45 (1); pp. 50-55 | • Prescribing habits and eligibility • Referral • Service structure or model • Support services • Tailoring | • PrEP service aspects, settings and staff • Multi-disciplinary and innovative PrEP care pathways | The authors developed a PrEP active referral mechnism, for at risk patients to be signposted to PrEP community partner sites. The article therefore describes the outcomes of the implementation model, framed in the context of an adapated PrEP continuum. The data demonstrate that a PrEP active referral mechanism from public STI clinics to PrEP partner sites is feasible and reaches high-risk individuals. Further work addressing barriers to linkage, particularly among youth, is needed to optimize engagement along the PrEP continuum. |
| Bien, C., V. Patel, O. Blackstock and U. Felsen | Reaching Key Populations: PrEP Uptake in an Urban Health Care System in the Bronx, New York; AIDS & Behavior; 2017; 5; pp. 1309-1314 | • Prescribing habits and eligibility • Staffing and service allocation | • PrEP service aspects, settings and staff | The study used a clinic database and chart review to identify individuals prescribed PrEP. Findings showed a large increase in PrEP prescribing over time, but overall PrEP prescribing was low. PrEP prescriptions occurred across a range of clinical settings, and that most PrEP prescribing occurred in primary care centers and a single sexual health center. There is a need to identify effective strategies to increase PrEP prescription in both primary care and subspecialty settings and to better engage populations at high risk for HIV infection ahead of a PrEP scale up. |
| Bourne, A., B. Alba, A. Garner, G. Spiteri, A. Pharris and T. Noori | Use of, and likelihood of using, HIV pre-exposure prophylaxis among men who have sex with men in Europe and Central Asia: findings from a 2017 large geosocial networking application survey; Sexually Transmitted Infections; 2019 | • Health communication and education • Prescribing habits and eligibility • Relationship with patient • Support services | • PrEP prescriber experiences, therapeutic alliance and care planning | The study examined the current use of PrEP, likelihood of future use and indicators of potential PrEP candidacy among an opportunistic sample of men who have sex with men in Europe and central Asia. Further research is required that examines HIV/STI testing practices and other health monitoring of people accessing PrEP online. Findings indicate that the odds of engaging in chemsex, having received an STI diagnosis or previous use of PEP were all significantly higher among men using PrEP compared with those who were not. A large proportion of men are accessing PrEP outside of traditional healthcare settings, posing a challenge for routine monitoring. |
| Buttram, M. E. | The informal use of antiretroviral medications for HIV prevention by men who have sex with men in South Florida: initiation, use practices, medications and motivations; Culture, Health & Sexuality; 2018; 20 (11); pp. 1185-1198 | • Health communication and education • Relationship with patient • Social media, apps and technology | • PrEP prescriber experiences, therapeutic alliance and care planning • Multi-disciplinary and innovative PrEP care pathways | The authors examined the phenomena of gay and other men who have sex with men using antiretroviral medications informally, without a prescription. Participants described using a range of medications not approved for PrEP and combining several medications as an HIV prevention ‘cocktail’. Intermittent or sporadic use of antiretroviral medication, inconsistent access to medication and the use of medications not approved for PrEP may potentially leave men with less protection against HIV infection, and contribute to HIV transmission, resistance or adverse effects including drug toxicity, drug interactions and hypersensitivity reactions. Efforts should be made to enhance access to this HIV prevention technology. As more individuals start using PrEP, informal antiretroviral medication use and related concerns – including adherence, diversion and antiretroviral medication resistance – must be considered. Enthusiasm for biomedical HIV interventions among gay and other MSM appears to be high. Building on this enthusiasm, efforts should be made by researchers, public health officials and community and social services agencies to increase PrEP awareness and acceptability and decrease informal use and diversion. |
| Calabrese, S. K., M. Magnus, K. H. Mayer, D. S. Krakower, A. I. Eldahan, L. A. Gaston Hawkins, N. B. Hansen, T. S. Kershaw, K. Underhill, J. R. Betancourt and J. F. Dovidio | Putting PrEP into practice: Lessons learned from early-adopting U.S. providers' firsthand experiences providing HIV pre-exposure prophylaxis and associated care; Plos One; 2016; 11 (6) | • Health communication and education • Level of knowledge • Prescribing habits and eligibility • Relationship with patient • Service structure or model • Staffing and service allocation • Support services • Tailoring | • PrEP service aspects, settings and staff • PrEP prescriber experiences, therapeutic alliance and care planning • Multi-disciplinary and innovative PrEP care pathways | The study explored PrEP providers’ first hand experiences relative to commonly-cited barriers. Nearly all providers indicated that they were self-educated with respect to PrEP. Rather than participating in formal training, their knowledge about PrEP was obtained through a combination of reading relevant literature, attending professional talks and conferences, consulting with colleagues, and treating HIV-positive patients with the same antiretroviral medication. Providers described their experience of initiating and monitoring patients on PrEP favorably and were keen to continue prescribing PrEP. They described various models of implementation within and between health centers, including collaboration between infectious disease specialists and primary care providers. |
| Chan, P. A., T. R. Glynn, C. E. Oldenburg, M. C. Montgomery, A. E. Robinette, A. Almonte, J. Raifman, L. Mena, R. Patel, K. H. Mayer, L. S. Beauchamps and A. S. Nunn | Implementation of Preexposure Prophylaxis for Human Immunodeficiency Virus Prevention Among Men Who Have Sex With Men at a New England Sexually Transmitted Diseases Clinic; Sexually Transmitted Diseases; 2016; 43 (11); pp. 717-723 | • Health communication and education • Peer and social networks • Support services | • Multi-disciplinary and innovative PrEP care pathways | The study assessed PrEP uptake among MSM presenting for services at a STD clinic. To promote PrEP among MSM, PrEP education and counseling were integrated into standard care at our STD clinic. We found that acceptability of PrEP education was high among MSM, as most patients were amenable to receiving PrEP education. Nonetheless, the overall rate of PrEP uptake was low, similar to other reports in STD clinic settings. PrEP uptake was lower among racial and ethnic minorities, likely reflecting a combination of individual- and structural-level factors including insurance status. The most common reason individuals were not interested in PrEP was low perceived HIV risk. Results of this study highlight barriers to uptake and opportunities for enhancing STD clinic-based PrEP programs. Interventions that improve understanding of HIV risk perception may enhance uptake. In addition, future studies should also evaluate PrEP retention in care and adherence outside of research settings. From a programmatic perspective, low PrEP uptake in our clinic highlights opportunities to improvePrEP education. |
| Clement, M. E., N. L. Okeke, T. Munn, M. Hunter, K. Alexis, A. Corneli, A. C. Seña, K. McGee and M. S. McKellar | Partnerships Between a University-Affiliated Clinic and Community-Based Organizations to Reach Black Men Who Have Sex With Men for PrEP Care; Journal of Acquired Immune Deficiency Syndromes; 2018; 77 (2); e25-e27 | • Health communication and education • Level of knowledge • Prescribing habits and eligibility • Referral • Staffing and service allocation • Support services | • PrEP service aspects, settings and staff • PrEP prescriber experiences, therapeutic alliance and care planning • Multi-disciplinary and innovative PrEP care pathways | The authors sought to demonstrate the value of community partnerships to reach those at greatest risk for HIV infection. They found that the most common referral source for Black and Black MSM patients was through CBCs, demonstrating that community partnerships can be a valuable avenue for patient recruitment. While large-scale efforts are needed to improve PrEP awareness and linkage to care, CBCs can play a critical role in reaching populations at high risk with messages about PrEP. Further research and programmatic support should be granted to CBCs to help them reach the population most affected by HIV. |
| Clement, M. E., J. Seidelman, J. Wu, K. Alexis, K. McGee, N. L. Okeke, G. Samsa and M. S. McKellar | An educational initiative in response to identified PrEP prescribing needs among PCPs in the Southern U.S; AIDS Care; 2018; 30 (5); pp. 650-655 | • Health communication and education • Prescribing habits and eligibility • Staffing and service allocation • Support services | • PrEP service aspects, settings and staff • PrEP prescriber experiences, therapeutic alliance and care planning | The study examined existing knowledge about PrEP prescribing patterns among PCPs. It identified lack of knowledge about PrEP as the largest barrier to prescribing. Almost all PCPs were willing to prescribe PrEP, and most felt that additional training would encourage them to start prescribing. Using an educational campaign to train PCPs, the authors found that the rate of those prescribing PrEP doubled between the initial and repeated survey 11 months later. Among PCPs who underwent an on-site training, the rate of those prescribing was greater than 50%. |
| Daughtridge, G. W., S. C. Conyngham, N. Ramirez and H. C. Koenig | I Am Men's Health: Generating Adherence to HIV Pre-Exposure Prophylaxis (PrEP) in Young Men of Color Who Have Sex with Men; Journal of the International Association of Providers of AIDS Care; 2015; 14 (2); pp. 103-107 | • Peer and social networks • Social media, apps and technology • Staffing and service allocation • Support services | • PrEP service aspects, settings and staff • PrEP adherence within formal service structures • Multi-disciplinary and innovative PrEP care pathways | The study aimed to generate adherence to PrEP among high-risk YMSM in a community setting by counducting the Youth Health Empowement Project. The Project was a PrEP program targeting young Black MSM at high risk of HIV. Following participation in the program, adherence to PrEP was excellent, with a weighted average of 73%. This adherence was sustained over time with 88% still maintaining their regimen after 28 weeks. |
| Desrosiers, A., M. Levy, A. Dright, M. Zumer, N. Jallah, I. Kuo, M. Magnus and M. Siegel | A Randomized Controlled Pilot Study of a Culturally-Tailored Counseling Intervention to Increase Uptake of HIV Pre-exposure Prophylaxis Among Young Black Men Who Have Sex with Men in Washington, DC; AIDS and Behavior; 2018; 23; pp. 105-115 | • Referral • Social media, apps and technology • Staffing and service allocation • Support services | • PrEP service aspects, settings and staff • Multi-disciplinary and innovative PrEP care pathways | To explore whether a culturally-tailored counseling center for young Black MSM positively impacted access and uptake of PrEP. The study found that when PrEP counseling is performed in a culturally competent setting by a culturally sensitive counselor, this can further improve PrEP uptake in young BMSM. |
| Doblecki-Lewis, S. and D. Jones | Community Federally Qualified Health Centers as Homes for HIV Preexposure Prophylaxis: Perspectives from South Florida; Journal of the International Association of Providers of AIDS Care; 2016; 15 (6); pp. 522-528 | • Health communication and education • Prescribing habits and eligibility • Relationship with patient • Social media, apps and technology • Staffing and service allocation • Support services | • PrEP service aspects, settings and staff • PrEP prescriber experiences, therapeutic alliance and care planning • Multi-disciplinary and innovative PrEP care pathways | The article explored the feasibility, acceptability, and uptake of PrEP among healthcare staff.During the focus group participants generally felt PrEP implementation was feasible at their sites, although demand for PrEP was reported to be low and several barriers to implementation were identified including stigma, concern about risk compensation, and difficulty of obtaining accurate risk assessment to identify PrEP candidates. |
| Doblecki-Lewis, S., A. Liu, D. Feaster, S. E. Cohen, G. Cardenas, O. Bacon, E. Andrew and M. A. Kolber | Healthcare Access and PrEP Continuation in San Francisco and Miami After the US PrEP Demo Project; Journal of Acquired Immune Deficiency Syndromes; 2017; 74 (5); pp. 531-538 | • Level of knowledge • Prescribing habits and eligibility • Relationship with patient • Service structure or model • Staffing and service allocation • Support services | • PrEP service aspects, settings and staff | The study reports the findings of a follow up survey from a PrEP demonstration project, administered 4-6 months after study completion. PrEP interest remained high among a large majority of respondents and did not significantly differ by geographic location, age, race, ethnicity, income, or insurance status. PrEP access following study completion, however, was variable. Many participants, particularly white participants and those from the San Francisco site, were able to successfully transition into PrEP care outside of the study, although one-third did encounter a gap between study completion and initiation of PrEP from another source. For the majority, PrEP was obtained through PCPs, and those who successfully obtained PrEP generally felt that the process was “easy” or “very easy,” suggesting that once a PCP was accessed and a plan for PrEP was initiated, significant barriers were not commonly encountered. The most significant bottleneck to continued PrEP engagement occurred with access to a medical provider were geographic and socioeconomic disparities in access to PrEP. Addressing these barriers will be required to achieve levels of PrEP engagement sufficient for a meaningful public health impact and to ensure distribution of PrEP access that includes communities most at risk of HIV. |
| Dubov, A., L. Fraenkel, R. Yorick, A. Ogunbajo and F. L. Altice | Strategies to Implement Pre-exposure Prophylaxis with Men Who Have Sex with Men in Ukraine; AIDS & Behavior; 2018; 4; pp. 110-1112 | • Service structure or model • Staffing and service allocation | • PrEP service aspects, settings and staff • PrEP adherence within formal service structures | The study looked to understand Ukrainian MSM preferences in order to inform program development and facilitation of the successful delivery of PrEP. The findings indicate that PrEP uptake is most likely to be successful when PrEP is affordable, its implementation is targeted and provided as “on demand” with associated education, and when more thorough medical care and related testing is provided to at-risk (and traditionally marginalized) populations. |
| Eaton, L. A., D. D. Matthews, L. A. Bukowski, M. R. Friedman, C. J. Chandler, D. L. Whitfield, J. M. Sang and R. D. Stall | Elevated HIV Prevalence and Correlates of PrEP Use Among a Community Sample of Black Men who Have Sex with Men; Journal of Acquired Immune Deficiency Syndromes; 2018; 79; pp. 339-346 | • Prescribing habits and eligibility • Service structure or model • Staffing and service allocation | • PrEP service aspects, settings and staff • PrEP adherence within formal service structures | The article assessed and evaluated variables associated with PrEP use in a large, community-based sample of Black MSM. Findings show that around 1 of 3 Black MSM are unaware of the availability of PrEP, and only 10-20% are accessing PrEP. It is evident that to optimize PrEP, comprehensive strategies to following patients prescribed PrEP are needed. Trials of PrEP efficacy and delivery typically include well-resourced approaches to patient engagement including high levels of patient monitoring. Implementing a comprehensive plan for providing PrEP (eg, quarterly check-ins, adherence support, and sexual risk reduction counseling) poses greater challenges. |
| Elst, E., J. Mbogua, D. Operario, G. Mutua, C. Kuo, P. Mugo, J. Kanungi, S. Singh, J. Haberer, F. Priddy and E. Sanders | High Acceptability of HIV Pre-exposure Prophylaxis but Challenges in Adherence and Use: Qualitative Insights from a Phase I Trial of Intermittent and Daily PrEP in At-Risk Populations in Kenya; AIDS & Behavior; 2013; 6; pp. 2162-2172 | • Health communication and education • Service structure or model • Support services | • PrEP service aspects, settings and staff • PrEP adherence within formal service structures • Multi-disciplinary and innovative PrEP care pathways | The study explored experiences of MSM and FSW who use PrEP. Whilst the findings show that acceptability of PrEP is high, they highlight a number of factors that might impede adherence and potential scale-up. These include the social impacts such as stigma and the complexities of adherence. PrEP interventions must consider the synergies between the drug, the individual user’s behavior, the societal context, adherence, and social harms as a result of taking PrEP outside the research setting. Behavioral and social interventions that address the contextual realities of PrEP users will be fundamental in building effective and sustainable programs and policies for wide PrEP implementation. |
| Fuchs, J. D., K. Stojanovski, E. Vittinghoff, V. M. McMahan, S. G. Hosek, K. R. Amico, A. Kouyate, H. J. Gilmore, S. P. Buchbinder, R. T. Lester, R. M. Grant and A. Y. Liu | A Mobile Health Strategy to Support Adherence to Antiretroviral Preexposure Prophylaxis; AIDS Patient Care & STDS; 2018; 32 (3); pp. 104-111 | • Social media, apps and technology | • PrEP service aspects, settings and staff • PrEP adherence within formal service structures • Multi-disciplinary and innovative PrEP care pathways | The study evaluated a mobile health intervention (iText) that utilized weekly bidirectional text or e-mail support messages to encourage pre-exposure prophylaxis (PrEP) adherence among participants in the multi-site iPrEx open-label extension study. The authors found iText to be feasible and acceptable, particularly among younger participants and participants of color. A majority preferred receiving messages by SMS over e-mail and while some participants chose receiving generic messages that inquired whether they were okay or not, most selected PrEP-specific language in those messages. |
| Galea, J. T., J. J. Kinsler, X. Salazar, S. J. Lee, M. Giron, J. N. Sayles, C. Cáceres, W. E. Cunningham, J. T. Galea, J. J. Kinsler, X. Salazar, S. J. Lee, M. Giron, J. N. Sayles, C. Cáceres and W. E. Cunningham | Acceptability of pre-exposure prophylaxis as an HIV prevention strategy: barriers and facilitators to pre-exposure prophylaxis uptake among at-risk Peruvian populations; International Journal of STD & AIDS; 2011; 22 (5); pp. 256-262 | • Relationship with patient • Staffing and service allocation | • PrEP prescriber experiences, therapeutic alliance and care planning | The authors found a wide range of attitudes and opinions regarding PrEP acceptability. Important potential barriers to PrEP included high out-of-pocket cost, partial efficacy and fear of side-effects. Stigma and discrimination associated with PrEP use, mistrust of health-care professionals and a belief that PrEP would result in a decrease in condom use were concerns for MSM and TG. |
| Galindo, G. R., J. J. Walker, P. Hazelton, T. Lane, W. T. Steward, S. F. Morin and E. A. Arnold | Community member perspectives from transgender women and men who have sex with men on pre-exposure prophylaxis as an HIV prevention strategy: implications for implementation; Implementation Science; 2012; 7 (1); p. 116 | • Health communication and education • Service structure or model • Tailoring | • PrEP service aspects, settings and staff • Multi-disciplinary and innovative PrEP care pathways | The study explored social and cultural influences that may play a role in their decision to use or not to use PrEP as an HIV prevention strategy. Findings identify that the communities most impacted by the HIV epidemic are knowledgeable about PrEP; that questions and concerns regarding medical mistrust and side effects are addressed; that PrEP is accessible financially; and that PrEP is a complete intervention package inclusive of education, condom promotion and linkage to care. Even with clinically proven individual-level efficacy, if not packaged, implemented, and sustained properly, PrEP could increase HIV health disparities at the population level that it initially had the potential to eliminate. |
| Gilmore, H. J., A. Liu, K. A. Koester, K. R. Amico, V. McMahan, P. Goicochea, L. Vargas, D. Lubensky, S. Buchbinder and R. Grant | Participant Experiences and Facilitators and Barriers to Pill Use Among Men Who Have Sex with Men in the iPrEx Pre-Exposure Prophylaxis Trial in San Francisco; AIDS Patient Care & STDs; 2013; 27 (10); pp. 560-566 | • Health communication and education • Relationship with patient • Social media, apps and technology • Support services | • PrEP service aspects, settings and staff • PrEP prescriber experiences, therapeutic alliance and care planning • PrEP adherence within formal service structures • Multi-disciplinary and innovative PrEP care pathways | The study sought to understand individual and contextual factors influencing study product use in this community. Facilitators to pill taking included having clear motivation to take the pill to help answer an important scientific question; accurate information about the study pill; skills for pill-taking, either antecedent to or developed while in the study, including establishing a routine; and strong positive relationships with the study team and engaging in the counseling they offered. Barriers included changes in routine; side effects or intercurrent illnesses; stress; and rarely, stigma. Adherence has been described as the ‘‘Achilles heel’’ of PrEP; optimizing adherence will be critical to maximizing the public health impact of PrEP implementation. |
| Golub, S. A., K. E. Gamarel, H. J. Rendina, A. Surace and C. L. Lelutiu-Weinberger | From Efficacy to Effectiveness: Facilitators and Barriers to PrEP Acceptability and Motivations for Adherence Among MSM and Transgender Women in New York City; AIDS Patient Care & STDs; 2013; 27 (4); pp. 248-254 | • Health communication and education • Service structure or model • Staffing and service allocation • Support services | • PrEP service aspects, settings and staff • Multi-disciplinary and innovative PrEP care pathways | The study examined potential facilitators and barriers to pre-exposure prophylaxis (PrEP) use and their association with PrEP acceptability and motivations for adherence. Overall, 55.4% of participants reported willingness to take PrEP. The most highly endorsed barriers to PrEP use were health concerns, including both long-term impacts and short-term side effects, questions about PrEP’s impact on future drug resistance, and concerns that PrEP does not provide complete protection against HIV. The most highly endorsed facilitator was free access to PrEP, followed by access to support services such as regular HIV testing, sexual health care/monitoring, and access to one-on-one counseling. Participants of color rated both barriers and facilitators as more important than their White counterparts. The findings from this study underscore the importance of implementing clinical guidelines for the provision of PrEP, which would include monitoring side effects and adherence, as well as the provisions of ongoing HIV testing and sexual health counseling. |
| Grimm, J. and J. Schwartz | It's Like Birth Control for HIV: Communication and Stigma for Gay Men on PrEP; Journal of Homosexuality; 2018; pp. 2-20 | • Health communication and education • Level of knowledge • Peer and social networks • Relationship with patient • Social media, apps and technology | • PrEP service aspects, settings and staff • PrEP prescriber experiences, therapeutic alliance and care planning • Multi-disciplinary and innovative PrEP care pathways | The article investigates the experiences of gay men who have adopted PrEP. The findings demonstrate that stigma plays a substantial role in participants’ experiences with PrEP. Men are learning of PrEP through news media outlets—instead relying heavily on interpersonal networks. Furthermore, they identified that healthcare providers need training to allow them to understand and effectively communicate with their MSM patients. Findings also suggest that some healthcare providers have limited knowledge of PrEP. A campaign aimed at mitigating that stigma could be especially beneficial. The authors recommend that health care providers should be trained in minimizing the expression of stigmatizing attitudes and should increase their knowledge of PrEP. |
| Grov, C. and N. Kumar | HIV Pre-Exposure Prophylaxis (PrEP) Is Coming to Europe, but Are Gay Men Ready to Accept It? Qualitative Findings from Berlin, Germany; Sexuality Research and Social Policy; 2018; 15 (3); pp. 283-289 | • Relationship with patient • Staffing and service allocation | • PrEP prescriber experiences, therapeutic alliance and care planning | The article discussed how the rollout of PrEP is a complex process involving a range of factors including educating those who would benefit from about it, educating providers about how to talk about PrEP with their patients, sustaining affordable methods for patients to obtain PrEP, achieving optimal adherence for those on PrEP, and maintaining retention in the health care system such that PrEP recipients (people who may be otherwise healthy) return for quarterly medical visits to remain on PrEP. The authors found that there was high interest in PrEP among men in the study and that some are finding ways to obtain it without governmental approval. |
| Hoffman, S., J. A. Guidry, K. L. Collier, J. E. Mantell, D. Boccher-Lattimore, F. Kaighobadi and T. G. M. Sandfort | A Clinical Home for Preexposure Prophylaxis: Diverse Health Care Providers' Perspectives on the "Purview Paradox"; Journal of the International Association of Providers of AIDS Care; 2016; 15 (1); pp. 59-65 | • Level of knowledge • Purview Paradox • Relationship with patient • Staffing and service allocation • Support services | • PrEP service aspects, settings and staff • PrEP prescriber experiences, therapeutic alliance and care planning • Multi-disciplinary and innovative PrEP care pathways | The study explored issues related to PrEP roll-out, including who should provide it and in what settings among clinicans. The providers identified specific skills they thought necessary for those who would prescribe PrEP. To obtain these skills, training and education will be required as well as as sexual history-taking, which is arguably important for individual and public health, beyond HIV risk. Ultimately, a public health approach to HIV prevention and sexual health would view PrEP as one of many tools that should be available to people who are at high risk for HIV infection, wherever they are seeking medical care. |
| Hojilla, J. C. | Optimizing the delivery of HIV pre-exposure prophylaxis (PrEP): An evaluation of risk compensation, disengagement, and the PrEP cascade: HIV serodisclosure among MSM and transgender women on HIV PrEP; AAI10282719; 2018; pp. 11-32 | • Social media, apps and technology | • Multi-disciplinary and innovative PrEP care pathways | The study identified baseline factors associated with participant non-disclosure and lack of knowledge of partner status; risk factors of PrEP disengagement among MSM; and prognostic indicators for discontinuation in care among MSM enrolled in a clinic-based cohort. The findings suggest a higher prevalence of non-disclosure and lack of knowledge of partner status among PrEP users. Substantive differences in the prevalence of non-disclosure and lack of knowledge of partner status across study regions was also observed. Prevalence for both were particularly high in Thailand, the Andes, and Brazil. Differences may reflect a complex array and interaction between structural barriers, such as the availability of testing services, and sociocultural factors, like attitudes towards disclosing or asking about sensitive topics particularly in the context of a sexual encounter, homophobia, and HIV stigma. Additionally, the availability of internet and phone application-based social networks for finding sexual partners may also have contributed to the variability across regions. Lastly, the significant associations between relationship characteristics and discussions about HIV status underscore the importance of how dyadic factors influence HIV prevention efforts. Risk reduction counseling provided in conjunction with PrEP should help patients develop feasible strategies to reduce their risk. |
| Hojilla, J. C. | Optimizing the delivery of HIV pre-exposure prophylaxis (PrEP): An evaluation of risk compensation, disengagement, and the PrEP cascade: Stimulant use is associated with PrEP disengagement in men who have sex with men and transgender women; AAI10282719; 2018; pp. 33-51 | • Prescribing habits and eligibility • Support services | • PrEP service aspects, settings and staff | The study identified baseline factors associated with participant non-disclosure and lack of knowledge of partner status; risk factors of PrEP disengagement among MSM; and prognostic indicators for discontinuation in care among MSM enrolled in a clinic-based cohort. The authors found strong evidence to suggest that stimulant use is associated with PrEP disengagement. An association between binge drinking and PrEP disengagement was also observed. The findings underscore the need for a comprehensive approach to prevention that combines PrEP with behavioral interventions to mitigate disengagement and optimize its public health benefit. |
| Hojilla, J. C., D. Vlahov, P. Crouch, C. Dawson-Rose, K. Freeborn and A. Carrico | HIV Pre-exposure Prophylaxis (PrEP) Uptake and Retention Among Men Who Have Sex with Men in a Community-Based Sexual Health Clinic; AIDS & Behavior; 2018; 4; pp. 1096-1099 | • Staffing and service allocation | • Multi-disciplinary and innovative PrEP care pathways | The article characterized key steps of the pre-exposure prophylaxis (PrEP) cascade and to identify correlates of retention in care. The authors observed a high uptake among individuals who sought PrEP services at the clinic, consistent with what others have reported previously. However, structural barriers to access, like cost, remain challenging for many patients. Despite the availability of PrEP navigators at the clinic who helped patients access insurance and drug assistance programs, a sizeable proportion (22%) did not initiate PrEP. |
| Hubach, R. D., J. M. Currin, C. A. Sanders, A. R. Durham, K. E. Kavanaugh, D. L. Wheeler and J. M. Croff | Barriers to Access and Adoption of Pre-Exposure Prophylaxis for the Prevention of HIV Among Men Who Have Sex With Men (MSM) in a Relatively Rural State; AIDS Education & Prevention; 2017; 29 (4); pp. 315-329 | • Health communication and education • Peer and social networks • Relationship with patient • Service structure or model • Social media, apps and technology • Staffing and service allocation • Tailoring | • PrEP service aspects, settings and staff • PrEP prescriber experiences, therapeutic alliance and care planning • Multi-disciplinary and innovative PrEP care pathways | The article discusses how despite the shown efficacy of PrEP in preventing HIV in at-risk populations, MSM residing in relatively rural states perceive substantial barriers to accessing PrEP. The social environment, specifically in regards to stigma from within the LGBT community and from medical providers, was frequently discussed as a prominent factor influencing PrEP access and adoption. MSM in Oklahoma perceive substantial barriers to adopting PrEP, which may be similar to other states with similar urban/rural proportions. Accessing quality, LGBT-sensitive care can help mitigate potential barriers; however, it still requires providers implementing PrEP within their practice. |
| Jaiswal, J., M. Griffin, S. N. Singer, R. E. Greene, I. L. Z. Acosta, S. K. Kaudeyr, F. Kapadia and P. N. Halkitis | Structural Barriers to Pre-exposure Prophylaxis Use Among Young Sexual Minority Men: The P18 Cohort Study; Current HIV Research; 2018; 16 (3); pp. 237-249 | • Health communication and education • Relationship with patient • Service structure or model • Support services | • PrEP service aspects, settings and staff • PrEP prescriber experiences, therapeutic alliance and care planning • PrEP adherence within formal service structures • Multi-disciplinary and innovative PrEP care pathways | The article identifies and discusses the individual-level factors of the slow uptake of PrEP. This extends to structural drivers and wider HIV prevention strategies. While almost all participants indicated awareness of PrEP, only 14% had ever used PrEP. PrEP use was associated with lower concerns about health care access, particularly paying for PrEP. Those with greater concerns talking with their provider about their sexual behaviors were less likely to use PrEP. Prior to the introduction of PrEP, the message from health care providers regarding sexual risk among MSM predominantly revolved around overcoming barriers to condom use, and were often fear-based. It is critical for providers who prescribe PrEP to initiate conversations about sexual risk in an open, non-judgmental way with all patients who may benefit from PrEP. This will demand that many providers reframe previous “safer sex” messages that spoke exclusively to the use of condoms to include biomedical prevention strategies (e.g.. PEP and PrEP). |
| John, S. A., H. J. Rendina, C. Grov and J. T. Parsons | Home-based pre-exposure prophylaxis (PrEP) services for gay and bisexual men: An opportunity to address barriers to PrEP uptake and persistence; Plos One; 2017; 12 (12); pp. 1-14 | • Health communication and education • Level of knowledge • Prescribing habits and eligibility • Purview Paradox • Relationship with patient • Service structure or model • Staffing and service allocation | • PrEP service aspects, settings and staff • PrEP prescriber experiences, therapeutic alliance and care planning • Multi-disciplinary and innovative PrEP care pathways | The study sought to identify whether GBM in a nationwide cohort who have not yet initiated PrEP (n= 906) would prefer to get PrEP-related care from a primary care provider (PCP) compared to a specialist clinic or provider. The authors found that more than half of participants would prefer to receive PrEP-related care from a PCP; however, nearly three-quarters of men preferred to receive PrEP persistence care via HB-PrEP services. As men who are interested in receiving HB-PrEP would still need to go to a healthcare provider for their first visit for PrEP prescription, these data are relevant for both PrEP uptake and persistence among GBM. In response to the providers’ perspectives, including the “purview paradox” indicating confusion for the best place for patients to receive PrEP-related care, GBM in this sample indicated interest in care from both PCPs and specialists. Meaningful barriers of concern about frequent medical check-ups were associated with preferring a PCP for PrEP-related care, but men who perceived a barrier to bringing up the topic of PrEP with a doctor preferred a specialist clinic or provider more than a PCP. HB-PrEP was more appealing for younger men and those engaged in sexual HIV transmission risk, suggesting HB-PrEP could help reach GBM most vulnerable to HIV and most in need of PrEP. The expansion of HB-PrEP has potential to increase PrEP uptake and persistence among GBM, particularly for men with barriers to clinic-based care and higher intentions to initiate PrEP. |
| Karris, M. Y., S. E. Beekmann, S. R. Mehta, C. M. Anderson and P. M. Polgreen | Are we prepped for preexposure prophylaxis (PrEP)? Provider opinions on the real-world use of PrEP in the United States and Canada; Clincial Infectious Diseases: An Official Publication of the Infectious Diseases of America; 2014; 58 (5); pp. 704-712 | • Prescribing habits and eligibility | • PrEP prescriber experiences, therapeutic alliance and care planning | The study evaluated the current practices and attitudes of PrEP among infectious disease experts who are members of the Emerging Infections Network (EIN). The authors found that strong support exists for PrEP, but very few clinicians (9%) had actually provided it. Additionally, a wide range of PrEP practices existed among those who have or would give PrEP, including differences in deciding who is eligible for PrEP, how persons on PrEP are followed up, and how PrEP is discontinued. Barriers to the provision of PrEP were many, with concerns about PrEP efficacy in the real world being the greatest concern. |
| Klassen, B. J., S. Y. Lin, N. J. Lachowsky, R. S. Hogg, D. M. Moore, E. A. Roth, J. B. Edward and S. A. Chown | Gay Men’s Understanding and Education of New HIV Prevention Technologies in Vancouver, Canada; Qualitative Health Research; 2017; 27 (12); pp. 1775-1791 | • Health communication and education • Level of knowledge • Peer and social networks • Prescribing habits and eligibility • Relationship with patient • Service structure or model • Support services | • PrEP service aspects, settings and staff • PrEP prescriber experiences, therapeutic alliance and care planning • Multi-disciplinary and innovative PrEP care pathways | The study explored the acceptability of New Prevention Technologies (NPT). Participants placed much emphasis on informal education from partners and peers, and the necessity of peer support to effective and acceptable NPT education. Participants mentioned peer support, often in the form of community outreach groups. Thus, peer education, prevention conversations, and support need to be encouraged and fostered within the community, as this will likely expand awareness and use of NPTs. Participants learnt about NPTs through a wide variety of sources including the Internet, healthcare providers, community organizations, sexual partners, and peers. NPT education should incorporate strong, factual information with personal testimony of NPT use, and would balance quality with ease of access. Stigma remains a major barrier to NPT use and education acceptability at various levels, and may be addressed through the promotion of community support and dialogue, along with early adopter testimony, and the challenging of stigma at its structural roots of marginalization. |
| Krakower, D. S., N. C. Ware, K. M. Maloney, I. B. Wilson, J. B. Wong and K. H. Mayer | Differing Experiences with Pre-Exposure Prophylaxis in Boston Among Lesbian, Gay, Bisexual, and Transgender Specialists and Generalists in Primary Care: Implications for Scale-Up; AIDS Patient Care & STDs; 2017; 31 (7); pp. 297-304 | • Health communication and education • Level of knowledge • Prescribing habits and eligibility • Purview Paradox • Service structure or model | • PrEP service aspects, settings and staff • PrEP prescriber experiences, therapeutic alliance and care planning • Multi-disciplinary and innovative PrEP care pathways | The study explored how PCPs approach decisions about prescribing PrEP to MSM and their experiences with PrEP provision. A major finding was that LGBT specialists and generalist PCPs in the same city were at vastly different stages of adopting PrEP into clinical practice 2 years after FDA approval. This has important implications for the scale-up of PrEP nationally. Thus, a deeper understanding of ways to engage generalists in PrEP provision could facilitate wider and more equitable access to PrEP, particularly in areas with limited access to LGBT specialists, such as rural areas. The authors found that LGBT specialists had successfully incorporated PrEP provision for MSM into primary care, whereas generalists in the same locale had limited experience with PrEP. Generalists expressed interest, however, in becoming proficient in PrEP, given their self-perception as preventive medicine experts. Both types of providers articulated similar prescribing dilemmas that may be amenable to solutions based on shared decision-making, which could leverage PCPs’ pre-existing expertise with this decision-making paradigm. |
| Kurtz, S. P. and M. E. Buttram | Misunderstanding of Pre-Exposure Prophylaxis Use Among Men Who Have Sex with Men: Public Health and Policy Implications; 2016; LGBT Health; 3 (6); pp. 461-464 | • Health communication and education | • Multi-disciplinary and innovative PrEP care pathways | The article solicited information about the HIV testing and prevention practices of MSM at high risk for HIV. Of those who had heard of it, few understood PrEP to be a physician-prescribed regimen; most believed it to be a pill taken before and/or after sex and acquired on the street or through HIV-positive friends. National and community-based information campaigns and health navigation resources are critical to ensure that diverse at-risk groups are reached with culturally relevant knowledge. Strategies are needed to overcome other structural barriers to efficacious PrEP uptake, including the standardization of insurance coverage, and a reduction in cost disparities across the high-risk groups for whom PrEP could dramatically reduce the number of new infections. |
| Kwakwa, H. A., S. Bessias, D. Sturgis, G. Walton, R. Wahome, O. Gaye and M. Jackson | Engaging United States Black Communities in HIV Pre-exposure Prophylaxis: Analysis of a PrEP Engagement Cascade; Journal of National Medical Association; 2018; 110 (5); pp. 480-485 | • Health communication and education • Prescribing habits and eligibility • Referral • Service structure or model | • PrEP service aspects, settings and staff • Multi-disciplinary and innovative PrEP care pathways | The article examined the process of accessing PrEP for a majority Black population in an urban community health center setting. In this study, the lower percentage of high-risk referred patients who completed the access process and initiated PrEP suggests a need for support and facilitation at each step of the PrEP engagement cascade, particularly for populations with steeper fall-off such as those who have never heard of PrEP, non-Hispanic Black MSM and women. It is critical that the necessary effort be invested in engaging this community despite a potentially greater degree of difficulty. If health services fail to improve PrEP utilisation among racial/ethnic minorities, existing disparities in HIV prevalence stand to grow. |
| Landovitz, R. J., M. Beymer, R. Kofron, K. R. Amico, C. Psaros, L. Bushman, P. L. Anderson, R. Flynn, D. P. Lee, R. K. Bolan, W. C. Jordan, C. Tseng, R. Dierst-Davies, J. Rooney and A. R. Wohl | Plasma Tenofovir Levels to Support Adherence to TDF/FTC Preexposure Prophylaxis for HIV Prevention in MSM in Los Angeles, California; Journal of Acquired Immune Deficiency Syndromes; 2017; 76 (5); pp. 501-511 | • Service structure or model | • PrEP service aspects, settings and staff | The authors explored strategies that identify and intervene with those challenged by adherence to daily medication of PrEP. Results showed that 75.1% of participants were retained in the PrEP cohort and 65.5% maintained adherence up to week 48. Younger and African-American participants were less likely to have protective drug levels. The study showed that PrEP was acceptable and well tolerated in a diverse population of MSM and that Drug level monitoring has the potential to allow targeting of additional adherence support to those struggling with daily tablet adherence. |
| Lelutiu-Weinberger, C. and S. A. Golub | Enhancing PrEP Access for Black and Latino Men Who Have Sex With Men; Journal of Acquired Immune Deficiency Syndromes; 2016; 73 (5); pp. 547-555 | • Health communication and education • Relationship with patient • Social media, apps and technology • Support services | • PrEP service aspects, settings and staff • PrEP prescriber experiences, therapeutic alliance and care planning • Multi-disciplinary and innovative PrEP care pathways | The study examined the differences in perceived barriers and facilitators to PrEP access for Black and Latino MSM compared to other MSM. Compared to other MSM, Black and Latino MSM (56% of the sample) were more likely to have public insurance and access health care via public clinics; were more likely to regard having to talk to their doctor about their sex life as a barrier to PrEP; less likely to endorse agency in medical decision-making; more likely to report PrEP stigma and concerns regarding PrEP efficacy; were more likely to consider access to free sexual health care and additional supportive services, e.g., counseling or text-based support. Increasing interest in PrEP may involve high-quality education about PrEP efficacy, and increased engagement by trusted community members and representatives. |
| Levy, M. E., C. C. Watson, S. N. Glick, I. Kuo, L. Wilton, R. A. Brewer, S. D. Fields, V. Criss and M. Magnus | Receipt of HIV prevention interventions is more common in community-based clinics than in primary care or acute care settings for Black men who have sex with men in the District of Columbia; AIDS Care - Psychological and Socio-Medical Aspects of AIDS/HIV; 2016; 28 (5); pp. 660-664 | • Relationship with patient • Staffing and service allocation | • PrEP prescriber experiences, therapeutic alliance and care planning | The study investigates the utilisation of HIV prevention and general care services among a non clinic-based sample of Black MSM. The majority of men (76%) had accessed primary care, acute care, or a community-based clinic in the last six months despite barriers to care. However, there were disparities by setting in the provision of services to Black MSM, with greater receipt of HIV prevention services at community-based clinics than in primary care or acute care settings. Conversely, general preventive services were obtained more frequently in primary care settings than at community-based clinics. Black MSM were less likely than other MSM to disclose sexual behaviors to providers, and those who do not disclose sexual behaviors are less likely to discuss HIV, disclose their HIV status, and obtain HIV testing. Only half of HIV-negative participants were offered an HIV test. |
| Liu, A. Y., E. Vittinghoff, P. von Felten, K. R. Amico, P. L. Anderson, R. Lester, E. Andrew, I. Estes, P. Serrano, J. Brothers, S. Buchbinder, S. Hosek and J. D. Fuchs | Randomized Controlled Trial of a Mobile Health Intervention to Promote Retention and Adherence to Pre-exposure Prophylaxis among Young People at Risk for Human Immunodeficiency Virus: The EPIC Study; Clinical Infectious Diseases: An Official Publication of the Infectious Diseases Society of America; 2018 | • Social media, apps and technology | • PrEP adherence within formal service structures • Multi-disciplinary and innovative PrEP care pathways | The study evaluate the impact of a youth-tailored, bidirectional text-messaging intervention (PrEPmate) on study retention and PrEP adherence. Participants who received PrEPmate were more likely to be retained at study visits and achieve protective PrEP levels over 36 weeks. The beneficial impact of PrEPmate did not differ significantly by baseline characteristics including age, race/ethnicity, education, and insurance. Although rates of sexual risk behaviors and STIs were high at baseline, overall risk declined in the study, with similar declines observed in both arms. Bidirectional SMS-based PrEP support was found to be active in increasing PrEP retention and adherence among youth at risk for HIV. Future implementation science research is needed to evaluate the impact of PrEPmate when implemented in more diverse geographic and clinic settings, including internationally, and assess contextual factors which may influence the implementation, effectiveness, and scale-up of PrEPmate. |
| Maloney, K. M., D. S. Krakower, D. Ziobro, J. G. Rosenberger, D. Novak and K. H. Mayer | Culturally Competent Sexual Healthcare as a Prerequisite for Obtaining Preexposure Prophylaxis: Findings from a Qualitative Study; LGBT Health; 2017; 4 (4); pp. 310-314 | • Health communication and education • Level of knowledge • Relationship with patient | • PrEP service aspects, settings and staff • PrEP prescriber experiences, therapeutic alliance and care planning • Multi-disciplinary and innovative PrEP care pathways | The authors aimed to explore factors influencing discussions about PrEP between MSM and their healthcare providers. Participants identified PCPs as the preferred source for information about PrEP. However, an emergent theme was the need for a non-judgmental relationship with a PCP before disclosure of sexual orientation and discussions about sexual health, including HIV prevention and PrEP, could occur. Online focus groups with geographically diverse members of a partner- seeking website for MSM suggest that barriers to communicating about sexual health between MSM and providers may be limiting access to PrEP and could exacerbate inequities in PrEP uptake. Improved patient–provider communication about sexual orientation and sexual behaviors might increase PrEP use among MSM, particularly for MSM without access to LGBT-specialized providers. |
| Marcus, J. L., L. B. Hurley, C. B. Hare, D. P. Nguyen, T. Phengrasamy, M. J. Silverberg, J. E. Stoltey and J. E. Volk | Preexposure Prophylaxis for HIV Prevention in a Large Integrated Health Care System: Adherence, Renal Safety, and Discontinuation; JAIDS: Journal of Acquired Deficiency Syndrome; 2016; 73 (5); pp. 540-546 | • Social media, apps and technology • Support services | • PrEP adherence within formal service structures • Multi-disciplinary and innovative PrEP care pathways | The study assessed pharmacy refill adherence and discontinuation, decreases in estimated glomerular filtration rate (eGFR), and sexually transmitted infection (STI)/HIV incidence. The authors observed no HIV infections during 850 person-years of ongoing PrEP use. Adherence was 92% overall; however, Black race/ethnicity, higher TDF/FTC co-payments, and smoking were associated with lower adherence. They found that PrEP is being accessed by a primarily male, White, and older population, indicating that outreach is needed to others at risk for HIV infection, including female, Black, and younger individuals. Adherence is high, but strategies are needed to increase affordability, facilitate PrEP access during gaps in insurance coverage, and support adherence. |
| Marcus, J. L., K. Levine, C. Grasso, D. S. Krakower, V. Powell, K. T. Bernstein, S. Boswell and K. H. Mayer | HIV Preexposure Prophylaxis as a Gateway to Primary Care; American Journal of Public Health; 2018; 108 (10); pp. 1418-1420 | • Support services | • Multi-disciplinary and innovative PrEP care pathways | The authors determine whether PrEP use is associated with use of non–HIV-related health care. They found that PrEP use was independently associated with increased receipt of primary care, including influenza vaccination, tobacco and depression screening, and glucose testing, but not haemoglobin A1c testing. The benefits of PrEP may extend to behavioral health, mental health, and prevention and treatment of other infectious and chronic diseases. In addition to efforts to integrate PrEP prescribing into primary care, efforts may be warranted to ensure uptake of recommended primary care among PrEP users. |
| Marks, S. J., R. C. Merchant, M. A. Clark, T. Liu, J. G. Rosenberger, J. Bauermeister and K. H. Mayer | Potential Healthcare Insurance and Provider Barriers to Pre-Exposure Prophylaxis Utilization Among Young Men Who Have Sex with Men; AIDS Patient Care & STDs; 2017; 31 (11); pp. 470-478 | • Prescribing habits and eligibility | • PrEP service aspects, settings and staff | The study examined potential healthcare insurance and provider barriers to PrEP utilisation among YMSM. As expected, PrEP use was associated with higher levels of condomless sex, access to health insurance, and access to a primary healthcare provider. The authors state that although PrEP offers an effective and safe means of intervention, utilization remains lower than is required to curb the HIV epidemic. Findings identified disparities in access to healthcare by age, race/ethnicity, education, and region. Specifically, older YMSM, blacks and Hispanics, those with fewer years of formal education, and residents of the southern and the western United States were more likely to lack healthcare access. |
| Mayer, K., S. Safren, S. Elsesser, C. Psaros, J. Tinsley, M. Marzinke, W. Clarke, C. Hendrix, S. Wade Taylor, J. Haberer and M. Mimiaga | Optimizing Pre-Exposure Antiretroviral Prophylaxis Adherence in Men Who Have Sex with Men: Results of a Pilot Randomized Controlled Trial of 'Life-Steps for PrEP'; AIDS & Behavior; 2017; 5; pp. 1350-1360 | • Staffing and service allocation • Support services • Tailoring | • PrEP service aspects, settings and staff • Multi-disciplinary and innovative PrEP care pathways | The study evaluated a cognitive behavioral intervention condition or a time and session-matched comparison counseling intervention. Findings showed that adherence was high for both the intervention and the controlled group. However, plasma tenofovir levels were significantly higher in teh intervention group at 6 months, yet at completion of the study, this was not significant. Medication adherence was high across a cognitive-behavioral (Life-Steps) and time-matched counseling intervention for PrEP adherence, with some evidence suggesting superiority of Life-Steps in this pilot RCT. |
| Merchant, R. C., D. Corner, E. Garza, W. Guan, K. H. Mayer, L. Brown and P. A. Chan | Preferences for HIV pre-exposure prophylaxis (PrEP) information among men-who-have-sex-with-men (MSM) at community outreach settings; Journal of Gay & Lesbian Mental Health; 2016; 20 (1); pp. 21-33 | • Health communication and education • Relationship with patient • Service structure or model • Social media, apps and technology | • Multi-disciplinary and innovative PrEP care pathways | The article explored the interest in learning more about PrEP and preferences in receiving information about PrEP among HIV-uninfected MSM. The authors found that observed that among MSM recruited through community outreach, HIV sexual risk-taking was significant, yet self-perceived PrEP knowledge was low and interest in learning more about PrEP was moderate. Most preferred learning about PrEP and being provided local PrEP clinic information through electronic media. However, receipt of PrEP information alone did not appear to motivate these men into presenting to a local clinic for PrEP evaluation. They advocated for an increased effort to educate MSM in the community about HIV PrEP preferentially through electronic outlets. Primary care providers should feel comfortable addressing the topic with their patients, and public health efforts should focus on production of electronic educational media with an emphasis on linkage to care. |
| Mitchell, J. T., S. LeGrand, L. B. Hightow-Weidman, M. S. McKellar, A. D. M. Kashuba, M. Cottrell, T. McLaurin, G. Satapathy and F. J. McClernon | Smartphone-Based Contingency Management Intervention to Improve Pre-Exposure Prophylaxis Adherence: Pilot Trial; JMIR Mhealth and Uhealth; 2018; 6 (9); p. e10456 | • Social media, apps and technology | • PrEP adherence within formal service structures • Multi-disciplinary and innovative PrEP care pathways | The authors developed and pilot-tested a smartphone-based intervention, known as mSMART, that targets PrEP adherence. mSMART is the first PrEP adherence intervention administered via smartphones to integrate contingency management. The authors found that adherence scored improved for 30% of participants, and adherence did not worsen for any participant. Although our findings indicate that mSMART is a promising intervention to improve adherence rates, the results are preliminary and future studies are needed to demonstrate efficacy. These studies should also consider our findings indicating areas in which mSMART can be adapted to more comprehensively meet the needs of young MSM prescribed PrEP. |
| Mullins, T. L. K., G. Zimet, M. Lally, J. Xu, S. Thornton and J. A. Kahn | HIV Care Providers' Intentions to Prescribe and Actual Prescription of Pre-Exposure Prophylaxis to At-Risk Adolescents and Adults; AIDS Patient Care & STDs; 2017; 31 (12); pp. 504-516 | • Level of knowledge • Prescribing habits and eligibility • Purview Paradox • Service structure or model • Staffing and service allocation • Support services | • PrEP service aspects, settings and staff | The authors explored intentions to prescribe PrEP to adolescents and adults and sought to identify the actual prescription of PrEP. They found that clinicians reported higher intention to prescribe, and more experience prescribing, PrEP to adults versus adolescents, and several modifiable factors, such as provider-level barriers and concerns about cost and insurance coverage, were associated with intention to prescribe PrEP to youth. Clinicians reported greater intention to prescribe PrEP to adults compared with adolescents, and the authors identified a number of modifiable factors associated with intention to prescribe and actual prescription of PrEP to adolescents that should be addressed to increase provision of PrEP to youth. Furthermore, they found that multi-disciplinary teams and behavioral interventions are not necessary for delivering PrEP and that this was associated with intention to prescribe and actual prescription of PrEP suggests that clinicians view these elements as barriers to prescribing PrEP. To address this potential barrier to PrEP prescription, brief, streamlined, and effective behavioral interventions that can be delivered with PrEP need to be developed and disseminated. |
| Mutua, G., E. Sanders, P. Mugo, O. Anzala, J. E. Haberer, D. Bangsberg, B. Barin, J. F. Rooney, D. Mark, P. Chetty, P. Fast and F. H. Priddy | Safety and adherence to intermittent pre-exposure prophylaxis (PrEP) for HIV-1 in African men who have sex with men and female sex workers; Plos On; 2012; 7 (4); p. e33103 | • Service structure or model • Social media, apps and technology | • PrEP service aspects, settings and staff • Multi-disciplinary and innovative PrEP care pathways | The article examined safety and adherence data from the first trial of an intermittent PrEP regimen among Kenyan MSM and FSW. The data suggest that although adherence is lower with fixed intermittent than with daily regimens, whilst adherence to post-coital doses was 26%. Additional drug level data, qualitative data on adherence barriers, and better methods to measure sexual activity are necessary to determine whether adherence to post-coital PrEP could be comparable to more standard regimens. |
| Newman, P. A., A. Guta, A. Lacombe-Duncan and S. Tepjan | Clinical exigencies, psychosocial realities: negotiating HIV pre-exposure prophylaxis beyond the cascade among gay, bisexual and other men who have sex with men in Canada; Journal of the International AIDS Society; 2018; 21 (11); p. e25211 | • Health communication and education • Peer and social networks • Service structure or model • Social media, apps and technology • Staffing and service allocation | • PrEP service aspects, settings and staff • Multi-disciplinary and innovative PrEP care pathways | The authors sought to understand the experiences of users and non-users of PrEP. Regardless of PrEP use, participants generally described having multiple partners, using condoms inconsistently and employing a range of strategies for managing sexual risk, including condom use. Some participants who had not initiated PrEP use described HIV risk behaviours and the use of testing, but demonstrated low perceived risk and no anxiety about acquiring HIV. All PrEP users and some non-users reported seeking out information in scientific (e.g. academic journals) and/or community-based sources (AIDS service organization websites). Most participants who sought out PrEP did not report barriers in linkage to care nor discriminatory reactions from healthcare providers. PrEP access was facilitated by an urban environment with many LGBTQ-friendly services and physicians, many of whom also provide care to people living with HIV. PrEP users described various strategies for taking their medication, often borne of trial-and-error, while non-PrEP users generally anticipated that adherence would not be a concern. Other findings covered retention, discontinuation, stigma and the impact of PrEP on sexual practices and relationships. |
| Ojikutu, B., L. Bogart, K. Mayer, T. Stopka, P. Sullivan and Y. Ransome | Spatial Access and Willingness to Use Pre-Exposure Prophylaxis Among Black/African American Individuals in the United States: Cross-Sectional Survey; JMIR Public Health and Surveillance; 2019; 5 (1) | • Health communication and education • Peer and social networks • Support services | • PrEP service aspects, settings and staff | The study determined the association between proximity to PrEP-prescribing clinics and willingness to use PrEP among black individuals. The findings demonstrate that black individuals with higher spatial access to PrEP-prescribing clinics were more willing to use this intervention. Scaling up of PrEP prescription at clinics in areas where black individuals reside is necessary to increase access to PrEP. |
| Ojile, N., D. Sweet and K. J. Kallail | A Preliminary Study of the Attitudes and Barriers of Family Physicians to Prescribing HIV Preexposure Prophylaxis; Kansas Journal of Medicine; 2017; 10 (2); pp. 40-42 | • Health communication and education • Level of knowledge • Prescribing habits and eligibility • Purview Paradox • Relationship with patient • Staffing and service allocation | • PrEP service aspects, settings and staff • Multi-disciplinary and innovative PrEP care pathways | The authors assessed the attitudes and perceived barriers of family physicians in Kansas towards prescribing PrEP to high risk patient populations. Fifty-three percent of family physicians take a sexual history on new patients less than frequently, and only 35% frequently ask about the use of safe sex practices. Only 29% frequently ask if the patient has sex with men, women, or both. Seventy-six percent of respondents would be willing to prescribe PrEP. While 59% of participants agreed that PrEP belongs in the primary care domain of treatment, 71% agreed that they had limited or no knowledge of PrEP guidelines. |
| Paparini, S., W. Nutland, T. Rhodes, V. Nguyen and J. Anderson | DIY HIV prevention: Formative qualitative research with men who have sex with men who source PrEP outside of clinical trials; Plos One; 2018; 13 (8); p. e0202830 | • Health communication and education • Peer and social networks • Service structure or model • Staffing and service allocation • Support services | • PrEP service aspects, settings and staff • Multi-disciplinary and innovative PrEP care pathways | The articles aims to understand the information and support needs of MSM who currently are, or who have recently, self-obtained and self-administered PrEP. The authors found that DIY PrEP users are highly motivated “early adopters” who are keen and able to experiment with new strategies for HIV prevention, even in the context of partial information and the need for significant personal motivation. Yet the discussions also highlighted that participants depend on crucial peer support to obtain a prevention technology that was promoted through community engagement in clinical trials rather than as a public health intervention. This highlights the need for tailored interventions as well as further research and analysis to better understand how DIY PrEP users can be supported to ensure the best possible outcomes from these informal practices. Understanding how to harness community activism and clinical partnerships in different contexts remains key to maximizing the HIV prevention impact of informal PrEP. The findings suggest that participants felt that the diffusion of information on how to access PrEP was initiated and sustained by their communities. |
| Parisi, D., B. Warren, S. J. Leung, T. Akkaya-Hocagil, Q. Qin, I. Hahn and L. Stevens | A Multicomponent Approach to Evaluating a Pre-exposure Prophylaxis (PrEP) Implementation Program in Five Agencies in New York; JANAC; Journal of the Association of Nurses in AIDS Care; 2018; 29 (1) | • Health communication and education • Referral • Relationship with patient • Service structure or model • Social media, apps and technology • Staffing and service allocation • Support services | • PrEP service aspects, settings and staff • PrEP adherence within formal service structures • Multi-disciplinary and innovative PrEP care pathways | The study determined the practicality of using PrEP as an HIV prevention intervention for adult MSM who were at high risk for acquiring HIV infection. Findings showed that 86% of clients assessed for eligibility were enrolled in TPIP. Participants found it easy to take a pill every day, attend routine medical visits, test for HIV and STIs, and deal with the side effects of PrEP. Although 77% of participants did not have insurance issues, participants who were uninsured and underinsured encountered issues with high deductibles/copayments and lack of coverage for medical visits and testing. TPIP staff stressed the necessity of having a PrEP navigator who could assist clients with resources, such as assistance with paying for medications, medical visits, and testing. TPIP demonstrated that clients could adhere to PrEP and have positive outcomes. Agency feedback demonstrated the need for an agency-wide implementation strategy to effectively develop a PrEP program. TPIP played a pivotal role in laying the foundation for statewide implementation of PrEP. Throughout the program, PrEP was delivered as part of a comprehensive prevention plan that included consistent and correct condom use, safer-sex practices, risk- reduction counseling, and routine screening for HIV and other STIs that could facilitate HIV transmission. |
| Parsons, J. T., S. A. John, T. H. F. Whitfield, J. Cienfuegos-Szalay and C. Grov | HIV/STI counseling and testing services received by gay and bisexual men using pre-exposure prophylaxis (PrEP) at their last PrEP care visit; Sexually Transmitted Diseases; 2018; 45 (12); pp. 798-802 | • Health communication and education • Purview Paradox • Relationship with patient • Service structure or model • Staffing and service allocation • Support services | • PrEP service aspects, settings and staff • PrEP prescriber experiences, therapeutic alliance and care planning | The study examined where PrEP-using GBM in New York City access PrEP care services, and determine the comprehensiveness of their routine PrEP maintenance care. The findings highlighted that there is also a need for reducing barriers to the provision of comprehensive PrEP care services to patients. The majority of PrEP-using MSM did not receive comprehensive care consisting of a discussion about their sexual behavior, blood and urine samples, and both rectal and oral swabs at their last PrEP care visit. Compared to men who more recently initiated PrEP, those who have been on PrEP longer, engaged in more CAS, and received care from a specialized clinic were more likely to receive comprehensive care. |
| Pasipanodya, E. C., S. Jain, X. Sun, J. Blumenthal, E. Ellorin, K. Corado, M. P. Dube, E. S. Daar, S. R. Morris and D. J. Moore | Trajectories and Predictors of Longitudinal Preexposure Prophylaxis Adherence Among Men Who Have Sex With Men; Journal of Infectious Diseases; 2018; 218 (10); pp. 1551-1559 | • Social media, apps and technology • Support services | • PrEP service aspects, settings and staff • PrEP adherence within formal service structures • Multi-disciplinary and innovative PrEP care pathways | The study sought to identify trajectories of PrEP adherence among individuals receiving adherence texts, explore factors associated with patterns of adherence, and examine study-end attitudes toward PrEP and the text-messaging intervention. The model identified that classes with higher text-reported adherence had higher drug concentrations. Younger age and minority race were associated with lower adherence, and individuals in classes with lower adherence had greater baseline levels of depression, substance use concerns, and sexual risk. |
| Patel, R. R., P. A. Chan, L. C. Harrison, K. H. Mayer, A. Nunn, L. A. Mena and W. G. Powderly | Missed Opportunities to Prescribe HIV Pre-Exposure Prophylaxis by Primary Care Providers in Saint Louis, Missouri; LGBT Health; 2018; 5 (4); pp. 250-256 | • Health communication and education • Level of knowledge • Prescribing habits and eligibility • Purview Paradox • Referral • Relationship with patient • Service structure or model • Staffing and service allocation | • PrEP service aspects, settings and staff • PrEP prescriber experiences, therapeutic alliance and care planning • Multi-disciplinary and innovative PrEP care pathways | The authors explored whether patients seeking PrEP at an ID specialty clinic were engaged in primary care, if they had asked their PCPs about PrEP before seeking specialty care, and to identify the reasons why patients did not ask their PCPs for PrEP and why PCPs had declined to prescribe PrEP. Findings highlighted that individuals seeking PrEP faced numerous barriers to obtaining PrEP from PCPs in metropolitan Saint Louis, Missouri. Major barriers included not having a PCP, having a PCP who was unaware of and/or perceived PrEP to be a specialist’s responsibility, not knowing a PCP could prescribe PrEP, and having a PCP but not feeling comfortable discussing sexual behaviors. Forty-eight percent of these at-risk patients asked for PrEP from their PCPs, but were not prescribed it, creating a large missed PrEP- prescribing opportunity by PCPs. |
| Phanuphak, N., T. Sungsing, J. Jantarapakde, S. Pengnonyang, D. Trachunthong, P. Mingkwanrungruang, W. Sirisakyot, P. Phiayura, P. Seekaew, P. Panpet, P. Meekrua, N. Praweprai, F. Suwan, S. Sangtong, P. Brutrat, T. Wongsri, P. R. Na Nakorn, S. Mills, M. Avery and R. Vannakit | Princess PrEP program: the first key population-led model to deliver pre-exposure prophylaxis to key populations by key populations in Thailand; Sexual Health; 2018; 15 (6); pp. 542-555 | • Referral • Service structure or model • Social media, apps and technology • Staffing and service allocation | • PrEP service aspects, settings and staff • PrEP adherence within formal service structures • Multi-disciplinary and innovative PrEP care pathways | The article investigates the uptake, retention and adherence to PrEP services from the Princess PrEP program implemented by trained MSM and M2F TG community health workers in community health centres throughout Thailand. Findings showed that age >25 years, being MSM and having at least a Bachelors degree significantly increased retention to PrEP and condomless sex did not change over the 12-month period. Retention in PrEP programs is a concern, especially among TGW and those who were young or with lower levels of education; innovative tools are needed to support these populations. STI screening and treatment infrastructures must be immediately strengthened and programs and health systems should advocate for access to cheaper molecular-based STI testing. To scale-up and sustain KP-led PrEP programs, strong endorsement from international and national guidelines is necessary. |
| Philbin, M. M., C. M. Parker, R. G. Parker, P. A. Wilson, J. Garcia and J. S. Hirsch | Gendered Social Institutions and Preventive Healthcare Seeking for Black Men Who Have Sex with Men: The Promise of Biomedical HIV Prevention; Archives of Sexual Behavior; 2018; 7; pp. 2091-2100 | • Level of knowledge • Relationship with patient • Service structure or model | • PrEP service aspects, settings and staff • PrEP prescriber experiences, therapeutic alliance and care planning | The study examined the healthcare system and the labor market as two gendered institutions that influence Black MSM’s preventive healthcare practices and show how Black MSM are negatively impacted by heteronormatively gendered institutional processes in health care and labor market settings. Two primary findings emerged from the study. The first is that the labor market systematically excluded the men in the sample, which limited their ability to access employer-sponsored healthcare. Such discrimination may promote overt demonstrations of masculinity that increase their HIV vulnerability and decrease healthcare seeking. Secondly, healthcare systems are not structured to promote preventive healthcare for men, particularly Black MSM. In fact, they constrained men’s access to primary providers and were usually tailored to women. Applying a structural, gendered lens to men’s health—in addition to the more frequently researched individual or interpersonal levels—provides insight into factors that affect healthcare seeking and HIV prevention for Black MSM. |
| Raifman, J., A. Nunn, C. E. Oldenburg, M. C. Montgomery, A. Almonte, A. L. Agwu, R. Arrington‐Sanders, P. A. Chan and R. Arrington-Sanders | An Evaluation of a Clinical Pre-Exposure Prophylaxis Education Intervention among Men Who Have Sex with Men; Health Services Research; 2018; 53 (4); 2249-2267 | • Health communication and education • Staffing and service allocation | • PrEP prescriber experiences, therapeutic alliance and care planning • Multi-disciplinary and innovative PrEP care pathways | The authors evaluated the impact of PrEP education intervention on PrEP awareness and use among MSM attending a STD clinic. The intervention increased PrEP awareness by 24% and increased PrEP use by 5%. There is a need for interventions to increase PrEP awareness and use, especially among MSM who are racial and ethnic minorities in STD clinics, primary care practices, and other settings providing sexual health care. |
| Raifman, J. R. G., C. Flynn and D. German | Healthcare Provider Contact and Pre-exposure Prophylaxis in Baltimore Men Who Have Sex With Men; American Journal of Preventive Medicine; 2017; 52 (1); 55-63 | • Health communication and education • Level of knowledge • Relationship with patient • Service structure or model • Support services | • PrEP service aspects, settings and staff • PrEP prescriber experiences, therapeutic alliance and care planning | The article determined whether healthcare provider or CBC contact was associated with PrEP awareness among MSM. Findings highlight the need to support healthcare providers’ PrEP knowledge and ability to discuss PrEP with key populations at risk of HIV as well as to address racial disparities in PrEP awareness. Policymakers can also improve PrEP implementation by encouraging government organisations and other professional groups to issue PrEP recommendations. |
| Refugio, O. N., M. M. Kimble, C. L. Silva, J. E. Lykens, C. Bannister and J. D. Klausner | PrEPTECH: a telehealth-based initiation program for human immunodeficiency virus pre-exposure prophylaxis in young men of color who have sex with men. A pilot study of feasibility; Journal of Acquired Immune Deficiency Syndromes; 2018; 80; pp. 40-45 | • Relationship with patient • Social media, apps and technology | • Multi-disciplinary and innovative PrEP care pathways | The study investigated the feasibility of PrEPTECH, an intervention that uses telehealth to initiate and deliver PrEP medication, as well as promote PrEP adherence. PrEPTECH provides unique services that differ from the few other telehealth-based PrEP programs, including customizable daily reminders and online education. The study reported that at least 75% felt that PrEPTECH was confidential, fast, convenient, and easy to use. Less than 15% personally experienced PrEP stigma during the study. The median time to PrEP initiation was 46 days. Sexually transmitted infection positivity was 20% and 19% at baseline and 90 days, respectively. No HIV infections were detected. |
| Ridgway, J. P., E. A. Almirol, A. Bender, A. Richardson, J. Schmitt, E. Friedman, N. Lancki, I. Leroux, N. Pieroni, J. Dehlin and J. A. Schneider | Which Patients in the Emergency Department Should Receive Preexposure Prophylaxis? Implementation of a Predictive Analytics Approach; AIDS Patient Care and STDs; 2018; 32 (5); pp. 202-207 | • Social media, apps and technology • Staffing and service allocation • Support services | • Multi-disciplinary and innovative PrEP care pathways | The authors developed and implemented an electronic risk score to identify Emergency Department patients who are potential candidates for PrEP. The study found that of the 51 patients who completed risk assessment, 68.6% (35/51) were interested in PrEP, 17.6% (9/51) scheduled a PrEP appointment, and 7.8% (4/51) successfully initiated PrEP. The measured number of successful PrEP initiations is likely an underestimate, as it does include patients who initiated PrEP with outside providers or referred acquaintances for PrEP care. It was concluded that individuals who seek care in the Emergency Department are often at disproportionate risk for HIV. They have the potential to play a crucial role in HIV prevention by identifying and linking high-risk HIV-negative clients to PrEP care and require further research to outline the future role in PrEP service provision. |
| Rivierez, I., G. Quatremere, B. Spire, J. Ghosn and D. Rojas Castro | Lessons learned from the experiences of informal PrEP users in France: results from the ANRS-PrEPage study; AIDS Care; 2018; 30; pp. 48-53 | • Support services | • Multi-disciplinary and innovative PrEP care pathways | The study explored the subjective experience and the underlying logic behind informal PrEP use through a community-based approach. The results indicate that avoiding transmission while improving the quality of sex-life was essential for participants allowed them to overcome legal barriers to obtain PrEP. However, despite a proximity to well-informed groups, some jeopardized their health by adopting wrong regimens or by using drugs that were not approved for PrEP. The results support the need for a harmonized European AIDS policy around PrEP and provide information for the support of PrEP use in future implementation programs. |
| Rusie, L. K., C. Orengo, D. Burrell, A. Ramachandran, M. Houlberg, K. Keglovitz, D. Munar and J. A. Schneider | Preexposure Prophylaxis Initiation and Retention in Care Over 5 Years, 2012-2017: Are Quarterly Visits Too Much?; Clinical Infectious Diseases; 2018; 67 (2); 283-287 | • Staffing and service allocation | • PrEP adherence within formal service structures | The article provides descriptive information on retention in care patterns in routine clinical care and to explore some of the baseline factors related to retention that may inform clinical practice, mathematical models, and future retention in PrEP care prevention interventions. The authors reported that with respect to PrEP retention in care, just under half (43%) were retained in care for ≥12 months, yet only 15% had high visit constancy (clinic visit in all 4 quarters) consistent with CDC guidelines during the first 12 months of PrEP care. Factors associated with PrEP visits in ≥1 quarter included the number of other comorbid conditions and insurance. |
| Shover, C. L., M. Javanbakht, S. Shoptaw, R. K. Bolan, L. Sung-Jae, J. T. Parsons, J. Rendina and P. M. Gorbach | HIV Preexposure Prophylaxis Initiation at a Large Community Clinic: Differences Between Eligibility, Awareness, and Uptake; American Journal of Public Health; 2018; 108 (10); pp. 1409-1417 | • Prescribing habits and eligibility • Service structure or model | • PrEP service aspects, settings and staff | The article characterized the uptake of PrEP in a community setting and to identify disparities in PrEP use by demographic and behavioral factors associated with increased HIV risk. The findings suggest that whilst there are disparities in PrEP use among young MSM and transgender people of color, PrEP uptake is increasing generally. As PrEP is acceptable to those who use sex drugs, interventions providing PrEP services including retention and adherence support targeting these individuals have the potential to reduce HIV transmission. |
| Siegler, A. J., A. Bratcher, K. M. Weiss, F. Mouhanna, L. Ahlschlager and P. S. Sullivan | Location location location: an exploration of disparities in access to publicly listed pre-exposure prophylaxis clinics in the United States; Annals of Epidemiology; 2018 | • Service structure or model • Social media, apps and technology • Staffing and service allocation | • PrEP service aspects, settings and staff | The authors described the geographic distribution of PrEP clinics in the United States. They also explored how the density of PrEP-providing clinics aligns with race, income, insurance status, and urban city in comparison to the overall population, to estimated numbers of MSM eligible for PrEP, and to new HIV diagnoses. Findings reported that most (43/50) states had less than one PrEP-providing clinic per 100,000 population. Among states, the median was two clinics per 1000 PrEP-eligible men who have sex with men. Differences between disease burden and service provision were seen for counties with higher proportions of their residents living in poverty, lacking health insurance, identifying as African American, or identifying as Hispanic/Latino. The Southern region accounted for over half of all new HIV diagnoses but only one-quarter of PrEP-providing clinics. |
| Smith, D. K., L. Toledo, D. J. Smith, M. A. Adams and R. Rothenberg | Attitudes and Program Preferences of African-American Urban Young Adults About Pre-Exposure Prophylaxis (PrEP); AIDS Educaiton & Prevention; 2012; 24 (5); pp. 408-421 | • Staffing and service allocation | • PrEP adherence within formal service structures | The article to elicits attitudes about, and preferences for, PrEP services from a key group of potential users in the United States. The authors focus on inner-city, African-American young adult men and women at risk for HIV transmission because of their sexual and drug-related behaviors. In these focus groups of young African-American men and women, substantial interest in PrEP was reported among both heterosexuals and MSM. Interest in PrEP was associated with its cost, effectiveness, and ease of accessing services and medication near to their homes or by public transportation. The successful introduction of clinically delivered HIV prevention methods for African-American young adults at risk of HIV acquisition requires an understanding not only of their current beliefs about HIV acquisition and its related sexual risk and protective behaviors, but also their beliefs about medication use and challenges and opportunities related to their access to health care. |
| Spinelli, M. A., H. M. Scott, E. Vittinghoff, A. Y. Liu, A. Morehead-Gee, R. Gonzalez and S. P. Buchbinder | Provider adherence to pre-exposure prophylaxis monitoring guidelines in a large primary care network; Open Forum Infectious Diseases; 2018; 5 (6) | • Prescribing habits and eligibility • Support services | • PrEP service aspects, settings and staff • Multi-disciplinary and innovative PrEP care pathways | The study examined test ordering of PrEP in a primary care network. The authors found that providers did not order HIV testing before almost one-quarter of PrEP initiations and that panel management was associated with higher testing. Although they discovered suboptimal HIV and STI testing in a primary care population, data suggest the promise of panel management, which could address disparities in PrEP testing. Future research into innovative population management strategies could help minimize PrEP’s potential risks and maximize its preventive impact. |
| Stekler, J. D., V. McMahan, L. Ballinger, L. Viquez, F. Swanson, J. Stockton, B. Crutsinger-Perry, D. Kern and J. D. Scott | HIV Pre-exposure Prophylaxis Prescribing Through Telehealth; Journal of Acquired Immune Deficiency Syndromes; 2018; 77 (5); pp. e40-e42 | • Social media, apps and technology | • PrEP adherence within formal service structures • Multi-disciplinary and innovative PrEP care pathways | The article described our preliminary experience using a telehealth approach to prescribe PrEP. The program showed preliminary evidence of the feasibility and acceptability of a telehealth approach to PrEP prescribing that promotes HIV counselors as PrEP experts. The approach is flexible as it provides to the specialist and the transition of time and effort to less-expensive staff who are content experts in HIV and sexual health. Telehealth participants had similar baseline characteristics compared with other participants, but they were significantly less likely to return for their 3-month follow-up. Although conceivable this was due to the physical absence of the physician at the initial visit, it is more likely associated with participant factors that led to the client choosing to be seen on an alternate day. Work is needed to develop strategies to promote PrEP adherence and persistence among persons at highest risk of HIV acquisition and identify individuals most likely to benefit from those strategies. |
| Sullivan, P. S., R. Driggers, J. D. Stekler, A. J. Siegler, T. Goldenberg, S. J. McDougal, J. Caucutt, J. Jones and R. Stephenson | Usability and Acceptability of a Mobile Comprehensive HIV Prevention App for Men Who Have Sex With Men: A Pilot Study; JMIR Mhealth and Uhealth; 2017; 5 (3); p. e26 | • Health communication and education • Service structure or model • Social media, apps and technology • Support services | • Multi-disciplinary and innovative PrEP care pathways | The study evaluated the usability and acceptability of a theory-based Android mobile phone app for HIV prevention. The content of the app was thought of positively overall, with 88% finding the level of detail and 81% finding the assessment recommendations to be useful or very useful. Additionally, 66% felt the app content helped them to stick to an HIV prevention plan. Most participants felt the app was a good balance of personal and professional language (71%) and the information was easy to understand (90%). Very few participants reported they would probably or definitely not download it again (5%), not recommend the app to a friend (3%), or not continue to use the app themselves (13%). |
| Sun, C., K. Anderson, D. Bangsberg, K. Toevs, D. Morrison, C. Wells, P. Clark and C. Nicolaidis | Access to HIV Pre-exposure Prophylaxis in Practice Settings: a Qualitative Study of Sexual and Gender Minority Adults’ Perspectives; Journal of General Intern Medicine; 2019 | • Level of knowledge • Prescribing habits and eligibility • Purview Paradox • Referral • Relationship with patient • Staffing and service allocation • Support services | • PrEP service aspects, settings and staff • PrEP prescriber experiences, therapeutic alliance and care planning • Multi-disciplinary and innovative PrEP care pathways | The study identifies barriers and facilitators of PrEP access by examining sexual and gender minority patients’ experiences with accessing health care systems and engaging with providers about PrEP in a variety of practice settings. Participants described the centrality of patient-provider relationships to positive experiences around PrEP, the necessity of personally advocating to access PrEP, and the experience of system-level barriers to PrEP access. Participants also made several suggestions to improve PrEP access including improving provider engagement with sexual and gender minority patients, encouraging providers to initiate conversations about PrEP, and increasing awareness of medication financial support. |
| Tangmunkongvorakul, A., S. Chariyalertsak, R. K. Amico, P. Saokhieo, V. Wannalak, T. Sangangamsakun, P. Goicochea and R. Grant | Facilitators and barriers to medication adherence in an HIV prevention study among men who have sex with men in the iPrEx study in Chiang Mai, Thailand; AIDS Care - Psychological and Socio-Medical Aspects of AIDS/HIV; 2013; 25 (8); pp. 961-967 | • Peer and social networks • Support services • Tailoring | • PrEP service aspects, settings and staff • PrEP adherence within formal service structures • Multi-disciplinary and innovative PrEP care pathways | The authors conducted the study to better understand the characteristics of iPrEx participants specifically from this underserved population in Thailand, and gain insights into their experiences of trying to take a daily tablet as part of this blinded PrEP trial. PrEP has the potential to transform HIV prevention interventions. Findings suggest that that participants held generally positive attitudes toward the iPrEx study and study medication and related this to high rates of adherence to the daily regimen. Participants also reflected on the provision of quality health care as part of participation in the trial, as well as support from clinical research staff, family and friends as helpful in supporting high rates of study medication adherence. Discourse concerning challenges to adherence included medication taking behavior, which was contextualized by lifestyle, living arrangement, social life, social stigma in terms of being mistakenly identified as HIVpositive or unintentional disclosure of sexual identity to family and friends, and relationship conflicts with partners. As PrEP use in HIV-negative individuals is in its nascent stages, further study is needed to explore risks and benefits, strategies to boost medication adherence, long-term effects, and how to best pair PrEP with STI prevention. |
| Tellalian, D., K. Maznavi, U. F. Bredeek and W. D. Hardy | Pre-Exposure Prophylaxis (PrEP) for HIV Infection: Results of a Survey of HIV Healthcare Providers Evaluating Their Knowledge, Attitudes, and Prescribing Practices; AIDS Patient Care & STDS; 2013; 27 (10); pp. 553-559 | • Health communication and education • Level of knowledge • Prescribing habits and eligibility • Purview Paradox • Staffing and service allocation | • PrEP service aspects, settings and staff • PrEP prescriber experiences, therapeutic alliance and care planning • PrEP adherence within formal service structures | The study evaluated provider knowledge, attitudes, perceptions, and prescribing practices regarding PrEP. Only 13% of respondents felt that PrEP was the ‘‘most effective’’ method for reducing HIV acquisition, while 47% felt that expanding HIV testing would be the most effective method. A scenario was provided regarding a healthy, 50-year- old heterosexual female in a monogamous relationship with an HIV-positive partner who does not use condoms. Sixty percent of respondents indicated that they would provide PrEP to a female patient in this setting. Twenty- six percent would not provide PrEP based on lack of scientific evidence, and 14% would not provide PrEP for other reasons. |
| Underhill, K., K. M. Morrow, C. M. Colleran, R. Holcomb, D. Operario, S. K. Calabrese, O. Galarraga and K. H. Mayer | Access to Healthcare, HIV/STI Testing, and Preferred Pre-Exposure Prophylaxis Providers among Men Who Have Sex with Men and Men Who Engage in Street-Based Sex Work in the US; Plos One; 2014; 9 (11) | • Health communication and education • Level of knowledge • Prescribing habits and eligibility • Referral • Relationship with patient • Service structure or model • Staffing and service allocation • Support services | • PrEP service aspects, settings and staff • PrEP prescriber experiences, therapeutic alliance and care planning • Multi-disciplinary and innovative PrEP care pathways | The study explored healthcare access, HIV/STI testing, unmet healthcare needs, and preferred PrEP providers. MSWs primarily accessed care in Emergency Departments, substance use clinics, correctional institutions, and walk-in clinics. Rates of HIV testing were high, but MSWs reported low access to other STI testing, low insurance coverage, and unmet healthcare needs including primary care, substance use treatment, and mental health services. MSM not engaging in sex work were more likely to report access to primary and specialist care. Rates of HIV testing among these MSM were slightly lower, but they reported more STI testing, more insurance coverage, and fewer unmet needs. Preferred PrEP providers for both groups included primary care physicians, infectious disease specialists, and psychiatrists. MSWs were also willing to access PrEP in substance use treatment and Emergency Department settings. |
| Vaccher, S. J., J. M. Kaldor, D. Callander, I. B. Zablotska and B. G. Haire | Qualitative Insights Into Adherence to HIV Pre-Exposure Prophylaxis (PrEP) Among Australian Gay and Bisexual Men; AIDS Patient Care & STDS; 2018; 32 (12); pp. 519-528 | • Peer and social networks • Relationship with patient • Service structure or model • Social media, apps and technology | • PrEP service aspects, settings and staff • PrEP prescriber experiences, therapeutic alliance and care planning • PrEP adherence within formal service structures • Multi-disciplinary and innovative PrEP care pathways | The article investigated factors that shaped PrEP users pill-taking experiences and explored the reasons these specific factors aided or diminished adherence. Participants portrayed PrEP in an overwhelmingly positive light, focusing on strategies that helped them maintain good adherence, despite other barriers they may have faced. There was an underlying sense of individuals taking ownership of their PrEP use, actively investigating different routines to determine the most suitable dosing strategy, and creating contingency plans if they were unable to access PrEP in their usual or preferred manner. Participants relied on various support networks and sought out information from a number of sources to ensure they had a good understanding of PrEP. Finally, while some participants reported side effects that led to the discontinuation of PrEP, or problems with disclosure that impacted on their ability to take PrEP as desired, the general consensus was that PrEP was broadly beneficial to their lives, and individuals would do all that they could to maintain high levels of adherence and take PrEP as required for the foreseeable future. |
| Witzel, T. C., W. Nutland and A. Bourne | What qualities in a potential HIV pre-exposure prophylaxis service are valued by black men who have sex with men in London? A qualitative acceptability study; International Journal of STD & AIDS; 2018; 29 (8); pp. 760-765 | • Relationship with patient • Service structure or model • Support services • Tailoring | • PrEP service aspects, settings and staff • PrEP prescriber experiences, therapeutic alliance and care planning | The authors sought to understand the dimensions of acceptability of a potential PrEP service for Black MSM in London. The findings highlighted the importance of proximity and anonymity; quality, efficiency and reassurance; and understanding, empathy and identity to participants. These relate, respectively, to preferences regarding clinic location and divisions from community, features of service delivery and staff characteristics. Careful consideration in regards to components used in service development will facilitate ongoing engagement. Interpersonal skills of staff are central to service acceptability, particularly when staff are perceived to be from similar cultural backgrounds as their patients. |

MSM – men who have sex with men; YMSM – young men who have sex with men; HCP – healthcare provider/professional; PCP – primary care provider; FSW – female sex worker; MSW – male sex worker; TG – transgender; TGW – transgender woman; M2F – male to female; F2M – female to male; CBC – community-based clinic; RCT – randomised control trial; PEP – post-exposure prophylaxis; GBM – gay and bisexual men; TPIP – Targeted PrEP Implementation Programme; iPrEX – implementation study; EMR – electronic medical records; iNSC – integrated Next Step Counselling; CRF – case report form; O2O – online to offline; NHS – National Health Service; IDU – intravenous drug user; NA – Neutral Assessment; STD – sexually transmitted disease; HB-PrEP – home based pre-exposure prophylaxis; CAS – condomless anal sex.
