# Supplementary material for: Pre-exposure Prophylaxis (PrEP) for HIV Prevention Among Men Who Have Sex with Men (MSM): A Scoping Review on PrEP Service Delivery and Programming
Source: AIDS Behav. 2020 Apr 9;24(11):3056–70. doi: 10.1007/s10461-020-02855-9 (PMC7502438; doi:10.1007/s10461-020-02855-9)
Supplement: Supplementary file 2 — Supplementary file2 (DOCX 43 kb) [file 10461_2020_2855_MOESM2_ESM.docx]

Supplementary Table S2 Supplemental Table of Included Studies: Study Detailsa

| **Authors** | **Title** | **Article type** | **Study details** | | | **HCP** | | **MSM** | | | **Geography** | | |
| --- | --- | --- | --- | --- | --- | --- | --- | --- | --- | --- | --- | --- | --- |
|  |  |  | **Method** | **Design** | **Recruited from** | **Number** | **Targeting** | **Number** | **Race** | **Age** | **Location** | **Urban/rural** | **Health system** |
| Adams, L. M. and B. H. Balderson | HIV providers' likelihood to prescribe pre-exposure prophylaxis (PrEP) for HIV prevention differs by patient type: a short report; AIDS Care; 2016; 28 (9); pp. 1154-1158 | Journal article | Quantitative | Cross sectional | Professional Association of HIV care providers | 260 prescribers | PrEP eligible MSM |  |  |  | US | Both | Private health insurance and public health coverage |
| Adams, L. M., B. H. Balderson, K. Brown, S. E. Bush and B. J. Packett | Who Starts the Conversation and Who Receives Preexposure Prophylaxis (PrEP)? A Brief Online Survey of Medical Providers’ PrEP Practices; Health Education & Behavior; 2018; 54 (5); 723-729 | Journal article | Quantitative | Cross sectional | Professional Association of HIV care providers | 342 prescribers | PrEP eligible MSM |  |  |  | US | Both | Private health insurance and public health coverage |
| Aloysius, I., A. Savage, J. Zdravkov, R. Korologou-Linden, A. Hill, R. Smith, V. Houghton-Price, M. Boffito and N. Nwokolo | InterPrEP. Internet-based pre-exposure prophylaxis with generic tenofovir DF/emtricitabine in London: an analysis of outcomes in 641 patients; Journal of Virus Eradication; 2017; 3 (4); pp. 218-222 | Journal article | Quantitative | Cross sectional | Clinic |  |  | 641 HIV negative | 81% White | 37 (32-45) years old | London, UK | Urban | Healthcare to all through NHS |
| Amico, K. R., V. McMahan, P. Goicochea, L. Vargas, J. L. Marcus, R. M. Grant and A. Liu | Supporting study product use and accuracy in self-report in the iPrEx study: next step counseling and neutral assessment; AIDS & Behavior; 2012; 16 (5); pp. 1243-1259 | Journal article | Qualitative | Intervention evaluation | Trial (iPrEx) | 37 study staff (20 counselors; 8 nurses; 9 study clinicians) | iPrEx staff using iNSC |  |  |  | Brazil; Ecuador; Peru; South Africa; Thailand; US | Both | Multiple |
| Amico, K. R., J. Miller, C. Balthazar, P. A. Serrano, J. Brothers, S. Zollweg and S. Hosek | Integrated Next Step Counseling (iNSC) for Sexual Health and PrEP Use Among Young Men Who Have Sex with Men: Implementation and Observations from ATN110/113; AIDS and Behavior; 2018 | Journal article | Qualitative | Intervention evaluation | Trial (iPrEx) |  |  | 178 | 10% White; 23% Hispanic/Latino; 67% Black/African American | 19 (15-22) years old | US | Both | Private health insurance and public health coverage |
| Anand, T., C. Nitpolprasert, D. Trachunthong, S. J. Kerr, S. Janyam, D. Linjongrat, L. B. Hightow-Weidman, P. Phanuphak, J. Ananworanich and N. Phanuphak | A novel Online-to-Offline (O2O) model for pre-exposure prophylaxis and HIV testing scale up; Journal of the International AIDS Society; 2017; 20 (1); p. 21326 | Journal article | Quantitative | Cross sectional | HIV educational website; eCounseling platforms; integrated social media networks |  |  | 272,568 | Not mentioned | 27 (23-33) years old | Thailand | Both | Universal healthcare through 3 government schemes |
| Arnold, E. A., P. Hazelton, T. Lane, K. A. Christopoulos, G. R. Galindo, W. T. Steward and S. F. Morin | A qualitative study of provider thoughts on implementing pre-exposure prophylaxis (PrEP) in clinical settings to prevent HIV infection; 2012; Plos One; 7 (7); e40603 | Journal article | Qualitative | Cross sectional | Referrals and snowball sampling | 22 PCPs (HIV specialists, community health clinic providers, and public health officials) | Seeing high numbers of MSM and TG women |  |  |  | California, US | Urban | Private health insurance and public health coverage |
| Arnold, T., L. Brinkley-Rubinstein, P. A. Chan, A. Perez-Brumer, E. S. Bologna, L. Beauchamps, K. Johnson, L. Mena and A. Nunn | Social, structural, behavioral and clinical factors influencing retention in Pre-Exposure Prophylaxis (PrEP) care in Mississippi; Plos One; 2017; 12 (2); p. e0172354; | Journal article | Qualitative | Cross sectional | PrEP program at LGBT clinic |  |  | 30 | 83% African American | 26.6 years old | Mississippi, US | Urban | Private health insurance and public health coverage |
| Beach, L. B., G. J. Greene, P. Lindeman, A. K. Johnson, C. N. Adames, M. Thomann, P. C. T. Washington and G. Phillips Ii | Barriers and Facilitators to Seeking HIV Services in Chicago Among Young Men Who Have Sex with Men: Perspectives of HIV Service Providers; AIDS Patient Care & STDS; 2018; 32 (11); pp. 468-476 | Journal article | Qualitative | Intervention evaluation | Department of Public Health | 19 key informants | YMSM |  |  |  | Illinois, US | Urban | Private health insurance and public health coverage |
| Bhatia, R., L. Modali, M. Lowther, N. Glick, M. Bell, S. Rowan, K. Keglovitz and J. Schneider | Outcomes of Preexposure Prophylaxis Referrals From Public STI Clinics and Implications for the Preexposure Prophylaxis Continuum; Sexually Transmitted Diseases; 2018; 45 (1); pp. 50-55 | Journal article | Quantitative | Retrospective cross sectional | Clinic |  |  | 137 | 7% Asian; 18% White; 32% Hispanic; 38% Black | 29 years old | Illinois, US | Urban | Private health insurance and public health coverage |
| Bien, C., V. Patel, O. Blackstock and U. Felsen | Reaching Key Populations: PrEP Uptake in an Urban Health Care System in the Bronx, New York; AIDS & Behavior; 2017; 5; pp. 1309-1314 | Journal article | Quantitative | Retrospective | HIV clinical cohort database |  |  | 108 | 29% Black; 36% Hispanic | 28 (IQR 24-37) years old | New York, US | Urban | Private health insurance and public health coverage |
| Bourne, A., B. Alba, A. Garner, G. Spiteri, A. Pharris and T. Noori | Use of, and likelihood of using, HIV pre-exposure prophylaxis among men who have sex with men in Europe and Central Asia: findings from a 2017 large geosocial networking application survey; Sexually Transmitted Infections; 2019 | Journal article | Quantitative | Cross sectional | Gay social network |  |  | 10, 562 | Not mentioned | All ages | France; Russia; UK; Italy; Turkey; Ukraine; Germany; Belgium; Belarus; Spain; Netherlands; Denmark; Switzerland; Portugal; Sweden; Other | Both | Multiple |
| Buttram, M. E. | The informal use of antiretroviral medications for HIV prevention by men who have sex with men in South Florida: initiation, use practices, medications and motivations; Culture, Health & Sexuality; 2018; 20 (11); pp. 1185-1198 | Journal article | Qualitative | Cross sectional | Health and social service agencies; LGBT community centre; community groups |  |  | 30 | 23% Black; 37% White; 40% Hispanic | 38 (18-62) years old | California, US | Urban | Private health insurance and public health coverage |
| Calabrese, S. K., M. Magnus, K. H. Mayer, D. S. Krakower, A. I. Eldahan, L. A. Gaston Hawkins, N. B. Hansen, T. S. Kershaw, K. Underhill, J. R. Betancourt and J. F. Dovidio | Putting PrEP into practice: Lessons learned from early-adopting U.S. providers' firsthand experiences providing HIV pre-exposure prophylaxis and associated care; Plos One; 2016; 11 (6) | Journal article | Mixed methods | Cross sectional | Purposive, targeted, email sampling | 18 HCPs who had prescribed PrEP | MSM; people who exchange sex; drug users; M2F TG |  |  |  | US | Both | Private health insurance and public health coverage |
| Chan, P. A., T. R. Glynn, C. E. Oldenburg, M. C. Montgomery, A. E. Robinette, A. Almonte, J. Raifman, L. Mena, R. Patel, K. H. Mayer, L. S. Beauchamps and A. S. Nunn | Implementation of Preexposure Prophylaxis for Human Immunodeficiency Virus Prevention Among Men Who Have Sex With Men at a New England Sexually Transmitted Diseases Clinic; Sexually Transmitted Diseases; 2016; 43 (11); pp. 717-723 | Journal article | Quantitative | Cross sectional | Clinic |  |  | 234 | 4% Asian; 9% Black/African American; 77% White | 32.3 (18-72) years old | Rhode Island, US | Urban | Publically funded |
| Clement, M. E., N. L. Okeke, T. Munn, M. Hunter, K. Alexis, A. Corneli, A. C. Seña, K. McGee and M. S. McKellar | Partnerships Between a University-Affiliated Clinic and Community-Based Organizations to Reach Black Men Who Have Sex With Men for PrEP Care; Journal of Acquired Immune Deficiency Syndromes; 2018; 77 (2); e25-e27 | Journal article | Quantitative | Retrospective cohort | Clinic |  |  | 91 (including GBM) | 5% Hispanic/Latino; 42% non-Hispanic/White; 46% non-Hispanic/Black | 31 (19-66) years old | North Carolina, US | Urban | Private health insurance and public health coverage |
| Clement, M. E., J. Seidelman, J. Wu, K. Alexis, K. McGee, N. L. Okeke, G. Samsa and M. S. McKellar | An educational initiative in response to identified PrEP prescribing needs among PCPs in the Southern U.S; AIDS Care; 2018; 30 (5); pp. 650-655 | Journal article | Quantitative | Cross sectional | Duke University Health System | 115 PCPs | PCPs running PrEP educational campaign |  |  |  | North Carolina, US | Urban | Private health insurance and public health coverage |
| Daughtridge, G. W., S. C. Conyngham, N. Ramirez and H. C. Koenig | I Am Men's Health: Generating Adherence to HIV Pre-Exposure Prophylaxis (PrEP) in Young Men of Color Who Have Sex with Men; Journal of the International Association of Providers of AIDS Care; 2015; 14 (2); pp. 103-107 | Journal article | Quantitative | Intervention cohort | Community program |  |  | 20 | 4% American Indian; 4% Asian; 9% White; 9% Hispanic/Latino; 57% Black/African American | 21 years old | Pennsylvania, US | Urban | Private health insurance and public health coverage |
| Desrosiers, A., M. Levy, A. Dright, M. Zumer, N. Jallah, I. Kuo, M. Magnus and M. Siegel | A Randomized Controlled Pilot Study of a Culturally-Tailored Counseling Intervention to Increase Uptake of HIV Pre-exposure Prophylaxis Among Young Black Men Who Have Sex with Men in Washington, DC; AIDS and Behavior; 2018; 23; pp. 105-115 | Journal article | Quantitative | RCT | Jack'd, Grindr, Tinder and Adam4Adam social networking applications |  |  | 50 HIV negative | Black/African American | 16-25 years old | Washington, US | Urban | Private health insurance and public health coverage |
| Doblecki-Lewis, S. and D. Jones | Community Federally Qualified Health Centers as Homes for HIV Preexposure Prophylaxis: Perspectives from South Florida; Journal of the International Association of Providers of AIDS Care; 2016; 15 (6); pp. 522-528 | Journal article | Qualitative | Cross sectional | HIV-servicing community health centres | 22 service providers, administrators or case managers | Recruited based on job role. At least one prescribing provider |  |  |  | Florida, US | Urban | Private health insurance and public health coverage |
| Doblecki-Lewis, S., A. Liu, D. Feaster, S. E. Cohen, G. Cardenas, O. Bacon, E. Andrew and M. A. Kolber | Healthcare Access and PrEP Continuation in San Francisco and Miami After the US PrEP Demo Project; Journal of Acquired Immune Deficiency Syndromes; 2017; 74 (5); pp. 531-538 | Journal article | Quantitative | Cross sectional | Trial (US PrEP Demo Project) |  |  | 173 former participants | Asian 2.9%; Black 9.9%; White 77.8% | All ages | California and Florida, US | Urban | Private health insurance and public health coverage |
| Dubov, A., L. Fraenkel, R. Yorick, A. Ogunbajo and F. L. Altice | Strategies to Implement Pre-exposure Prophylaxis with Men Who Have Sex with Men in Ukraine; AIDS & Behavior; 2018; 4; pp. 110-1112 | Journal article | Quantitative | Cross sectional | LGBT NGO mailing lists; advertisements on websites; social networking applications |  |  | 1,184 | Not mentioned | 28.6 years old | Ukraine | Both | Universal healthcare system |
| Eaton, L. A., D. D. Matthews, L. A. Bukowski, M. R. Friedman, C. J. Chandler, D. L. Whitfield, J. M. Sang and R. D. Stall | Elevated HIV Prevalence and Correlates of PrEP Use Among a Community Sample of Black Men who Have Sex with Men; Journal of Acquired Immune Deficiency Syndromes; 2018; 79; pp. 339-346 | Journal article | Quantitative | Cross sectional | Gay pride event |  |  | 4184 HIV negative | Black/African American | 34.7 years old | Pennsylvania; Michigan; Washington; Georgia; Texas; Tennesse, US | Urban | Private health insurance and public health coverage |
| Elst, E., J. Mbogua, D. Operario, G. Mutua, C. Kuo, P. Mugo, J. Kanungi, S. Singh, J. Haberer, F. Priddy and E. Sanders | High Acceptability of HIV Pre-exposure Prophylaxis but Challenges in Adherence and Use: Qualitative Insights from a Phase I Trial of Intermittent and Daily PrEP in At-Risk Populations in Kenya; AIDS & Behavior; 2013; 6; pp. 2162-2172 | Journal article | Qualitative | Cross sectional | Exploratory data from RCT |  |  | 51 (including FSW) | Black | 26 (18-46) years old | Kenya, Africa | Urban | Public healthcare |
| Fuchs, J. D., K. Stojanovski, E. Vittinghoff, V. M. McMahan, S. G. Hosek, K. R. Amico, A. Kouyate, H. J. Gilmore, S. P. Buchbinder, R. T. Lester, R. M. Grant and A. Y. Liu | A Mobile Health Strategy to Support Adherence to Antiretroviral Preexposure Prophylaxis; AIDS Patient Care & STDS; 2018; 32 (3); pp. 104-111 | Journal article | Mixed methods | Intervention evaluation | Trial (iPrEx) |  |  | 52 | Hispanic/Latino 11%; Black 13%; White 68% | 49 (21-66) years old | California and Illinois, US | Urban | Private health insurance and public health coverage |
| Galea, J. T., J. J. Kinsler, X. Salazar, S. J. Lee, M. Giron, J. N. Sayles, C. Cáceres, W. E. Cunningham, J. T. Galea, J. J. Kinsler, X. Salazar, S. J. Lee, M. Giron, J. N. Sayles, C. Cáceres and W. E. Cunningham | Acceptability of pre-exposure prophylaxis as an HIV prevention strategy: barriers and facilitators to pre-exposure prophylaxis uptake among at-risk Peruvian populations; International Journal of STD & AIDS; 2011; 22 (5); pp. 256-262 | Journal article | Qualitative | Cross sectional | Community outreach |  |  | 17 (also included 15 FSW and 13 M2F TG) | Not mentioned | 33 years old | Peru | Urban | Mixed health system: Ministry of Health, EsSalud, the Armed Forces (FFAA), National Police (PNP) and the private sector |
| Galindo, G. R., J. J. Walker, P. Hazelton, T. Lane, W. T. Steward, S. F. Morin and E. A. Arnold | Community member perspectives from transgender women and men who have sex with men on pre-exposure prophylaxis as an HIV prevention strategy: implications for implementation; Implementation Science; 2012; 7 (1); p. 116 | Journal article | Qualitative | Cross sectional | Community; word of mouth; flyers at social service groups; street |  |  | 30 HIV negative and unknown status (includes M2F TG) | 20% White; 33% Latino; 43% Black | 36.1 (21-58) years old | California, US | Urban | Private health insurance and public health coverage |
| Gilmore, H. J., A. Liu, K. A. Koester, K. R. Amico, V. McMahan, P. Goicochea, L. Vargas, D. Lubensky, S. Buchbinder and R. Grant | Participant Experiences and Facilitators and Barriers to Pill Use Among Men Who Have Sex with Men in the iPrEx Pre-Exposure Prophylaxis Trial in San Francisco; AIDS Patient Care & STDs; 2013; 27 (10); pp. 560-566 | Journal article | Qualitative | Cross sectional | Trial (iPrEx) |  |  | 52 | 7% Asian; 12% African American; 15% Hispanic/Latino; 66% White | 43 (22-66) years old | California, US | Urban | Private health insurance and public health coverage |
| Golub, S. A., K. E. Gamarel, H. J. Rendina, A. Surace and C. L. Lelutiu-Weinberger | From Efficacy to Effectiveness: Facilitators and Barriers to PrEP Acceptability and Motivations for Adherence Among MSM and Transgender Women in New York City; AIDS Patient Care & STDs; 2013; 27 (4); pp. 248-254 | Journal article | Mixed methods | Cross sectional | Trial (iPrEx) |  |  | 177 (including 7 M2F TG) | 21% Latino; 38% Black | 34.8 (18-58) years old | New York, US | Urban | Private health insurance and public health coverage |
| Grimm, J. and J. Schwartz | It's Like Birth Control for HIV: Communication and Stigma for Gay Men on PrEP; Journal of Homosexuality; 2018; pp. 2-20 | Journal article | Qualitative | Cross sectional | Advertisements on Grindr and Scruff |  |  | 39 HIV negative | 6% Middle East or North African; 19% Latino; 69% White | 35 years old | US | Both | Private health insurance and public health coverage |
| Grov, C. and N. Kumar | HIV Pre-Exposure Prophylaxis (PrEP) Is Coming to Europe, but Are Gay Men Ready to Accept It? Qualitative Findings from Berlin, Germany; Sexuality Research and Social Policy; 2018; 15 (3); pp. 283-289 | Journal article | Qualitative | Cross sectional | Referrals from HIV prevention/treatment and LGBT advocacy organizations; advertising on MSM sexual networking website; peer-to-peer referral |  |  | 20 (HIV negative and HIV positive) | Not mentioned | 35.9 (24-54) years old | Germany | Urban | Universal multi-payer health care system paid for by a combination of statutory and private health insurance |
| Hoffman, S., J. A. Guidry, K. L. Collier, J. E. Mantell, D. Boccher-Lattimore, F. Kaighobadi and T. G. M. Sandfort | A Clinical Home for Preexposure Prophylaxis: Diverse Health Care Providers' Perspectives on the "Purview Paradox"; Journal of the International Association of Providers of AIDS Care; 2016; 15 (1); pp. 59-65 | Journal article | Qualitative | Cross sectional | Co-investigators and members of expert panel initially contacted clinicians in their settings and networks | 30 PCPs and HIV specialists | MSM; IDUs; high risk heterosexual women and men |  |  |  | New York, US | Urban | Private health insurance and public health coverage |
| Hojilla, J. C. | Optimizing the delivery of HIV pre-exposure prophylaxis (PrEP): An evaluation of risk compensation, disengagement, and the PrEP cascade: HIV serodisclosure among MSM and transgender women on HIV PrEP; AAI10282719; 2018; pp. 11-32 | PhD | Quantitative | Retrospective | Trial (iPrEx) |  |  | 1,184 | Not mentioned | 30 years old | Brazil; Ecuador; Peru; South Africa; Thailand; USA | Both | Multiple |
| Hojilla, J. C. | Optimizing the delivery of HIV pre-exposure prophylaxis (PrEP): An evaluation of risk compensation, disengagement, and the PrEP cascade: Stimulant use is associated with PrEP disengagement in men who have sex with men and transgender women; AAI10282719; 2018; pp. 33-51 | PhD | Quantitative | Retrospective cross sectional | Trial (iPrEx) |  |  | 330 | 57% Latino/Hispanic | 29 (19-70) years old | Brazil; Ecuador; Peru; South Africa; Thailand; USA | Both | Multiple |
| Hojilla, J. C., D. Vlahov, P. Crouch, C. Dawson-Rose, K. Freeborn and A. Carrico | HIV Pre-exposure Prophylaxis (PrEP) Uptake and Retention Among Men Who Have Sex with Men in a Community-Based Sexual Health Clinic; AIDS & Behavior; 2018; 4; pp. 1096-1099 | Journal article | Quantitative | Retrospective cross sectional | Self-referred; offered PrEP by clinicians |  |  | 344 | 65% White; 74% non-Hispanic | 31 years old | California, US | Urban | Nurse-led community based clinic that provides free sexual health services |
| Hubach, R. D., J. M. Currin, C. A. Sanders, A. R. Durham, K. E. Kavanaugh, D. L. Wheeler and J. M. Croff | Barriers to Access and Adoption of Pre-Exposure Prophylaxis for the Prevention of HIV Among Men Who Have Sex With Men (MSM) in a Relatively Rural State; AIDS Education & Prevention; 2017; 29 (4); pp. 315-329 | Journal article | Qualitative | Cross sectional | Internet-based directed marketing; purposive approaches; electronic advertisements placed on a variety of social and sexual networking websites; flyers in LGBT venues |  |  | 20 (includes GBM) | Black/African American 5%; Native American/Alaskan 10%; White/non-Hispanic 80% | 36.4 (22-66) years old | Oklahoma, US | Rural | Private health insurance and public health coverage |
| Jaiswal, J., M. Griffin, S. N. Singer, R. E. Greene, I. L. Z. Acosta, S. K. Kaudeyr, F. Kapadia and P. N. Halkitis | Structural Barriers to Pre-exposure Prophylaxis Use Among Young Sexual Minority Men: The P18 Cohort Study; Current HIV Research; 2018; 16 (3); pp. 237-249 | Journal article | Quantitative | Longitudinal cohort | Trial |  |  | 492 | Asian 7.7%; White 25% ; Black 27%; Hispanic/Latino 32% | 22.47 years old | New York, US | Urban | Private health insurance and public health coverage |
| John, S. A., H. J. Rendina, C. Grov and J. T. Parsons | Home-based pre-exposure prophylaxis (PrEP) services for gay and bisexual men: An opportunity to address barriers to PrEP uptake and persistence; Plos One; 2017; 12 (12); pp. 1-14 | Journal article | Quantitative | Longitudinal cohort | Trial (One Thousand Strong) |  |  | 906 HIV negative (includes GBM) | Black 7%; Latino 12%; White 72% | 41.9 years old | US | Both | Private health insurance and public health coverage |
| Karris, M. Y., S. E. Beekmann, S. R. Mehta, C. M. Anderson and P. M. Polgreen | Are we prepped for preexposure prophylaxis (PrEP)? Provider opinions on the real-world use of PrEP in the United States and Canada; Clincial Infectious Diseases: An Official Publication of the Infectious Diseases of America; 2014; 58 (5); pp. 704-712 | Journal article | Quantitative | Cross sectional | Provider-based network of infectious disease physicians; Emerging Infections Network | 573 infectious disease physicians | Infectious diseases |  |  |  | US; Canada | Both | Private health insurance and public health coverage |
| Klassen, B. J., S. Y. Lin, N. J. Lachowsky, R. S. Hogg, D. M. Moore, E. A. Roth, J. B. Edward and S. A. Chown | Gay Men’s Understanding and Education of New HIV Prevention Technologies in Vancouver, Canada; Qualitative Health Research; 2017; 27 (12); pp. 1775-1791 | Journal article | Qualitative | Longitudinal cohort | Trial (Momentum Health Study) |  |  | 15 HIV negative | Not mentioned | 38 (22-58) years old | Vancouver, Canada | Urban | Provincial and territorial systems of publicly funded health care |
| Krakower, D. S., N. C. Ware, K. M. Maloney, I. B. Wilson, J. B. Wong and K. H. Mayer | Differing Experiences with Pre-Exposure Prophylaxis in Boston Among Lesbian, Gay, Bisexual, and Transgender Specialists and Generalists in Primary Care: Implications for Scale-Up; AIDS Patient Care & STDs; 2017; 31 (7); pp. 297-304 | Journal article | Qualitative | Cross sectional | Purposive sampling from a community health centre | 31 service providers and PCPs | LGBT patients |  |  |  | Massachusetts, US | Urban | Private health insurance and public health coverage |
| Kurtz, S. P. and M. E. Buttram | Misunderstanding of Pre-Exposure Prophylaxis Use Among Men Who Have Sex with Men: Public Health and Policy Implications; 2016; LGBT Health; 3 (6); pp. 461-464 | Journal article | Qualitative | Cross sectional | GPS-based social networking applications (e.g., Grindr, SCRUFF |  |  | 31 HIV negative/unknown status | 19% White; 23% African American/Black; 55% Hispanic | 27.2 years old | Florida, US | Urban | Private health insurance and public health coverage |
| Kwakwa, H. A., S. Bessias, D. Sturgis, G. Walton, R. Wahome, O. Gaye and M. Jackson | Engaging United States Black Communities in HIV Pre-exposure Prophylaxis: Analysis of a PrEP Engagement Cascade; Journal of National Medical Association; 2018; 110 (5); pp. 480-485 | Journal article | Quantitative | Cross sectional | Referrals from city health centre PrEP program |  |  | 785 (485 male) | 4% Hispanic; 8% White; 88% Black | 26 years old | Pennsylvania, US | Urban | Free primary care and HIV specialist services |
| Landovitz, R. J., M. Beymer, R. Kofron, K. R. Amico, C. Psaros, L. Bushman, P. L. Anderson, R. Flynn, D. P. Lee, R. K. Bolan, W. C. Jordan, C. Tseng, R. Dierst-Davies, J. Rooney and A. R. Wohl | Plasma Tenofovir Levels to Support Adherence to TDF/FTC Preexposure Prophylaxis for HIV Prevention in MSM in Los Angeles, California; Journal of Acquired Immune Deficiency Syndromes; 2017; 76 (5); pp. 501-511 | Journal article | Quantitative | Intervention cohort (PrEP-based and PEP based cohorts) | Trial (PATH-PrEP) |  |  | 329 (includes PEP users; 1 M2F TG) | 11% Black/African American; 28% Hispanic/Latino; 50% White | 34 (20-69) years old | California, US | Urban | Private health insurance and public health coverage |
| Lelutiu-Weinberger, C. and S. A. Golub | Enhancing PrEP Access for Black and Latino Men Who Have Sex With Men; Journal of Acquired Immune Deficiency Syndromes; 2016; 73 (5); pp. 547-555 | Journal article | Quantitative | Cross sectional | Flyers in local venues; placement of study ads on websites and mobile applications, e.g. Grindr, Adam4Adam, or Craigslist; outreach at bars, events, community-based organisations; participant referral |  |  | 491 | 23% Latino; 33% Black/African American; 37% White | All ages | New York, US | Urban | Private health insurance and public health coverage |
| Levy, M. E., C. C. Watson, S. N. Glick, I. Kuo, L. Wilton, R. A. Brewer, S. D. Fields, V. Criss and M. Magnus | Receipt of HIV prevention interventions is more common in community-based clinics than in primary care or acute care settings for Black men who have sex with men in the District of Columbia; AIDS Care - Psychological and Socio-Medical Aspects of AIDS/HIV; 2016; 28 (5); pp. 660-664 | Journal article | Quantitative | Cross sectional | PrEP project (Pursuing Real and Innovative Ideas to Remove Structural Barriers for Men) |  |  | 75 | Black | 26 (18-60) years old | Washington, US | Urban | Private health insurance and public health coverage |
| Liu, A. Y., E. Vittinghoff, P. von Felten, K. R. Amico, P. L. Anderson, R. Lester, E. Andrew, I. Estes, P. Serrano, J. Brothers, S. Buchbinder, S. Hosek and J. D. Fuchs | Randomized Controlled Trial of a Mobile Health Intervention to Promote Retention and Adherence to Pre-exposure Prophylaxis among Young People at Risk for Human Immunodeficiency Virus: The EPIC Study; Clinical Infectious Diseases: An Official Publication of the Infectious Diseases Society of America; 2018 | Journal article | Quantitative | RCT (EPIC study - PrEPmate vs Standard of Care arms) | Ruth M. Rothstein CORE Center, a public health clinic focused on HIV prevention, care, and research |  |  | 121 | 28% Black; 36% Latino | 24.2 (18-29) years old | Illinois, US | Urban | Private health insurance and public health coverage |
| Maloney, K. M., D. S. Krakower, D. Ziobro, J. G. Rosenberger, D. Novak and K. H. Mayer | Culturally Competent Sexual Healthcare as a Prerequisite for Obtaining Preexposure Prophylaxis: Findings from a Qualitative Study; LGBT Health; 2017; 4 (4); pp. 310-314 | Journal article | Qualitative | Cross sectional | Sexual networking website |  |  | 24 | 13% Hispanic/Latino; 88% White | 48 (40-52) years old | US | Both | Private health insurance and public health coverage |
| Marcus, J. L., L. B. Hurley, C. B. Hare, D. P. Nguyen, T. Phengrasamy, M. J. Silverberg, J. E. Stoltey and J. E. Volk | Preexposure Prophylaxis for HIV Prevention in a Large Integrated Health Care System: Adherence, Renal Safety, and Discontinuation; JAIDS: Journal of Acquired Deficiency Syndrome; 2016; 73 (5); pp. 540-546 | Journal article | Quantitative | Cohort | Clinic |  |  | 972 | 4% Black; 10% Asian; 12% Hispanic; 70% White | 37.5 (18-68) years old | California, US | Both | Integrated health care system |
| Marcus, J. L., K. Levine, C. Grasso, D. S. Krakower, V. Powell, K. T. Bernstein, S. Boswell and K. H. Mayer | HIV Preexposure Prophylaxis as a Gateway to Primary Care; American Journal of Public Health; 2018; 108 (10); pp. 1418-1420 | Journal article | Quantitative | Cross sectional | Clinic |  |  | 5,857 (2,047 PrEP users) | Black 5%; Asian 6%; Hispanic 14% | 33 years old | Boston, Massachusetts | Urban | Private health insurance and public health coverage |
| Marks, S. J., R. C. Merchant, M. A. Clark, T. Liu, J. G. Rosenberger, J. Bauermeister and K. H. Mayer | Potential Healthcare Insurance and Provider Barriers to Pre-Exposure Prophylaxis Utilization Among Young Men Who Have Sex with Men; AIDS Patient Care & STDs; 2017; 31 (11); pp. 470-478 | Journal article | Quantitative | Cross sectional | Social media |  |  | 1,197 HIV negative (80 PrEP users) | 12% Black; 29% Hispanic; 39% White | 18-24 years old | US | Both | Private health insurance and public health coverage |
| Mayer, K., S. Safren, S. Elsesser, C. Psaros, J. Tinsley, M. Marzinke, W. Clarke, C. Hendrix, S. Wade Taylor, J. Haberer and M. Mimiaga | Optimizing Pre-Exposure Antiretroviral Prophylaxis Adherence in Men Who Have Sex with Men: Results of a Pilot Randomized Controlled Trial of 'Life-Steps for PrEP'; AIDS & Behavior; 2017; 5; pp. 1350-1360 | Journal article | Quantitative | RCT | Community outreach; advertisements; flyers; social media; clinic |  |  | 39 | 2% Black/African American; 8% Hispanic/Latino; 86% White | 38.4 (25-50) years old | New England (Maine, Vermont, New Hampshire, Massachusetts, Rhode Island, Connecticut), US | Both | Private health insurance and public health coverage |
| Merchant, R. C., D. Corner, E. Garza, W. Guan, K. H. Mayer, L. Brown and P. A. Chan | Preferences for HIV pre-exposure prophylaxis (PrEP) information among men-who-have-sex-with-men (MSM) at community outreach settings; Journal of Gay & Lesbian Mental Health; 2016; 20 (1); pp. 21-33 | Journal article | Quantitative | Cross sectional | Gay pride event; bars and nightclubs |  |  | 209 | 1% Asian; 8% Black/African American; 10% Hispanic; 75% White | 30 (24-44) years old | Rhode Island, US | Urban | Private health insurance and public health coverage |
| Mitchell, J. T., S. LeGrand, L. B. Hightow-Weidman, M. S. McKellar, A. D. M. Kashuba, M. Cottrell, T. McLaurin, G. Satapathy and F. J. McClernon | Smartphone-Based Contingency Management Intervention to Improve Pre-Exposure Prophylaxis Adherence: Pilot Trial; JMIR Mhealth and Uhealth; 2018; 6 (9); p. e10456 | Journal article | Mixed methods | RCT | Community advertisements; word of mouth |  |  | 10 | 20% Asian; 70% White | 24.1 years old | US | Both | Private health insurance and public health coverage |
| Mullins, T. L. K., G. Zimet, M. Lally, J. Xu, S. Thornton and J. A. Kahn | HIV Care Providers' Intentions to Prescribe and Actual Prescription of Pre-Exposure Prophylaxis to At-Risk Adolescents and Adults; AIDS Patient Care & STDs; 2017; 31 (12); pp. 504-516 | Journal article | Mixed methods | Cross sectional | Research network (National Institutes of Health funded Adolescent Medicine Trials Network for HIV/AIDS Interventions (ATN)) | 56 clinicians | Provided care to HIV infected youth |  |  |  | US | Both | Private health insurance and public health coverage |
| Mutua, G., E. Sanders, P. Mugo, O. Anzala, J. E. Haberer, D. Bangsberg, B. Barin, J. F. Rooney, D. Mark, P. Chetty, P. Fast and F. H. Priddy | Safety and adherence to intermittent pre-exposure prophylaxis (PrEP) for HIV-1 in African men who have sex with men and female sex workers; Plos On; 2012; 7 (4); p. e33103 | Journal article | Quantitative | RCT | Centres that provide comprehensive HIV prevention package to at-risk research volunteers |  |  | 72 (includes FSW) | Black | 26 (18-46) years old | Kenya, Africa | Both | Public healthcare |
| Newman, P. A., A. Guta, A. Lacombe-Duncan and S. Tepjan | Clinical exigencies, psychosocial realities: negotiating HIV pre-exposure prophylaxis beyond the cascade among gay, bisexual and other men who have sex with men in Canada; Journal of the International AIDS Society; 2018; 21 (11); p. e25211 | Journal article | Qualitative | Exploratory cross sectional | Flyers; community outreach; word of mouth |  |  | 29 (GBM or PrEP user) | 21% 'person of colour'; 79% White | 36.7 years old | Toronto, Canada | Both | Provincial and territorial systems of publicly funded health care |
| Ojikutu, B., L. Bogart, K. Mayer, T. Stopka, P. Sullivan and Y. Ransome | Spatial Access and Willingness to Use Pre-Exposure Prophylaxis Among Black/African American Individuals in the United States: Cross-Sectional Survey; JMIR Public Health and Surveillance; 2019; 5 (1) | Journal article | Quantitative | Cross sectional | Probability-based, online, nonvolunteer Web panel |  |  | 787 | Black/African American | 34 years old | US | Both | Private health insurance and public health coverage |
| Ojile, N., D. Sweet and K. J. Kallail | A Preliminary Study of the Attitudes and Barriers of Family Physicians to Prescribing HIV Preexposure Prophylaxis; Kansas Journal of Medicine; 2017; 10 (2); pp. 40-42 | Journal article | Quantitative | Observational cross sectional | Email through practice based research network of family physicians | 20 service providers | Prescribing PrEP to high risk individuals |  |  |  | Kansas, US | Both | Private health insurance and public health coverage |
| Paparini, S., W. Nutland, T. Rhodes, V. Nguyen and J. Anderson | DIY HIV prevention: Formative qualitative research with men who have sex with men who source PrEP outside of clinical trials; Plos One; 2018; 13 (8); p. e0202830 | Journal article | Qualitative | Cross sectional | Advertisements on social networking applications |  |  | 20 | 0.5% Black/African; 1% Indian; 80% White | 29-56 years old | London, UK | Urban | Healthcare to all through NHS |
| Parisi, D., B. Warren, S. J. Leung, T. Akkaya-Hocagil, Q. Qin, I. Hahn and L. Stevens | A Multicomponent Approach to Evaluating a Pre-exposure Prophylaxis (PrEP) Implementation Program in Five Agencies in New York; JANAC; Journal of the Association of Nurses in AIDS Care; 2018; 29 (1) | Journal article | Quantitative | Intervention evaluation | TPIP |  |  | 171 (includes M2F TG) | 9% Asian; 13% Hispanic; 15% Black; 60% White | All ages | New York, US | Urban | Private health insurance and public health coverage |
| Parsons, J. T., S. A. John, T. H. F. Whitfield, J. Cienfuegos-Szalay and C. Grov | HIV/STI counseling and testing services received by gay and bisexual men using pre-exposure prophylaxis (PrEP) at their last PrEP care visit; Sexually Transmitted Diseases; 2018; 45 (12); pp. 798-802 | PhD | Quantitative | Cross sectional | Advertising; gay concentrated neighborhoods and settings (e.g., gay bars, pride events, at LGBT communitybased venues); digital recruitment (gay hookup websites and apps); social media |  |  | 104 GBM | 13% Black; 27% Latino; 52% White | 32.5 (21-61) years old | New York, US | Urban | Private health insurance and public health coverage |
| Pasipanodya, E. C., S. Jain, X. Sun, J. Blumenthal, E. Ellorin, K. Corado, M. P. Dube, E. S. Daar, S. R. Morris and D. J. Moore | Trajectories and Predictors of Longitudinal Preexposure Prophylaxis Adherence Among Men Who Have Sex With Men; Journal of Infectious Diseases; 2018; 218 (10); pp. 1551-1559 | Journal article | Quantitative | RCT | Clinic |  |  | 181 | 15% Black; 81% White | 34.98 years old | California, US | Both | Private health insurance and public health coverage |
| Patel, R. R., P. A. Chan, L. C. Harrison, K. H. Mayer, A. Nunn, L. A. Mena and W. G. Powderly | Missed Opportunities to Prescribe HIV Pre-Exposure Prophylaxis by Primary Care Providers in Saint Louis, Missouri; LGBT Health; 2018; 5 (4); pp. 250-256 | Journal article | Quantitative | Cross sectional | Clinic |  |  | 102 | 3% Asian; 3% Hispanic/Latino; 31% Black; 58% White | 29 (25-34) years old | Missouri, US | Urban | Private health insurance and public health coverage |
| Phanuphak, N., T. Sungsing, J. Jantarapakde, S. Pengnonyang, D. Trachunthong, P. Mingkwanrungruang, W. Sirisakyot, P. Phiayura, P. Seekaew, P. Panpet, P. Meekrua, N. Praweprai, F. Suwan, S. Sangtong, P. Brutrat, T. Wongsri, P. R. Na Nakorn, S. Mills, M. Avery and R. Vannakit | Princess PrEP program: the first key population-led model to deliver pre-exposure prophylaxis to key populations by key populations in Thailand; Sexual Health; 2018; 15 (6); pp. 542-555 | Journal article | Quantitative | Intervention evaluation | Community Health Centres |  |  | 1,697 (1,467 MSM) | 95% Thai | 28.8 years old | Thailand | Rural | Universal health coverage in public funded health facilities |
| Philbin, M. M., C. M. Parker, R. G. Parker, P. A. Wilson, J. Garcia and J. S. Hirsch | Gendered Social Institutions and Preventive Healthcare Seeking for Black Men Who Have Sex with Men: The Promise of Biomedical HIV Prevention; Archives of Sexual Behavior; 2018; 7; pp. 2091-2100 | Journal article | Qualitative | Ethnographic | Clinic | 17 community stakeholders (outreach workers, community mobilizers, healthcare professionals) | HIV prevention and/or BMSM health | 31 | Black | 29 years old | New York, US | Urban | Private health insurance and public health coverage |
| Raifman, J., A. Nunn, C. E. Oldenburg, M. C. Montgomery, A. Almonte, A. L. Agwu, R. Arrington‐Sanders, P. A. Chan and R. Arrington-Sanders | An Evaluation of a Clinical Pre-Exposure Prophylaxis Education Intervention among Men Who Have Sex with Men; Health Services Research; 2018; 53 (4); 2249-2267 | Journal article | Quantitative | Intervention evaluation | Self-reported data on PrEP awareness and use from STD clinic intake forms |  |  | 316 | 20% White; 34% Latino; 50% Black | All ages | Rhode Island, US | Both | Private health insurance and public health coverage |
| Raifman, J. R. G., C. Flynn and D. German | Healthcare Provider Contact and Pre-exposure Prophylaxis in Baltimore Men Who Have Sex With Men; American Journal of Preventive Medicine; 2017; 52 (1); 55-63 | Journal article | Quantitative | Cross sectional | Baltimore MSM National HIV Behavioral Surveillance data |  |  | 401 | Hispanic 5%; White 25%; Black 60% | 34 years old | Maryland, US | Urban | Private health insurance and public health coverage |
| Refugio, O. N., M. M. Kimble, C. L. Silva, J. E. Lykens, C. Bannister and J. D. Klausner | PrEPTECH: a telehealth-based initiation program for human immunodeficiency virus pre-exposure prophylaxis in young men of color who have sex with men. A pilot study of feasibility; Journal of Acquired Immune Deficiency Syndromes; 2018; 80; pp. 40-45 | Journal article | Quantitative | Longitudinal cross sectional | Grindr; posters; flyers at gay venues; word of mouth |  |  | 25 HIV negative YMSM | Black/African American 8%; White 16%; Asian 32%; Hispanic/Latino 40% | 22 (18-25) years old | California, US | Urban | Cost-free PrEP services through telehealth |
| Ridgway, J. P., E. A. Almirol, A. Bender, A. Richardson, J. Schmitt, E. Friedman, N. Lancki, I. Leroux, N. Pieroni, J. Dehlin and J. A. Schneider | Which Patients in the Emergency Department Should Receive Preexposure Prophylaxis? Implementation of a Predictive Analytics Approach; AIDS Patient Care and STDs; 2018; 32 (5); pp. 202-207 | Journal article | Quantitative | Cross sectional | EMR from Emergency Departments |  |  | 164 potential candidates for HIV prevention services | Latino 4%; White 4%; Black/African American 90% | 25.4 years old | Illinois, US | Urban | Private health insurance and public health coverage |
| Rivierez, I., G. Quatremere, B. Spire, J. Ghosn and D. Rojas Castro | Lessons learned from the experiences of informal PrEP users in France: results from the ANRS-PrEPage study; AIDS Care; 2018; 30; pp. 48-53 | Journal article | Qualitative | Cross sectional | Community based organisations; social media |  |  | 24 | Not mentioned | 38.5 (30-43) years old | France | Both | Universal health care largely financed by government national health insurance |
| Rusie, L. K., C. Orengo, D. Burrell, A. Ramachandran, M. Houlberg, K. Keglovitz, D. Munar and J. A. Schneider | Preexposure Prophylaxis Initiation and Retention in Care Over 5 Years, 2012-2017: Are Quarterly Visits Too Much?; Clinical Infectious Diseases; 2018; 67 (2); 283-287 | Journal article | Quantitative | Cross sectional | Clinic |  |  | 5,583 | Asian 6%; Black 16%; Hispanic 16%; White 58% | All ages | Illinois, US | Urban | Private health insurance and public health coverage |
| Shover, C. L., M. Javanbakht, S. Shoptaw, R. K. Bolan, L. Sung-Jae, J. T. Parsons, J. Rendina and P. M. Gorbach | HIV Preexposure Prophylaxis Initiation at a Large Community Clinic: Differences Between Eligibility, Awareness, and Uptake; American Journal of Public Health; 2018; 108 (10); pp. 1409-1417 | Journal article | Quantitative | Cross sectional | Community clinic (The Los Angeles LGBT Center) |  |  | 19,875 (includes TG) | Black/African American 7%; Asian 9%; Hispanic/Latino 32%; White 42% | All ages | California, US | Urban | Free and low-cost HIV and STI testing |
| Siegler, A. J., A. Bratcher, K. M. Weiss, F. Mouhanna, L. Ahlschlager and P. S. Sullivan | Location location location: an exploration of disparities in access to publicly listed pre-exposure prophylaxis clinics in the United States; Annals of Epidemiology; 2018 | Journal article | Quantitative | Cross sectional | National database (PrEP Locator) |  |  | 2,094 | Differences across counties and States discussed | Not mentioned | US | Both | Private health insurance and public health coverage |
| Smith, D. K., L. Toledo, D. J. Smith, M. A. Adams and R. Rothenberg | Attitudes and Program Preferences of African-American Urban Young Adults About Pre-Exposure Prophylaxis (PrEP); AIDS Educaiton & Prevention; 2012; 24 (5); pp. 408-421 | Journal article | Qualitative | Cross sectional | Ongoing study |  |  | 19 (also includes 58 mixed gender) | African American | 21 (18-24) years old | Georgia, US | Urban | Private health insurance and public health coverage |
| Spinelli, M. A., H. M. Scott, E. Vittinghoff, A. Y. Liu, A. Morehead-Gee, R. Gonzalez and S. P. Buchbinder | Provider adherence to pre-exposure prophylaxis monitoring guidelines in a large primary care network; Open Forum Infectious Diseases; 2018; 5 (6) | Journal article | Quantitative | Cross sectional | Clinic |  |  | 405 | Asian 8%; African American 13%; 26% Latino; 36% White | 34 years old | California, US | Urban | Private health insurance and public health coverage |
| Stekler, J. D., V. McMahan, L. Ballinger, L. Viquez, F. Swanson, J. Stockton, B. Crutsinger-Perry, D. Kern and J. D. Scott | HIV Pre-exposure Prophylaxis Prescribing Through Telehealth; Journal of Acquired Immune Deficiency Syndromes; 2018; 77 (5); pp. e40-e42 | Letter to Editor | Quantitative | Cross sectional | Clinic telephone and email inquiries; other community-based organizations; following HIV testing appointments |  |  | 48 (10 telehealth participants; 38 Gay City PrEP clients) | Telehealth: 20% White, 20% Asian, 30% Hispanic; Gay City PrEP clients: 5% Asian, 29% Hispanic, 42% White | Telehealth: 30 (20-38) years old; Gay City PrEP clients: 27 (19-46) years old | California, US | Urban | Private health insurance and public health coverage |
| Sullivan, P. S., R. Driggers, J. D. Stekler, A. J. Siegler, T. Goldenberg, S. J. McDougal, J. Caucutt, J. Jones and R. Stephenson | Usability and Acceptability of a Mobile Comprehensive HIV Prevention App for Men Who Have Sex With Men: A Pilot Study; JMIR Mhealth and Uhealth; 2017; 5 (3); p. e26 | Journal article | Mixed methods | Intervention evaluation | Social media; sexual networking mobile phone app |  |  | 121 | Hispanic/Latino 8%; Asian 10%; Black/African American 21%; White 52% | 28 (24-34) years old | Georgia; California, US | Urban | Private health insurance and public health coverage |
| Sun, C., K. Anderson, D. Bangsberg, K. Toevs, D. Morrison, C. Wells, P. Clark and C. Nicolaidis | Access to HIV Pre-exposure Prophylaxis in Practice Settings: a Qualitative Study of Sexual and Gender Minority Adults’ Perspectives; Journal of General Intern Medicine; 2019 | Journal article | Qualitative | Cross sectional | AIDS service organizations; LGBTQcommunity centers; local health departments; word of mouth; print flyers; social media |  |  | 27 (sexual and gender minorities) | Latino 19%; White 70% | 38 (21-67 years old) | Oregon, US | Both | Private health insurance and public health coverage |
| Tangmunkongvorakul, A., S. Chariyalertsak, R. K. Amico, P. Saokhieo, V. Wannalak, T. Sangangamsakun, P. Goicochea and R. Grant | Facilitators and barriers to medication adherence in an HIV prevention study among men who have sex with men in the iPrEx study in Chiang Mai, Thailand; AIDS Care - Psychological and Socio-Medical Aspects of AIDS/HIV; 2013; 25 (8); pp. 961-967 | Journal article | Qualitative | Cross sectional | Trial (iPrEx) |  |  | 14 (focus groups); 32 (interviews); both HIV negative | Not mentioned | 19-37 years old | Chiang Mai, Thailand | Urban | Universal health coverage in public funded health facilities |
| Tellalian, D., K. Maznavi, U. F. Bredeek and W. D. Hardy | Pre-Exposure Prophylaxis (PrEP) for HIV Infection: Results of a Survey of HIV Healthcare Providers Evaluating Their Knowledge, Attitudes, and Prescribing Practices; AIDS Patient Care & STDS; 2013; 27 (10); pp. 553-559 | Journal article | Quantitative | Cross sectional | Research network (American Academy of HIV Medicine) | 189 HIV HCPs | Prescribing PrEP to high risk individuals |  |  |  | US | Both | Private health insurance and public health coverage |
| Underhill, K., K. M. Morrow, C. M. Colleran, R. Holcomb, D. Operario, S. K. Calabrese, O. Galarraga and K. H. Mayer | Access to Healthcare, HIV/STI Testing, and Preferred Pre-Exposure Prophylaxis Providers among Men Who Have Sex with Men and Men Who Engage in Street-Based Sex Work in the US; Plos One; 2014; 9 (11) | Journal article | Qualitative | Cross sectional | Outreach in entertainment venues, sex work venues; community-based organizations; clinics;advertising in local media serving MSM |  |  | 38 (focus groups - 17 MSM); 26 (interviews - 25 MSM); includes MSWs | MSM focus groups: 12% Hispanic/Latino, 29% African American, 71% White; MSM interviews: 4% Asian, 12% African American, 24% Hispanic/Latino, 76% White | MSM focus groups: 39 (27-61) years old; MSM interviews: 33 (21-70) years old | Rhode Island, US | Urban | Private health insurance and public health coverage |
| Vaccher, S. J., J. M. Kaldor, D. Callander, I. B. Zablotska and B. G. Haire | Qualitative Insights Into Adherence to HIV Pre-Exposure Prophylaxis (PrEP) Among Australian Gay and Bisexual Men; AIDS Patient Care & STDS; 2018; 32 (12); pp. 519-528 | Journal article | Qualitative | Cross sectional | Advertisements on online social networking sites; contacting participants in PrEP implementation studies who had consented to be contacted for other research |  |  | 24 HIV negative GBM | Not mentioned | 38 (18-53) years | Sydney, Australia | Urban | Public healthcare |
| Witzel, T. C., W. Nutland and A. Bourne | What qualities in a potential HIV pre-exposure prophylaxis service are valued by black men who have sex with men in London? A qualitative acceptability study; International Journal of STD & AIDS; 2018; 29 (8); pp. 760-765 | Journal article | Qualitative | Cross sectional | Gay-specific geolocation social networking applications (apps); social media; PROUD study mailing list through the Medical Research Council Clinical Trials Unit |  |  | 25 | Black | 31.1 (18-45) years old | London, UK | Urban | Healthcare to all through NHS |

MSM – men who have sex with men; YMSM – young men who have sex with men; HCP – healthcare provider/professional; PCP – primary care provider; FSW – female sex worker; MSW – male sex worker; TG – transgender; TGW – transgender woman; M2F – male to female; F2M – female to male; CBC – community-based clinic; RCT – randomised control trial; PEP – post-exposure prophylaxis; GBM – gay and bisexual men; TPIP – Targeted PrEP Implementation Programme; iPrEX – implementation study; EMR – electronic medical records; iNSC – integrated Next Step Counselling; CRF – case report form; O2O – online to offline; NHS – National Health Service; IDU – intravenous drug user; NA – Neutral Assessment; STD – sexually transmitted disease; HB-PrEP – home based pre-exposure prophylaxis; CAS – condomless anal sex.
